# Supplementary figures and images for: Predicting online participation through Bayesian network analysis
Source: PLoS One. 2021 Dec 23;16(12):e0261663. doi: 10.1371/journal.pone.0261663 (PMC8699968; doi:10.1371/journal.pone.0261663)

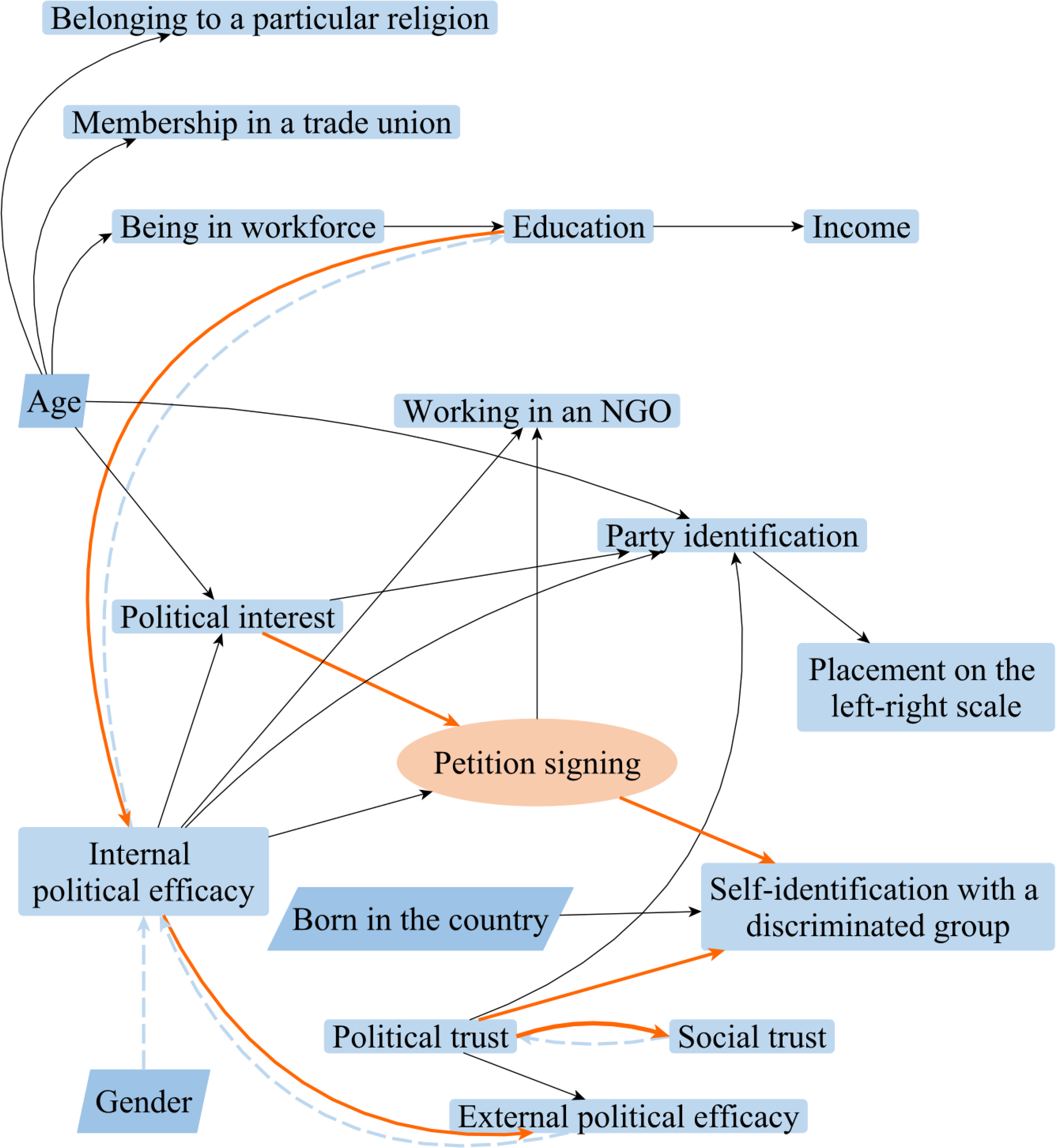

Supplement: S1 Fig — Source: [35]. N = 27 366 individuals in 19 countries. Notes: Within Bayesian network analysis, score-based Tabu and hybrid H2PC algorithms were applied to analyze the data and learn the structure of the causal relationships between the variables. Dashed blue lines represent false positives, i.e., edges that are not present in the structure learned by the Tabu algorithm but present in the structure learned by H2PC. Orange lines represent false negatives, i.e., edges that are present in the structure learned by the Tabu algorithm but absent in the structure learned by H2PC. All the edges from the other nodes to “Age”, “Gender” and “Born in the country” are blacklisted prior to learning the structure. In the figure, those nodes that can only be parents have a darker blue color. The node “Country” (i.e., the country of the respondent’s residency) is present in the structure but not depicted by the figure to facilitate the apprehension of the relationships between the nodes of interest. All variables are individual-level variables. (TIF) [file pone.0261663.s001.tif]

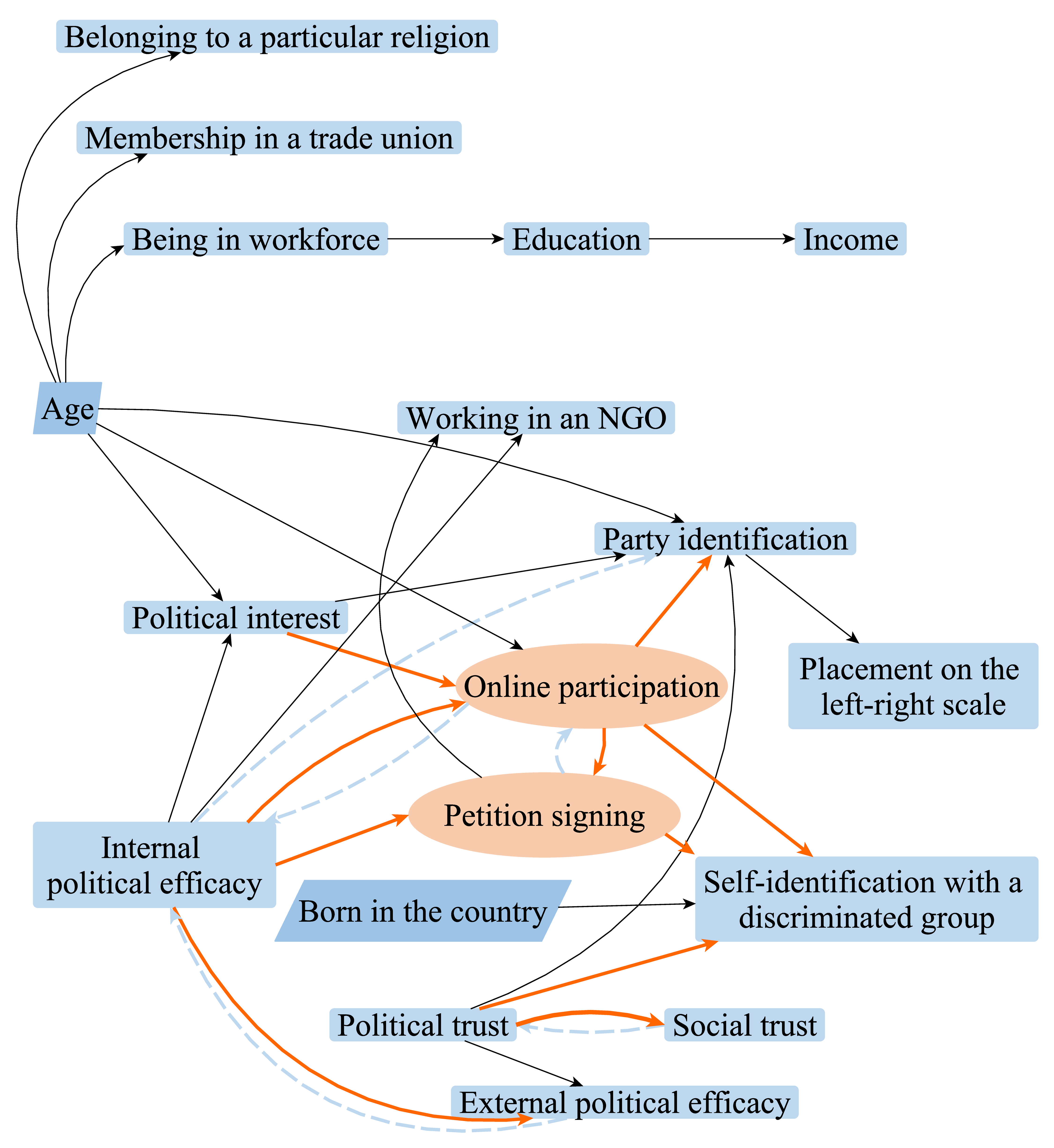

Supplement: S2 Fig — Source: [35]. N = 27 323 individuals in 19 countries. Notes: Within Bayesian network analysis, score-based Tabu and hybrid H2PC algorithms were applied to analyze the data and learn the structure of the causal relationships between the variables. Dashed blue lines represent false positives, i.e., edges that are not present in the structure learned by the Tabu algorithm but present in the structure learned by H2PC. Orange lines represent false negatives, i.e., edges that are present in the structure learned by the Tabu algorithm but absent in the structure learned by H2PC. All the edges from the other nodes to “Age”, “Gender” and “Born in the country” are blacklisted prior to learning the structure. In the figure, those nodes that can only be parents have a darker blue color. The node “Country” (i.e., the country of the respondent’s residency) is present in the structure but not depicted by the figure to facilitate the apprehension of the relationships between the nodes of interest. All variables are individual-level variables. (TIF) [file pone.0261663.s002.tif]

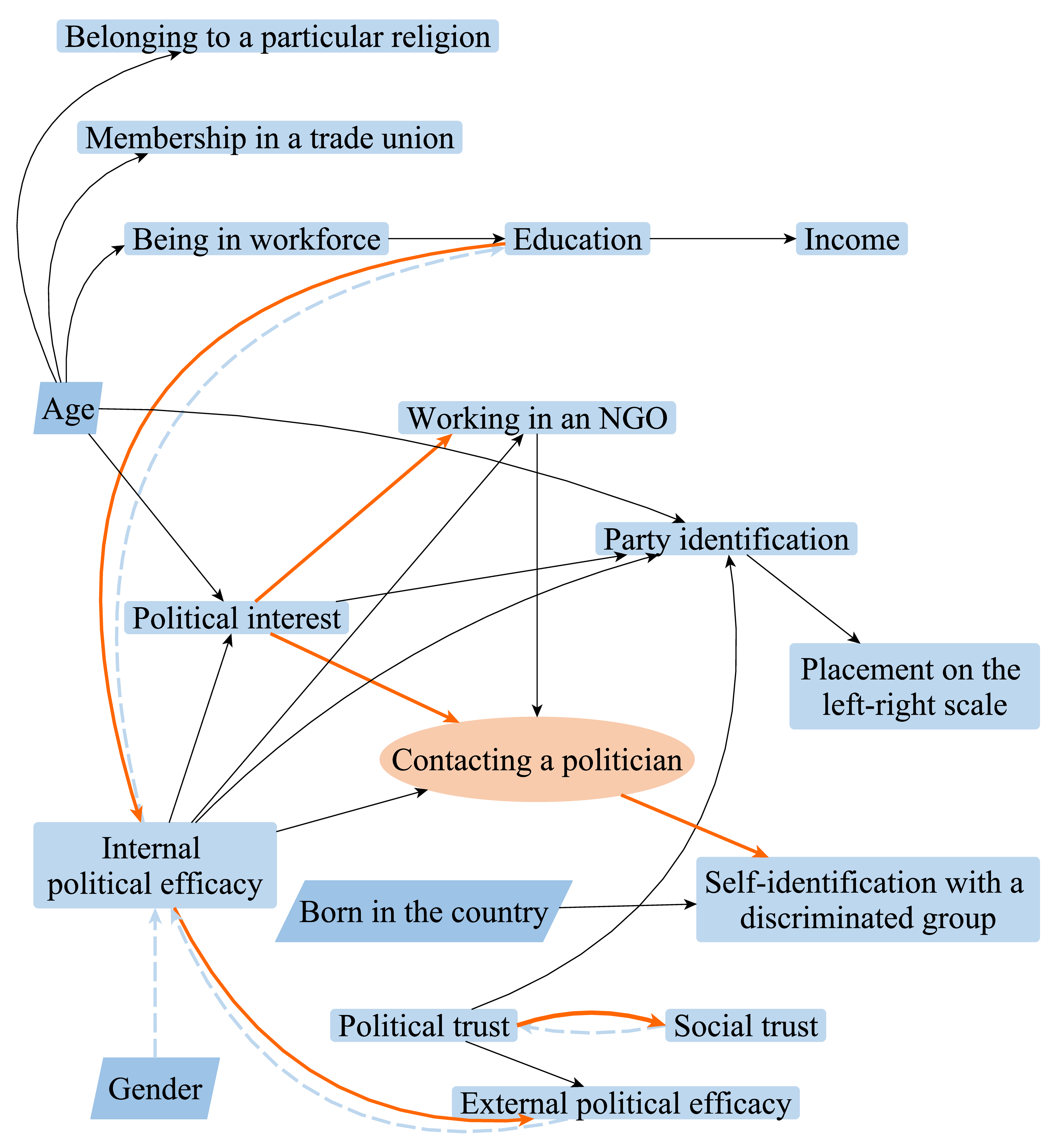

Supplement: S3 Fig — Source: [35]. N = 27 397 individuals in 19 countries. Notes: Within Bayesian network analysis, score-based Tabu and hybrid H2PC algorithms were applied to analyze the data and learn the structure of the causal relationships between the variables. Dashed blue lines represent false positives, i.e., edges that are not present in the structure learned by the Tabu algorithm but present in the structure learned by H2PC. Orange lines represent false negatives, i.e., edges that are present in the structure learned by the Tabu algorithm but absent in the structure learned by H2PC. All the edges from the other nodes to “Age”, “Gender” and “Born in the country” are blacklisted prior to learning the structure. In the figure, those nodes that can only be parents have a darker blue color. The node “Country” (i.e., the country of the respondent’s residency) is present in the structure but not depicted by the figure to facilitate the apprehension of the relationships between the nodes of interest. All variables are individual-level variables. (TIF) [file pone.0261663.s003.tif]

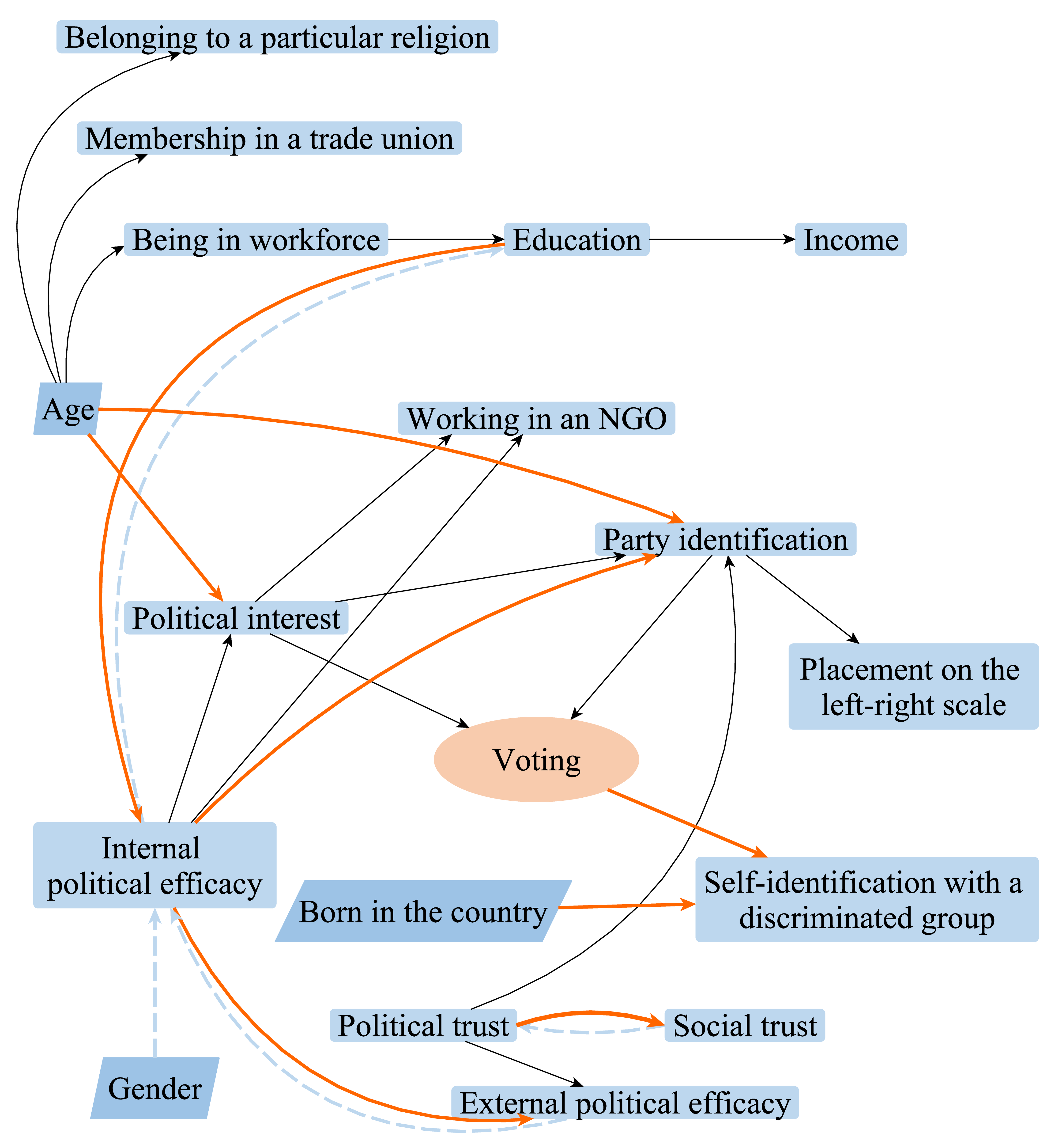

Supplement: S4 Fig — Source: [35]. N = 25 404 individuals in 19 countries. Notes: Within Bayesian network analysis, score-based Tabu and hybrid H2PC algorithms were applied to analyze the data and learn the structure of the causal relationships between the variables. Dashed blue lines represent false positives, i.e., edges that are not present in the structure learned by the Tabu algorithm but present in the structure learned by H2PC. Orange lines represent false negatives, i.e., edges that are present in the structure learned by the Tabu algorithm but absent in the structure learned by H2PC. All the edges from the other nodes to “Age”, “Gender” and “Born in the country” are blacklisted prior to learning the structure. In the figure, those nodes that can only be parents have a darker blue color. The node “Country” (i.e., the country of the respondent’s residency) is present in the structure but not depicted by the figure to facilitate the apprehension of the relationships between the nodes of interest. All variables are individual-level variables. (TIF) [file pone.0261663.s004.tif]

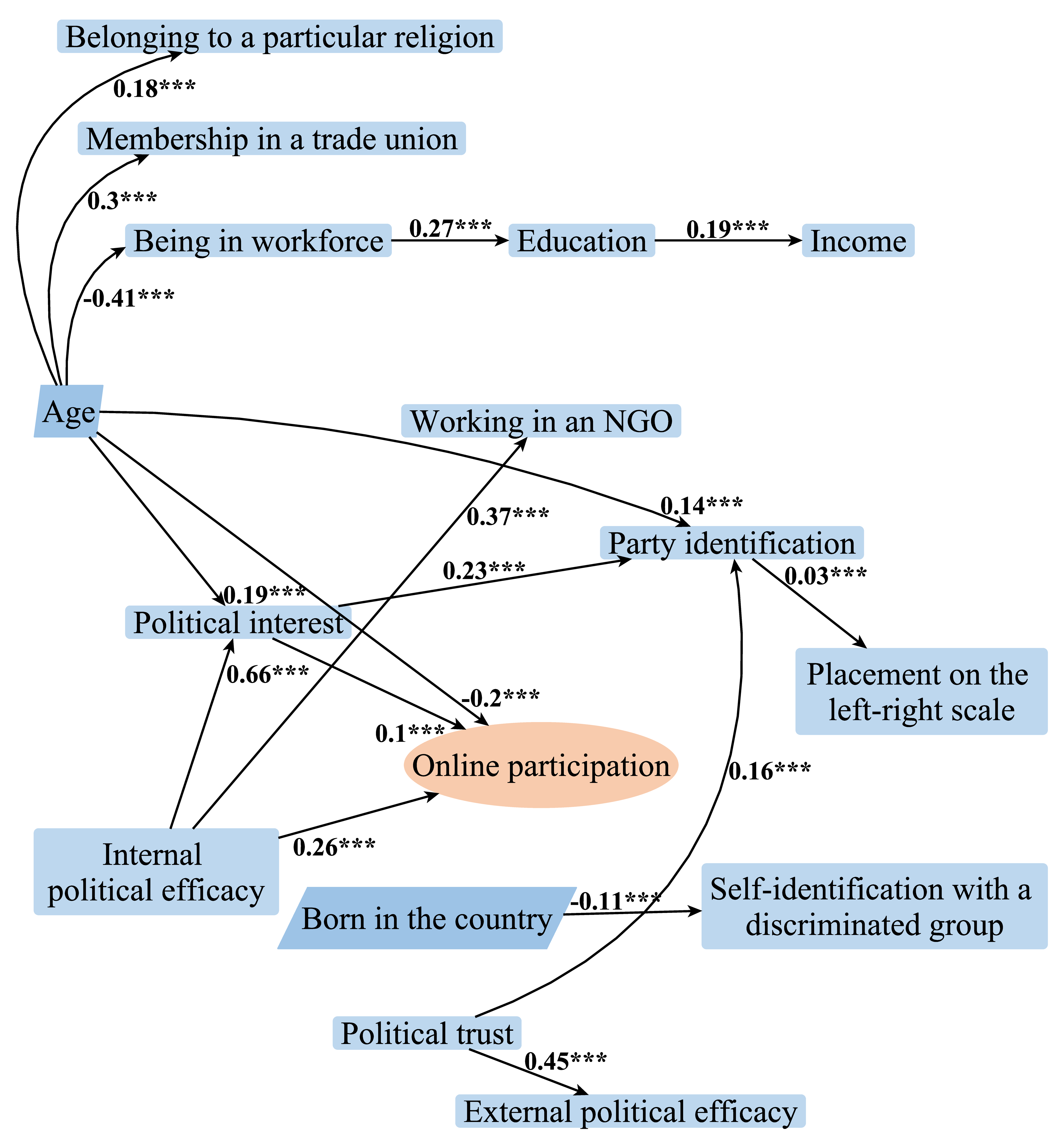

Supplement: S5 Fig — Source: [35]. N = 27 379 individuals in 19 countries. Notes: Structural equation modeling was applied to analyze the data. Only those arcs that were determined by both Tabu and H2PC algorithms are present in the model. Entities depicted in association with the edges are parameter estimates of the structural equation modeling. Sign.: *p < 0.05; **p < 0.01; ***p < 0.001. All variables are individual level variables. (TIF) [file pone.0261663.s005.tif]

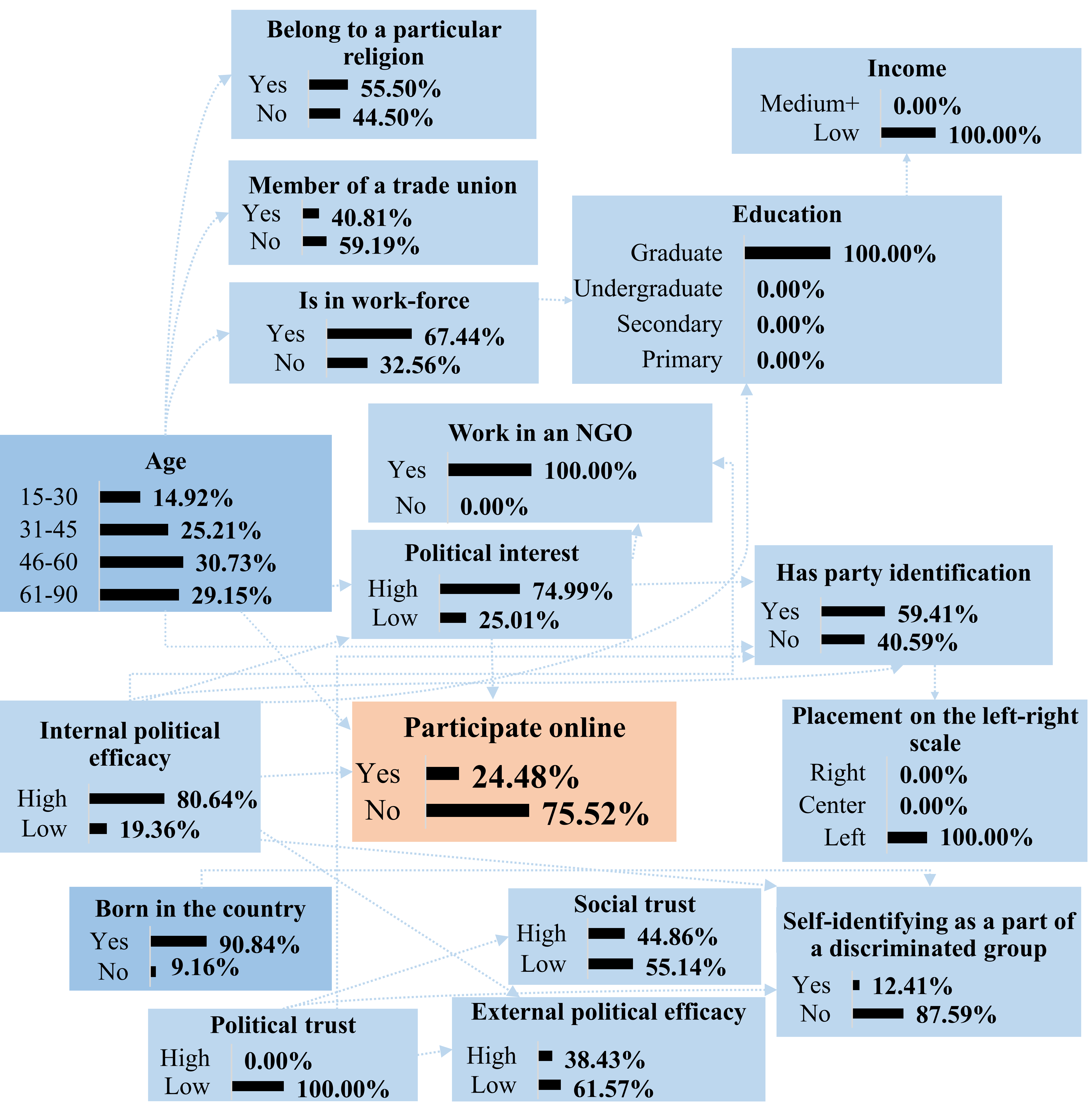

Supplement: S6 Fig — Source: ESS 2018 [35]. N = 27 379 individuals in 19 countries. Notes: Bayesian parameter estimation, conditional on the acquired structure of the network, was applied to analyse the data. Entities are the probabilities of events in percentage. The following conditional probability query was applied: education is “graduate”, placement on the left-right scale is “left”, work in an NGO is “yes”, political trust is “low” and income is “low”. (TIF) [file pone.0261663.s006.tif]

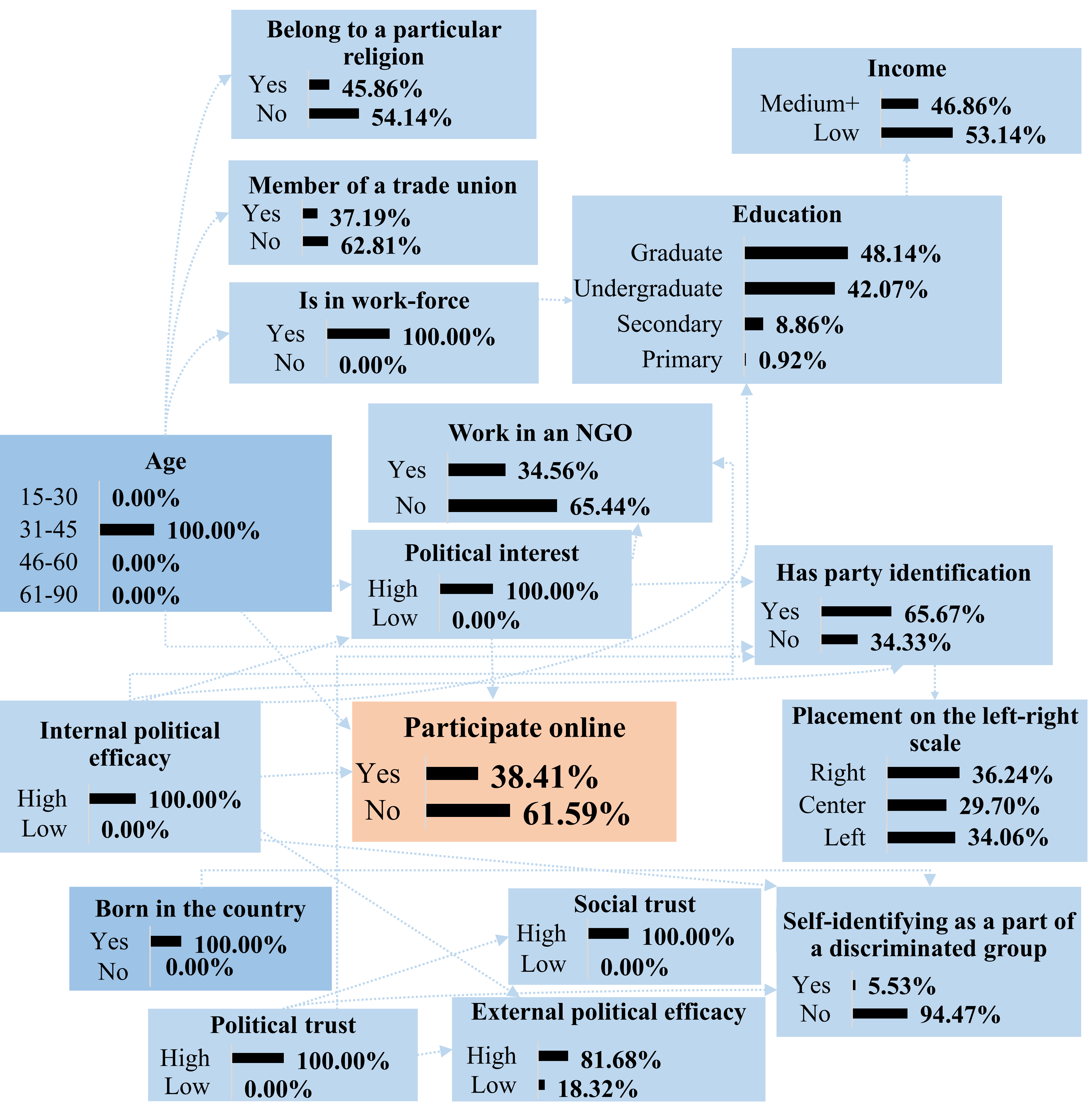

Supplement: S7 Fig — Source: [35]. N = 27 379 individuals in 19 countries. Notes: Bayesian parameter estimation, conditional on the acquired structure of the network, was applied to analyze the data. Entities are the probabilities of events in percentage. The following conditional probability query was applied: age is “31–45”, political interest is “high”, political trust is “high”, social trust is “high”, internal political efficacy is “high” and born in the country of residence is “yes”. (TIF) [file pone.0261663.s007.tif]
