# Supplementary material for: Predicting online participation through Bayesian network analysis
Source: PLoS One. 2021 Dec 23;16(12):e0261663. doi: 10.1371/journal.pone.0261663 (PMC8699968; doi:10.1371/journal.pone.0261663)
Supplement: S1 File — The document provides additional information on the data handling, methods and results and complements the main text of the manuscript. (PDF) [file pone.0261663.s008.pdf]

Supplementary information for:
Predicting online participation through Bayesian network analysis

Elizaveta Kopacheva[1*]

1 Department of Political Science & Centre for Data Intensive Sciences and Applications (DISA), Linnaeus University, Växjö, Sweden

* elizaveta.kopacheva@lnu.se

This document provides additional information on the data handling, methods and results and complements the main text of the manuscript. I have included details on: (1) the dataset, (2) data transformations and (3) results.

Table 1. Variables used for the analysis.

Variable name	Meaning	Operationalization of
pstplonl	Posted or shared anything about politics online in the last 12 months	Online activism
sgnptit	Signed a petition in the last 12 months	Signing petition
contplt	Contacted a politician or government official during the last 12 months	Contacting politicians
vote	Voted in the last national election	Participation in voting
ppltrst	Most people can be trusted or you can't be too careful	
pplfair	Most people try to take advantage of you, or try to be fair	Social trust
pplhlp	Most of the time people helpful or mostly looking out for themselves	
trstlgl	Trust in the legal system	
trstplc	Trust in the police	
trstplt	Trust in politicians	Political trust
trstprt	Trust in political parties	
trstprl	Trust in country's parliament	
polintr	How interested in politics	Political interest
psppsgva	Political system allows people to have a say in what government does	External political efficacy
psppipla	Political system allows people to have influence on politics	
clsprty	Is there a particular political party you feel closer to than all the other parties?	Party identification
lrscale	Placement on left right scale	Placement on the left-right scale
actrolga	Able to take active role in political group	Internal political efficacy
cptppola	Confident in own ability to participate in politics	
pdwrk	Doing last 7 days: paid work	
wrkorg	Worked in another organisation or association last 12 months	
rlgblg	Belonging to particular religion or denomination	Recruitment
dscrgrp	Member of a group discriminated against in this country	
mbtru	Member of trade union or similar organization	
eduyrs	Years of full-time education completed	Education
hincfel	Feeling about household's income nowadays	Income
brncntr	Were you born in country?	Nationality
gndr	Gender	Gender
agea	Age of respondent, calculated	Age
cntry	Country	Country of origin

Source: ESS 2018 [1]. N = 36 015 individuals in 19 countries.

Dataset

The dataset used for this study is the [1]. In order to examine preconditions of online activism, 30 questions of the ESS questionnaire that operationalize preconditions of political participation highlighted by [2] and some additional characteristics that are expected to influence political participation, i.e. political [3, 4, 5, 6, 7, 8, 9, 4, 10] and social trust [11, 12, 5, 13, 14], were chosen for the analysis. The list of the variables is illustrated by Table 1.

Table 2. Operationalization of social trust, political trust, external and internal political efficacy.

Operationalization of	Variables	Factor loadings	Proportion of variance explained by the factor
Social trust	ppltrst<br>pplfair<br>pplhlp	0.751<br>0.802<br>0.711	0.57
Political trust	trstlgl<br>trstplc<br>trstplt<br>trstprt<br>trstprl	0.764<br>0.646<br>0.875<br>0.847<br>0.842	0.64
External political efficacy	psppsgva<br>psppipla	0.751<br>0.751	0.56
Internal political efficacy	actrolga<br>cptppola	0.757<br>0.757	0.57

Source: ESS 2018 [1]. N = 36 015 individuals in 19 countries.

Table 3. Discretization of the variables used for the analysis.

Variable	Values	
	Before	After
Participation in online activism	2 - Did not post or share anything about politics online last 12 months<br>1 - "Posted or shared anything about politics online last 12 months"	0 - Absence<br>1 - Presence
Signing petitions	2 - Did not sign a petition last 12 months<br>1 - "Signed petition last 12 months"	0 - No<br>1 - Yes
Contacting politicians	2 - Did not contact a politician, government or local government official during the last 12 months<br>1 - "Contacted a politician, government or local government official during the last 12 months"	0 - No<br>1 - Yes
Participation in voting	2 - Did not Vote in the last election and 3 - "Not eligible to vote"<br>1 - "Voted in the last election"	0 - Absence<br>1 - Presence
Social trust	Values [-2.3693; 0)<br>Values [0; 2.0964]	0 - Low<br>1 - High
Political trust	Values [-1.9815; 0)<br>Values [0; 0.646]	0 - Low<br>1 - High
Political interest	3 - "Hardly interested" and 4 - "Not at all interested"<br>1 - "Very interested" and 2 - "Quite interested"	0 - Low interest<br>1 - High interest
External political efficacy	Values [-0.7485, 0)<br>Values [0; 1.2254]	0 - Low<br>1 - High
Internal political efficacy	Values [-0.6648; 0)<br>Values [0, 1.3811]	0 - Low<br>1 - High
Party identification	2 - Do not feel closer to a particular party<br>1 - "Feel closer to a particular party than all other parties"	0 - Absence<br>1 - Presence
Placement on left-right scale	Values in the range [0; 5)<br>Values equal to 5<br>Values in the range (5; 10]	1 - Left<br>2 - Center<br>3 - Right
Being in work-force	0 - Did not do any paid work in the last 7 days<br>1 - Doing paid work in the last 7 days	0 - Out of work-force<br>1 - In work-force
Working in a non-governmental organization	2 - Did not work in an NGO in the last 12 months<br>1 - Worked in an NGO in the last 12 months	0 - Not working<br>1 - Working
Belonging to a particular religion or denomination	2 - Not "belonging to particular religion or denomination"<br>1 - "Belonging to particular religion or denomination"	0 - Not belonging<br>1 - Belonging
Membership in a group discriminated against	2 - Not a member<br>1 - A member	0 - Not a member<br>1 - Member
Membership in a trade union	3 - Not a member<br>1 - Currently a member and 2 - Previously a member	0 - Not a member<br>1 - Member
Education	Less than 6 years of education<br>[6; 11) years of education<br>[11; 16) years of education<br>Equal to or more than 16 years of education	1 - Primary school<br>2 - Secondary school<br>3 - Undergraduate education<br>4 - Graduate education
Income	2 - "Coping on present income", 3 -"Difficult on present income" and 4 -"Very difficult on present income"<br>1-"Living comfortably on present income"	0 - Insufficient income<br>1 - Sufficient income
Born in the country	2 - Not born in country<br>1 - "Born in country"	0 - No<br>1 - Yes
Gender	2 - Female<br>1 - Male	0 - Female<br>1 - Male
Age	15-90 y.o.	4 groups: [15; 30], [31; 45], [46; 60], [61; 90]

Source: ESS 2018 [1]. N = 36 015 individuals in 19 countries.

Data transformations

Exploratory factor analysis (using Ordinary Least Squares to obtain the minimum residual solution [15]) was applied to 12 out of 30 ESS questions to reduce the number of variables and operationalize social trust, political trust, external and internal political efficacy.

The analysis was performed using the *R 4.0.1 platform* [16] and the additional package *psych* [15]. The factor loadings are presented by Table 2.

In order to perform Bayesian structure learning, all the variables were discretized as suggested by [17]. The variables were discretized as illustrated by Table 3. Table 4 shows all data transformations.

Table 4. Data transformations.

Original		Recoded		After dimensionality reduction		Discretised	
**Participation in online activism (pstplonl)**							
1	5362 (14.9%)	0	30445 (84.5%)		-		-
2	30445 (84.5%)	1	5362 (14.9%)		-		-
Missing	208 (0.6%)		-		-		-
**Signing petitions (sgnptit)**							
1	8608 (23.9%)	0	27171 (75.4%)		-		-
2	27171 (75.4%)	1	8608 (23.9%)		-		-
Missing	236 (0.7%)		-		-		-
**Contacting politicians (contplt)**							
1	5445 (15.1%)	0	30395 (84.4%)		-		-
2	30395 (84.4%)	1	5445 (15.1%)		-		-
Missing	175 (0.5%)		-		-		-
**Voting (vote)**							
1	25447 (70.7%)	0	10156 (28.2%)		-		-
2	7208 (20.0%)	1	25447 (70.7%)		-		-
3	2948 (8.2%)				-		-
Missing	412 (1.1%)		-		-		-
**Most people can be trusted (ppltrst)**							
Mean	5.09		-	Mean	0.0021	0	16443 (45.7%)
(SD)	(2.45)		-	(SD)	(0.894)	1	19212 (53.3%)
Median	5.00		-	Median	0.103		
[Min, Max]	[0, 10.0]		-	[Min, Max]	[-2.37, 2.10]		
Missing	109 (0.3%)		-	Missing	360 (1.0%)		-
**Most people try to take advantage of you (pplfair)**							
Mean	5.63		-		-		-
(SD)	(2.30)		-		-		-
Median	6.00		-		-		-
[Min, Max]	[0, 10.0]		-		-		-
Missing	228 (0.6%)		-		-		-
**Most of the time people helpful (pplhlp)**							
Mean	5.03		-		-		-
(SD)	(2.33)		-		-		-
Median	5.00		-		-		-
[Min, Max]	[0, 10.0]		-		-		-
Missing	140 (0.4%)		-		-		-
**Trust in the legal system (trstlgl)**							
Mean	5.49		-	Mean	0.0001	0	15992 (44.4%)
(SD)	(2.67)		-	(SD)	(0.950)	1	18392 (51.1%)
Median	6.00		-	Median	0.0925		
[Min, Max]	[0, 10.0]		-	[Min, Max]	[-1.98, 2.40]		
Missing	702 (1.9%)		-	Missing	1631 (4.5%)		-
**Trust in the police (trstplc)**							
Mean	6.46		-		-		-
(SD)	(2.48)		-		-		-
Median	7.00		-		-		-
[Min, Max]	[0, 10.0]		-		-		-
Missing	268 (0.7%)		-		-		-
**Trust in politicians (trstplt)**							
Mean	3.78		-		-		-
(SD)	(2.45)		-		-		-
Median	4.00		-		-		-
[Min, Max]	[0, 10.0]		-		-		-

Missing	723 (2.0%)		-		-		-
**Trust in political parties (trstprt)**							
Mean	3.72		-		-		-
(SD)	(2.41)		-		-		-
Median	4.00		-		-		-
[Min, Max]	[0, 10.0]		-		-		-
Missing	831 (2.3%)		-		-		-
**Trust in country's parliament (trstprl)**							
Mean	4.64		-		-		-
(SD)	(2.61)		-		-		-
Median	5.00		-		-		-
[Min, Max]	[0, 10.0]		-		-		-
Missing	868 (2.4%)		-		-		-
**Political interest (polintr)**							
1	3901 (10.8%)	0	20541 (57.0%)		-		-
2	11499 (31.9%)	1	15400 (42.8%)		-		-
3	13152 (36.5%)						
4	7389 (20.5%)						
Missing	74 (0.2%)		-		-		-
**Political system allows people to have a say in what government does (psppsgva)**							
Mean	2.26		-	Mean	0.0015	0	17878 (49.6%)
(SD)	(0.960)		-	(SD)	(0.848)	1	16644 (46.2%)
Median	2.00		-	Median	-0.749		
[Min, Max]	[1.00, 5.00]		-	[Min, Max]	[-0.749, 1.23]		
Missing	1169 (3.2%)		-	Missing	1493 (4.1%)		-
**Political system allows people to have influence on politics (psppipla)**							
Mean	2.19		-		-		-
(SD)	(0.940)		-		-		-
Median	2.00		-		-		-
[Min, Max]	[1.00, 5.00]		-		-		-
Missing	975 (2.7%)		-		-		-
**Party identification (clsprty)**							
1	16401 (45.5%)	0	18596 (51.6%)		-		-
2	18596 (51.6%)	1	16401 (45.5%)		-		-
Missing	1018 (2.8%)		-		-		-
**Placement on the left-right scale (lrscale)**							
Mean	5.09		-		-	1	9738 (27.0%)
(SD)	(2.20)		-		-	2	10303 (28.6%)
Median	5.00		-		-	3	10809 (30.0%)
[Min, Max]	[0, 10.0]		-		-		
Missing	5165 (14.3%)		-		-		-
**Able to take active role in political group (actrolga)**							
Mean	2.07		-	Mean	0.0038	0	19973 (55.5%)
(SD)	(1.06)		-	(SD)	(0.853)	1	14438 (40.1%)
Median	2.00		-	Median	-0.665		
[Min, Max]	[1.00, 5.00]		-	[Min, Max]	[-0.665, 1.38]		
Missing	935 (2.6%)		-	Missing	1604 (4.5%)		-
**Confident in own ability to participate in politics (cptppola)**							
Mean	2.14		-		-		-
(SD)	(1.07)		-		-		-
Median	2.00		-		-		-
[Min, Max]	[1.00, 5.00]		-		-		-
Missing	1217 (3.4%)		-		-		-
**Doing paid work (pdwrk)**							
0	17016 (47.2%)		-		-		-
1	18999 (52.8%)		-		-		-
**Worked in another organisation or association (wrkorg)**							
1	5405 (15.0%)	0	30432 (84.5%)		-		-
2	30432 (84.5%)	1	5405 (15.0%)		-		-
Missing	178 (0.5%)		-		-		-
**Belonging to particular religion or denomination (rlgblg)**							
1	20712 (57.5%)	0	15023 (41.7%)		-		-
2	15023 (41.7%)	1	20712 (57.5%)		-		-
Missing	280 (0.8%)		-		-		-
**Member of a group discriminated against in this country (dscrgrp)**							
1	2611 (7.2%)	0	33083 (91.9%)		-		-
2	33083 (91.9%)	1	2611 (7.2%)		-		-
Missing	321 (0.9%)		-		-		-
**Member of trade union or similar organisation (mbtru)**							
1	4995 (13.9%)	0	22954 (63.7%)		-		-

2	7829 (21.7%)	1	12824 (35.6%)		-		-
3	22954 (63.7%)						
Missing	237 (0.7%)		-		-		-
**Education (eduyrs)**							
Mean	13.0	Mean	0.130		-	1	1073 (3.0%)
(SD)	(4.00)	(SD)	(0.0400)		-	2	6952 (19.3%)
Median	12.0	Median	0.120		-	3	18233 (50.6%)
[Min, Max]	[0, 51.0]	[Min, Max]	[0, 0.510]			4	9252 (25.7%)
Missing	505 (1.4%)		-		-		-
**Income (hincfel)**							
1	10964 (30.4%)	0	24501 (68.0%)		-		-
2	16485 (45.8%)	1	10964 (30.4%)		-		-
3	5856 (16.3%)						
4	2160 (6.0%)						
Missing	550 (1.5%)		-		-		-
**Nationality (brncntr)**							
1	32333 (89.8%)	0	3657 (10.2%)		-		-
2	3657 (10.2%)	1	32333 (89.8%)		-		-
Missing	25 (0.1%)		-		-		-
**Gender (gndr)**							
1	16982 (47.2%)	0	19033 (52.8%)		-		-
2	19033 (52.8%)	1	16982 (47.2%)		-		-
**Age (agea)**							
Mean	50.7	Mean	0.507		-	1	6334 (17.6%)
(SD)	(18.7)	(SD)	(0.187)		-	2	8014 (22.3%)
Median	51.0	Median	0.510		-	3	9317 (25.9%)
[Min, Max]	[15.0, 90.0]	[Min, Max]	[0.150, 0.900]		-	4	12183 (33.8%)
Missing	167 (0.5%)		-		-		-

Source: ESS 2018 [1]. N = 36 015 individuals in 19 countries. Refer to Table 3 to decode the original value codes.

Results

Structure learning

The Bayesian network structure learning, parameter learning and inference were performed using the *R 4.0.1 platform* [16] and the additional packages *bnlearn* [18] and *gRain* [19].

In order to perform Bayesian network structure learning, constraint-based, score-based and hybrid algorithms within the package *bnlearn* were applied and scores of the received networks were compared. The difference between the constraint-based, score-based and hybrid algorithms is the following.

- Constraint-based algorithms apply statistical tests to identify conditional independence constraints of linking two network nodes.
- Score-based algorithms apply general optimisation techniques to increase the value of the network score, which shows the goodness-of-fit of the model [17].
- Hybrid algorithms utilize the tools of constraint-based techniques to reduce the number of possible directed acyclic graphs and tools of score-based algorithms to choose the network with the highest score [18].

To receive a better predictive performance, model averaging was performed [17, p. 140]. Fig 1 shows the models that were learned on the sets of 5000 network structures applying Tabu and H2PC algorithms.

Fig 1. Directed acyclic graphs (DAG) of the relationships between factors associated with participation in online activism. *Source:* [1]. N=27 379 individuals in 19 countries. *Notes:* Within Bayesian network analysis, score-based Tabu and hybrid H2PC algorithms were applied to analyze the data and learn the structure of the causal relationships between the variables. Dashed blue lines represent false positives, i.e., edges that are not present in the structure learned by the Tabu algorithm but present in the structure learned by H2PC. Orange lines represent false negatives, i.e., edges that are present in the structure learned by the Tabu algorithm but absent in the structure learned by H2PC. All the edges from the other nodes to "Age", "Gender" and "Born in the country" are blacklisted prior to learning the structure. In the figure, those nodes that can only be parents have a darker blue color. The node "Country" (i.e., the country of the respondent's residency) is present in the structure but not depicted by the figure to facilitate the apprehension of the relationships between the nodes of interest. All variables are individual-level variables.

As a robustness test, the relationships between predictor variables were investigated by applying the same Tabu and H2PC algorithms to ESS data but using participation in petition signing, as another example of a non-institutionalised form of political participation [10], as an outcome variable.

Fig 2 shows the models that were learned on the sets of 5000 network structures by applying Tabu and H2PC algorithms to the ESS data using participation in petition signing as an outcome variable.

Fig 2. Directed acyclic graphs of the relationships between factors associated with participation in petition signing. *Source:* [1]. N=27 366 individuals in 19 countries. *Notes:* Within Bayesian network analysis, score-based Tabu and hybrid H2PC algorithms were applied to analyze the data and learn the structure of the causal relationships between the variables. Dashed blue lines represent false positives, i.e., edges that are not present in the structure learned by the Tabu algorithm but present in the structure learned by H2PC. Orange lines represent false negatives, i.e., edges that are present in the structure learned by the Tabu algorithm but absent in the structure learned by H2PC. All the edges from the other nodes to "Age", "Gender" and "Born in the country" are blacklisted prior to learning the structure. In the figure, those nodes that can only be parents have a darker blue color. The node "Country" (i.e., the country of the respondent's residency) is present in the structure but not depicted by the figure to facilitate the apprehension of the relationships between the nodes of interest. All variables are individual-level variables.

To check the robustness, the algorithms were also applied to analyse the relationships between all the mentioned variables including participation in online activism and petition signing. Once again, the models were learned on the sets of 5000 network structures. Fig 3 shows those models.

Fig 3. Directed acyclic graphs of the relationships between factors associated with participation in online activism and petition signing. *Source:* [1]. N=27 323 individuals in 19 countries. *Notes:* Within Bayesian network analysis, score-based Tabu and hybrid H2PC algorithms were applied to analyze the data and learn the structure of the causal relationships between the variables. Dashed blue lines represent false positives, i.e., edges that are not present in the structure learned by the Tabu algorithm but present in the structure learned by H2PC. Orange lines represent false negatives, i.e., edges that are present in the structure learned by the Tabu algorithm but absent in the structure learned by H2PC. All the edges from the other nodes to "Age", "Gender" and "Born in the country" are blacklisted prior to learning the structure. In the figure, those nodes that can only be parents have a darker blue color. The node "Country" (i.e., the country of the respondent's residency) is present in the structure but not depicted by the figure to facilitate the apprehension of the relationships between the nodes of interest. All variables are individual-level variables.

Additional tests, which aimed to check for robustness causal relations between variables associated with participation, included learning structures of the networks that consisted of the same variables but conventional forms of participation, i.e., contacting a politician and voting, instead of participation in online activism and petition signing. The causality structure learned in relation to participation in contacting politicians is presented in Fig 4 while Fig 5 shows the directed acyclic graphs of the relationships between factors associated with participation in voting.

Fig 4. Directed acyclic graphs of the relationships between factors associated with participation in contacting politicians. *Source:* [1]. N=27 397 individuals in 19 countries. *Notes:* Within Bayesian network analysis, score-based Tabu and hybrid H2PC algorithms were applied to analyze the data and learn the structure of the causal relationships between the variables. Dashed blue lines represent false positives, i.e., edges that are not present in the structure learned by the Tabu algorithm but present in the structure learned by H2PC. Orange lines represent false negatives, i.e., edges that are present in the structure learned by the Tabu algorithm but absent in the structure learned by H2PC. All the edges from the other nodes to "Age", "Gender" and "Born in the country" are blacklisted prior to learning the structure. In the figure, those nodes that can only be parents have a darker blue color. The node "Country" (i.e., the country of the respondent's residency) is present in the structure but not depicted by the figure to facilitate the apprehension of the relationships between the nodes of interest. All variables are individual-level variables.

Here, we can see a similar structure of the causal relations between the variables associated with participation. Only participation in voting has interesting trends in terms of the causality between the factors relevant to political participation. Fig 5 shows that internal political efficacy does not have a direct effect on participation in voting as well as the relation between working in an NGO and voting is not direct suggesting that recruitment via NGOs is not significant in regard to participation in voting. In the meanwhile, party identification has a direct effect on voting that could be expected and in line with the suggestions of [2].

Fig 5. Directed acyclic graphs of the relationships between factors associated with participation in voting. *Source:* [1]. N=25 404 individuals in 19 countries. *Notes:* Within Bayesian network analysis, score-based Tabu and hybrid H2PC algorithms were applied to analyze the data and learn the structure of the causal relationships between the variables. Dashed blue lines represent false positives, i.e., edges that are not present in the structure learned by the Tabu algorithm but present in the structure learned by H2PC. Orange lines represent false negatives, i.e., edges that are present in the structure learned by the Tabu algorithm but absent in the structure learned by H2PC. All the edges from the other nodes to "Age", "Gender" and "Born in the country" are blacklisted prior to learning the structure. In the figure, those nodes that can only be parents have a darker blue color. The node "Country" (i.e., the country of the respondent's residency) is present in the structure but not depicted by the figure to facilitate the apprehension of the relationships between the nodes of interest. All variables are individual-level variables.

Comparing all of the structures, several uncertainties in relation to the relationships between the variables were distinguished.

Thus, the following causal relations are questioned.

1. Direct or reverse causation between political and social trust.
2. Direct or reverse causation between education and internal political efficacy.
3. Direct or reverse causation between external and internal political efficacy.
4. Direct causation between political interest and working in an NGO.
5. Direct or reverse causation between participation in online activism and working in an NGO.
6. Direct causation between gender and internal political efficacy.
7. Direct causation between participation in online activism and party identification.
8. Direct causation between internal political efficacy and party identification.
9. Direct causation between political trust and self-identification with a discriminated group.
10. Direct causation between internal political efficacy and self-identification with a discriminated group.
11. Direct causation between participation in online activism and self-identification with a discriminated group.

In order to test for the significance of the mentioned paths, structural equation modeling was conducted.

Testing the learned paths' significance

In order to test if the questioned arcs are improving the structure of the network, structural equation modeling was conducted in several steps. The first step was to test the significance of the model that included only those arcs that were determined by both of the algorithms, i.e., Tabu and H2PC, and was also found by the same algorithms when conducting the robustness tests.

Structural equation modeling were performed using the *R 4.0.1 platform* [16] and the additional package *lavaan* [22].

Fig 6 shows the results of such estimations.

Fig 6. Directed acyclic graph of the relationships between factors associated with participation in online activism. *Source:* [1]. N=27 379 individuals in 19 countries. *Notes:* Structural equation modeling was applied to analyze the data. Only those arcs that were determined by both Tabu and H2PC algorithms are present in the model. Entities depicted in association with the edges are parameter estimates of the structural equation modeling. Sign.: $^{*}p < 0.05$; $^{**}p < 0.01$; $^{***}p < 0.001$. All variables are individual level variables.

After estimating the fitness of the initial model, other arcs were added one by one. The structures were compared applying chi-square tests and proceeding with the best-model-fit structures. As a result of the comparison, the following structure was received (see Fig 7).

Fig 7. Directed acyclic graph of the relationships between factors associated with participation in online activism. *Source:* [1]. N=27 379 individuals in 19 countries. *Notes:* Structural equation modeling was applied to analyze the data. Structure with the best model fit is presented. Entities depicted in association with the edges are parameter estimates of the structural equation modeling. Sign.: $^{*}p < 0.05$; $^{**}p < 0.01$; $^{***}p < 0.001$. All variables are individual level variables.

The structure illustrated in Fig 7 is the Directed acyclic graph (DAG) that was used to fit the parameters of the Bayesian network and acquire the set of conditional probability distribution tables.

Exacting inference

Once, the structure with the best model fit was determined, a new empty graph was created using *bnlearn* tools. The list of arcs was set to those directed edges that were determined in the previous step. Thus, a network consisting of 36 directed edges was transformed into a junction tree and its probability tables were computed.

Fig 8 visualizes the junction tree.

Fig 8. Probability distribution of all factors associated with participation in online activism. *Source:* [1]. N=27 379 individuals in 19 countries. *Notes:* Bayesian parameter estimation, conditional on the acquired structure of the network, was applied to analyze the data. Entities are the probabilities of events in percentage.

Using the received probability distribution tables, it became possible to construct conditional probability queries, i.e., calculating the probabilities corresponding to an event under specific conditions [17, p. 20].

One of the examples of such query may be calculating the probability of a person to participate in online activism if this person is an individual with a low income, graduate level of education, working in a non-governmental organization, placing oneself on the left of the left-right scale and having a low political trust.

Setting the evidence in the described way will change local distributions of the nodes as presented by Fig 9.

Fig 9. Probability distribution of all factors associated with participation in online activism. *Source:* [1]. N=27 379 individuals in 19 countries. *Notes:* Bayesian parameter estimation, conditional on the acquired structure of the network, was applied to analyze the data. Entities are the probabilities of events in percentage. The following conditional probability query was applied: education is "graduate", placement on the left-right scale is "left", work in an NGO is "yes", political trust is "low" and income is "low".

Fig 9 shows how the probability of a person to participate in online activism increased from 17,03% to 24,48% under the specified conditions.

The following figure shows how the probability would change if we wanted to predict the political participation of a 31-45 y.o. employed person who was born in the country of residence and has high levels of political trust, internal political efficacy, political interest and social trust.

Fig 10. Probability distribution of all factors associated with participation in online activism. *Source:* [1]. N=27 379 individuals in 19 countries. *Notes:* Bayesian parameter estimation, conditional on the acquired structure of the network, was applied to analyze the data. Entities are the probabilities of events in percentage. The following conditional probability query was applied: age is "31-45", political interest is "high", political trust is "high", social trust is "high", internal political efficacy is "high" and born in the country of residence is "yes".

Fig 10 shows a significant increase in the probability of a person to participate in online activism due to the fact that the factors directly affecting participation, i.e., political interest, internal political efficacy and age, are known.

References

1. ESS round 9: European social survey round 9 data.Data file edition 1.2.; 2018. NSD - Norwegian Centre for Research Data, Norway – Data Archive and distributor of ESS data for ESS ERIC.
2. Verba S, Schlozman KL, Brady HE. Voice and equality: civic voluntarism in American politics. Cambridge, MA: Harvard University Press; 1995.
3. Inglehart R. Modernization and postmodernization: Cultural, economic, and political change in 43 societies. Princeton, NJ: Princeton University Press; 1997.
4. Norris P. Critical citizens global support for democratic government. Oxford: Oxford University Press; 1999.
5. Norris P. Democratic phoenix: Reinventing political activism. Cambridge: Cambridge University Press; 2002.
6. Pollock PH. The participatory consequences of internal and external political efficacy: A research note. The Western Political Quarterly. 1983;36(3):400–409. doi:https://doi.org/10.2307/448398.
7. Inglehart R, Welzel C. Modernization, cultural change, and democracy: The human development sequence. New York; Cambridge: Cambridge University Press; 2005.
8. Nye JS, Zelikow P, King DC. Why people don't trust government. Cambridge, MA: Harvard University Press; 1997.

9. Miller AH. Political issues and trust in government: 1964–1970. American political science review. 1974;68(3):951–972. doi:https://doi.org/10.2307/1959140.

10. Marien S, Hooghe M, Quintelier E. Inequalities in non-institutionalised forms of political participation: A multi-level analysis of 25 countries. Political Studies. 2010;58:187–213. doi:10.1111/j.1467-9248.2009.00801.x.

11. Putnam RD. Making democracy work: Civic traditions in modern Italy. Princeton, NJ: Princeton University Press; 1993.

12. Putnam RD. Bowling alone: The collapse and revival of American community. New York: Simon and Schuster; 2000.

13. Benson M, Rochon TR. Interpersonal trust and the magnitude of protest: A micro and macro level approach. Comparative Political Studies. 2004;37(4):435–457. doi:10.1177/0010414003262900.

14. Kaase M. Interpersonal trust, political trust and non-institutionalised political participation in Western Europe. West European Politics. 1999;22(3):1–21. doi:10.1080/01402389908425313.

15. Revelle W. Package 'psych': Procedures for psychological, psychometric, and personality research; 2019. The Comprehensive R Archive Network.

16. R Core Team. R: A language and environment for statistical computing; 2020. Available from: `https://www.R-project.org/`.

17. Scutari M, Denis JB. Bayesian networks: with examples in R. Boca Raton, FL: CRC press; 2014.

18. Scutari M, Ness R. Package 'bnlearn': Bayesian network structure learning, parameter learning and inference; 2019. The Comprehensive R Archive Network.

19. Højsgaard S. Package 'gRain': Graphical independence networks; 2020. The Comprehensive R Archive Network.

20. Glover F. Tabu search — part I. ORSA Journal on computing. 1989;1(3):190–206. doi:https://doi.org/10.1287/ijoc.1.3.190.

21. Gasse M, Aussem A, Elghazel H. A hybrid algorithm for Bayesian network structure learning with application to multi-label learning. Expert Systems with Applications. 2014;41(15):6755–6772. doi:https://doi.org/10.1016/j.eswa.2014.04.032.

22. Rosseel Y, Oberski D, Byrnes J, Vanbrabant L, Savalei V, Merkle E, et al.. Package 'lavaan': Latent variable analysis; 2020. The Comprehensive R Archive Network.
