# Supplementary material for: Predicting online participation through Bayesian network analysis
Source: PLoS One. 2021 Dec 23;16(12):e0261663. doi: 10.1371/journal.pone.0261663 (PMC8699968; doi:10.1371/journal.pone.0261663)
Supplement: S2 File — The R script used for the analysis. (PDF) [file pone.0261663.s009.pdf]

Supplementary material for: Predicting online participation through Bayesian network analysis. R script

8/14/2020

Loading the data

The following code will load the dataset. It is an SPSS file, thus, foreign package (R Core Team 2020a) is used to load the data.

```
#install.packages("foreign",dependencies = TRUE)
library(foreign)

# loading the file
ESS9Dat<-foreign::read.spss("ESS9e01_1.sav",
                            use.value.labels=FALSE,
                            to.data.frame=T)


# Selecting the needed variables
ESS9Dat2<-subset(ESS9Dat,
                 select=c("cntry",#Country
                          "lrscale",#Placement on left right scale
                          "ppltrst",#Most people can be trusted or you
                                    #can't be too careful
                          "pplfair",#Most people try to take advantage
                                    #of you, or try to be fair
                          "pplhlp",#Most of the time people helpful or
                                   #mostly looking out for themselves
                          "trstlgl",#Trust in the legal system
                          "trstplc",#Trust in the police
                          "trstplt",#Trust in politicians
                          "trstprt",#Trust in political parties
                          "trstprl",#Trust in country's parliament
                          "clsprty",#Is there a particular political
                                    #party you feel closer to than all
                                    #the other parties?
                          "polintr",#How interested in politics
                          "psppsgva",#Political system allows people
                                     #to have a say in what government
                                     #does
                          "psppipla",#Political system allows people to
                                     #have influence on politics
                          "actrolga",#Able to take active role in
                                     #political group
                          "cptppola",#Confident in own ability to
                                     #participate in politics
```

```
                          "sgnptit",#Signed a petition in the last 12
                                    #months
                          "pstplonl",#Posted or shared anything about
                                     #politics online in the last 12
                                     #months
                          "wrkorg",#Worked in another organisation or
                                   #association last 12 months
                          "rlgblg",#Belonging to particular religion
                                   #or denomination
                          "dscrgrp",#Member of a group discriminated
                                    #against in  this country
                          "mbtru",#Member of trade union or similar
                                  #organization
                          "gndr",#Gender
                          "agea",#Age
                          "eduyrs",#Years of full-time education completed
                          "pdwrk",#Doing last 7 days: paid work
                          "brncntr",#Were you born in country
                          "hincfel",#Feeling about household's income
                                    #nowadays
                          "dweight"))#Weight
```

Transforming the data

```
# Variable polintr (political interest)
# 1 and 2, "very interested" and "quite interested", are 1 = "interested"
# 3 and 4, "hardly interested" and "not at all interested", are
# 0 = "not interested"
ESS9Dat2$polintr<-ifelse(ESS9Dat2$polintr==1|ESS9Dat2$polintr==2,1,0)

# Variable psppsgva (political system allows people to have a say in
# what government does)
# 1 and 2, "Not at all" and "Very little", are 0 = "Political system
# does not allow people to have a say in what government does"
# 3, 4, and 5, "Some","A lot" and "Great deal", are 1 = "Political
# system allows people to have a say in what government does"
ESS9Dat2$psppsgva<-ifelse(ESS9Dat2$psppsgva==1|ESS9Dat2$psppsgva==2,0,1)

# Variable psppila (political system allows people to have influence
# on politics)
# 1 and 2, "Not at all" and "Very little", are 0 = "Political system
# does not allow people to have influence on politics"
# 3, 4, and 5, "Some","A lot" and "Great deal", are 1 = "Political
# system allows people to have influence on politics"
ESS9Dat2$psppipla<-ifelse(ESS9Dat2$psppipla==1|ESS9Dat2$psppipla==2,0,1)

# Variable clsprty (Is there a particular political party you feel
# closer to than all the other parties?)
# 1, "yes", is 1 = "Feel closer to a particular party than all other
# parties"
# 2, "no", is 0 = "No"
ESS9Dat2$clsprty<-ifelse(ESS9Dat2$clsprty==1,1,0)
```

```
# Variable actrolga (Able to take active role in political group)
# 1 and 2, "Not at all" and "Very little", are 0 = "Not able to take
# active role in political group"
# 3, 4, and 5, "Some","A lot" and "Great deal", are 1 = "Able to take
# active role in political group"
ESS9Dat2$actrolga<-ifelse(ESS9Dat2$actrolga==1|ESS9Dat2$actrolga==2,0,1)

# Variable cptppola (Confident in own ability to participate in politics)
# 1 and 2, "Not at all" and "Very little", are 0 = "Not confident in
# own ability to participate in politics"
# 3, 4, and 5, "Some","A lot" and "Great deal", are 1 = "Confident in
# own ability to participate in politics"
ESS9Dat2$cptppola<-ifelse(ESS9Dat2$cptppola==1|ESS9Dat2$cptppola==2,0,1)

# Variable sgnptit (signed a petition)
# 1, "yes", is 1 = "Signed petition last 12 months"
# 2, "no", is 0 = "No"
ESS9Dat2$sgnptit<-ifelse(ESS9Dat2$sgnptit==1,1,0)

# Variable pstplonl (posted political information online)
# 1, "yes", is 1 = "Posted or shared anything about politics online
# last 12 months"
# 2, "no", is 0 = "No"
ESS9Dat2$pstplonl<-ifelse(ESS9Dat2$pstplonl==1,1,0)

# Variable wrkorg (worked in another organization)
# 1, "yes", is 1 = "Worked in another organization or association
# last 12 months"
# 2, "no", is 0 = "No"
ESS9Dat2$wrkorg<-ifelse(ESS9Dat2$wrkorg==1,1,0)

# Variable rlgblg (belong to religion)
# 1, "yes", is 1 = "Belonging to particular religion or denomination"
# 2, "no", is 0 = "No"
ESS9Dat2$rlgblg<-ifelse(ESS9Dat2$rlgblg==1,1,0)

# Variable dscrgrp (Member of a group discriminated against in this
# country)
# 1, "yes", is 1 = "Member of a group discriminated against in this
# country"
# 2, "no", is 0 = "No"
ESS9Dat2$dscrgrp<-ifelse(ESS9Dat2$dscrgrp==1,1,0)

# Variable mbtru (member of a trade union)
# 1 and 2, "yes currently" and "yes previously", are 1 = "Member of
# trade union or similar organisation"
# 3, "no", is 0 = "No"
ESS9Dat2$mbtru<-ifelse(ESS9Dat$mbtru==3,0,1)

# Variable brncntr (born in country)
# 1, "yes", is 1 = "Born in country"
# 2, "no", is 0 = "No"
ESS9Dat2$brncntr<-ifelse(ESS9Dat2$brncntr==1,1,0)
```

```
# Variable gndr (gender)
# 1, "male", is 1 = "male"
# 2, "female", is 0 = "female"
ESS9Dat2$gndr<-ifelse(ESS9Dat2$gndr==1,1,0)

# Variable hincfel (income)
# 1-"Living comfortably on present income" is 1-"Living comfortably on
# present income" ;
# 2-"Coping on present income", 3-"Difficult on present income" and
# 4-"Very difficult on present income" are 0-"Not living comfortably
# on present income"
ESS9Dat2$hincfel<-ifelse(ESS9Dat2$hincfel==1,1,0)

# transforming variables for the age (agea) and years of education
#(eduyrs)
ESS9Dat2$agea100 <- ESS9Dat2$agea / 100
ESS9Dat2$eduyrs100 <- ESS9Dat2$eduyrs / 100
#now age and years of education are counted in hundreds of years
```

Applying factor analysis

Factor analysis must be applied to some of the variables to decrease dimensionality. Here, two packages are used. Those are psych package (Revelle 2020) to apply factor analysis and stats package (2020b) to see the factor loadings.

```
#install.packages(c("psych","stats"),dependencies = TRUE)
library(psych) # to apply factor analysis
library(stats) # to see the loadings
```

```
# Creating a new variable "political trust"
pol.trust2<-fa(as.matrix(ESS9Dat2[,c("trstplt",#Trust in politicians
                                     "trstprt",#Trust in political
                                               #parties
                                      "trstprl",#Trust in country's
                                                #parliament
                                      "trstlgl",#Trust in the legal system
                                      "trstplc")]),#Trust in the police
               nfactors=1, #to receive one variable "political trust"
               weight=ESS9Dat2$dweight, #variables are weighted
               residuals=TRUE)

#Looking at the summary
options(width = 50)
summary(pol.trust2)
```

```
##
## Factor analysis with Call: fa(r = as.matrix(ESS9Dat2[, c("trstplt", "
   trstprt", "trstprl",
##     "trstlgl", "trstplc")]), nfactors = 1, residuals = TRUE,
##     weight = ESS9Dat2$dweight)
##
```

```
## Test of the hypothesis that 1 factor is sufficient.
## The degrees of freedom for the model is 5  and the objective function
   was  0.51
## The number of observations was  36015  with Chi Square =  18365.55
   with prob <  0
##
## The root mean square of the residuals (RMSA) is  0.09
## The df corrected root mean square of the residuals is  0.12
##
## Tucker Lewis Index of factoring reliability =  0.708
## RMSEA index =  0.319  and the 10 % confidence intervals are  0.315
   0.323
## BIC =  18313.09
```

```
#Examining the loadings
loadings(pol.trust2)
```

```
##
## Loadings:
##         MR1
## trstplt 0.875
## trstprt 0.847
## trstprl 0.842
## trstlgl 0.764
## trstplc 0.646
##
##                  MR1
## SS loadings    3.193
## Proportion Var 0.639
```

```
print.psych(pol.trust2,cut=0.3,sort=TRUE)
```

```
## Factor Analysis using method =  minres
## Call: fa(r = as.matrix(ESS9Dat2[, c("trstplt", "trstprt", "trstprl",
##     "trstlgl", "trstplc")]), nfactors = 1, residuals = TRUE,
##     weight = ESS9Dat2$dweight)
## Standardized loadings (pattern matrix) based upon correlation matrix
##         V  MR1   h2   u2 com
## trstplt 1 0.88 0.77 0.23   1
## trstprt 2 0.85 0.72 0.28   1
## trstprl 3 0.84 0.71 0.29   1
## trstlgl 4 0.76 0.58 0.42   1
## trstplc 5 0.65 0.42 0.58   1
##
##                 MR1
## SS loadings    3.19
## Proportion Var 0.64
##
## Mean item complexity =  1
## Test of the hypothesis that 1 factor is sufficient.
##
## The degrees of freedom for the null model are  10  and the objective
   function was  3.5 with Chi Square of  125913.2
```

```
## The degrees of freedom for the model are 5  and the objective function
   was  0.51
##
## The root mean square of the residuals (RMSR) is  0.09
## The df corrected root mean square of the residuals is  0.12
##
## The harmonic number of observations is  35055 with the empirical chi
   square  5225.9  with prob <  0
## The total number of observations was  36015  with Likelihood Chi Square
    =  18365.55  with prob <  0
##
## Tucker Lewis Index of factoring reliability =  0.708
## RMSEA index =  0.319  and the 90 % confidence intervals are  0.315
   0.323
## BIC =  18313.09
## Fit based upon off diagonal values = 0.98
## Measures of factor score adequacy
##                                                    MR1
## Correlation of (regression) scores with factors   0.95
## Multiple R square of scores with factors          0.91
## Minimum correlation of possible factor scores     0.82
```

```
# variable poltrst2 is a new variable operationalizing political trust
ESS9Dat2$poltrst2<-pol.trust2$scores


# Creating a new variable "social trust"
soc.trust<-fa(as.matrix(ESS9Dat2[, c("ppltrst",#Most people can be
                                               #trusted or you can't
                                               #be too careful
                                     "pplfair",#Most people try to
                                               #take advantage of you,
                                               #or try to be fair
                                     "pplhlp")]),#Most of the time
                                                 #people helpful
                                                 #or mostly looking
                                                 #out for themselves
              nfactors=1,
              weight=ESS9Dat2$dweight,
              residuals=TRUE)

summary(soc.trust)
```

```
##
## Factor analysis with Call: fa(r = as.matrix(ESS9Dat2[, c("ppltrst", "
   pplfair", "pplhlp")]),
##     nfactors = 1, residuals = TRUE, weight = ESS9Dat2$dweight)
##
## Test of the hypothesis that 1 factor is sufficient.
## The degrees of freedom for the model is 0  and the objective function
   was  0
## The number of observations was  36015  with Chi Square =  0  with prob
   <  NA
##
```

```
## The root mean square of the residuals (RMSA) is  0
## The df corrected root mean square of the residuals is  NA
##
## Tucker Lewis Index of factoring reliability =  -Inf
```

```
loadings(soc.trust)
```

```
##
## Loadings:
##          MR1
## ppltrst 0.751
## pplfair 0.802
## pplhlp  0.711
##
##                  MR1
## SS loadings    1.712
## Proportion Var 0.571
```

```
print.psych(soc.trust,cut=0.3,sort=TRUE)
```

```
## Factor Analysis using method =  minres
## Call: fa(r = as.matrix(ESS9Dat2[, c("ppltrst", "pplfair", "pplhlp")]),
##     nfactors = 1, residuals = TRUE, weight = ESS9Dat2$dweight)
## Standardized loadings (pattern matrix) based upon correlation matrix
##         V  MR1   h2   u2 com
## pplfair 2 0.80 0.64 0.36   1
## ppltrst 1 0.75 0.56 0.44   1
## pplhlp  3 0.71 0.50 0.50   1
##
##                 MR1
## SS loadings    1.71
## Proportion Var 0.57
##
## Mean item complexity =  1
## Test of the hypothesis that 1 factor is sufficient.
##
## The degrees of freedom for the null model are  3  and the objective
   function was  0.93 with Chi Square of  33532.4
## The degrees of freedom for the model are 0  and the objective function
   was  0
##
## The root mean square of the residuals (RMSR) is  0
## The df corrected root mean square of the residuals is  NA
##
## The harmonic number of observations is  35783 with the empirical chi
   square  0  with prob <  NA
## The total number of observations was  36015  with Likelihood Chi Square
   =  0  with prob <  NA
##
## Tucker Lewis Index of factoring reliability =  -Inf
## Fit based upon off diagonal values = 1
## Measures of factor score adequacy
##                                                   MR1
```

```
## Correlation of (regression) scores with factors   0.90
## Multiple R square of scores with factors          0.80
## Minimum correlation of possible factor scores     0.61
```

```
# variable soctrst is a new variable operationalizing social trust
ESS9Dat2$soctrst<-soc.trust$scores


# Creating a new variable "external political efficacy"
pol.efficacy<-fa(as.matrix(ESS9Dat2[, c("psppsgva",#Political system
                                         #allows people
                                         #to have a say in
                                         #what government does
                                   "psppipla")]),#Political system
                                              #allows people
                                              #to have influence
                                              #on politics
                 nfactors=1,
                 weight=ESS9Dat2$dweight,
                 residuals=TRUE)

summary(pol.efficacy)
```

```
##
## Factor analysis with Call: fa(r = as.matrix(ESS9Dat2[, c("psppsgva", "
   psppipla")]), nfactors = 1,
##     residuals = TRUE, weight = ESS9Dat2$dweight)
##
## Test of the hypothesis that 1 factor is sufficient.
## The degrees of freedom for the model is -1  and the objective function
   was  0
## The number of observations was  36015  with Chi Square =  0  with prob
   <  NA
##
## The root mean square of the residuals (RMSA) is  0
## The df corrected root mean square of the residuals is  NA
##
## Tucker Lewis Index of factoring reliability =  1
```

```
loadings(pol.efficacy)
```

```
##
## Loadings:
##          MR1
## psppsgva 0.751
## psppipla 0.751
##
##                  MR1
## SS loadings    1.127
## Proportion Var 0.563
```

```
print.psych(pol.efficacy,cut=0.3,sort=TRUE)
```

```
## Factor Analysis using method =  minres
## Call: fa(r = as.matrix(ESS9Dat2[, c("psppsgva", "psppipla")]), nfactors
    = 1,
##     residuals = TRUE, weight = ESS9Dat2$dweight)
## Standardized loadings (pattern matrix) based upon correlation matrix
##          V  MR1   h2   u2 com
## psppsgva 1 0.75 0.56 0.44   1
## psppipla 2 0.75 0.56 0.44   1
##
##                 MR1
## SS loadings    1.13
## Proportion Var 0.56
##
## Mean item complexity =  1
## Test of the hypothesis that 1 factor is sufficient.
##
## The degrees of freedom for the null model are  1  and the objective
   function was  0.38 with Chi Square of  13754.95
## The degrees of freedom for the model are -1  and the objective function
    was  0
##
## The root mean square of the residuals (RMSR) is  0
## The df corrected root mean square of the residuals is  NA
##
## The harmonic number of observations is  34731 with the empirical chi
   square  0  with prob <  NA
## The total number of observations was  36015  with Likelihood Chi Square
    =  0  with prob <  NA
##
## Tucker Lewis Index of factoring reliability =  1
## Fit based upon off diagonal values = 1
## Measures of factor score adequacy
##                                                  MR1
## Correlation of (regression) scores with factors   0.85
## Multiple R square of scores with factors          0.72
## Minimum correlation of possible factor scores     0.44
```

```
# variable poleffic is a new variable operationalizing external
# political efficacy
ESS9Dat2$poleffic<-pol.efficacy$scores



# Creating a new variable "internal political efficacy"
resources<-fa(as.matrix(ESS9Dat2[, c("actrolga",#Able to take active
                                             #role in political group
                                   "cptppola")]),#Confident in own
                                                #ability to participate
                                                #in politics
              nfactors=1,
              weight=ESS9Dat2$dweight,
              residuals=TRUE)
summary(resources)
```

```
##
## Factor analysis with Call: fa(r = as.matrix(ESS9Dat2[, c("actrolga", "
   cptppola")]), nfactors = 1,
##     residuals = TRUE, weight = ESS9Dat2$dweight)
##
## Test of the hypothesis that 1 factor is sufficient.
## The degrees of freedom for the model is -1  and the objective function
   was  0
## The number of observations was  36015  with Chi Square =  0  with prob
   <  NA
##
## The root mean square of the residuals (RMSA) is  0
## The df corrected root mean square of the residuals is  NA
##
## Tucker Lewis Index of factoring reliability =  1
```

```
loadings(resources)
```

```
##
## Loadings:
##          MR1
## actrolga 0.757
## cptppola 0.757
##
##                  MR1
## SS loadings    1.145
## Proportion Var 0.573
```

```
print.psych(resources,cut=0.3,sort=TRUE)
```

```
## Factor Analysis using method =  minres
## Call: fa(r = as.matrix(ESS9Dat2[, c("actrolga", "cptppola")]), nfactors
    = 1,
##     residuals = TRUE, weight = ESS9Dat2$dweight)
## Standardized loadings (pattern matrix) based upon correlation matrix
##          V  MR1   h2   u2 com
## actrolga 1 0.76 0.57 0.43   1
## cptppola 2 0.76 0.57 0.43   1
##
##                 MR1
## SS loadings    1.15
## Proportion Var 0.57
##
## Mean item complexity =  1
## Test of the hypothesis that 1 factor is sufficient.
##
## The degrees of freedom for the null model are  1  and the objective
   function was  0.4 with Chi Square of  14305.29
## The degrees of freedom for the model are -1  and the objective function
    was  0
##
## The root mean square of the residuals (RMSR) is  0
```

```
## The df corrected root mean square of the residuals is  NA
##
## The harmonic number of observations is  34673 with the empirical chi
   square  0  with prob <  NA
## The total number of observations was  36015  with Likelihood Chi Square
    =  0  with prob <  NA
##
## Tucker Lewis Index of factoring reliability =  1
## Fit based upon off diagonal values = 1
## Measures of factor score adequacy
##                                                  MR1
## Correlation of (regression) scores with factors   0.85
## Multiple R square of scores with factors          0.73
## Minimum correlation of possible factor scores     0.46
```

```
# variable resource is a new variable operationalizing internal
# political efficacy
ESS9Dat2$resource<-resources$scores
```

Discretizing variables

In order to perform Bayesian structure learning, all the variables were discretized as suggested by Scutari and Denis (2014).

```
# Selecting the variables needed for the structure learning
# Here, we exclude the weight and variables used for the factor analysis
data<-subset(ESS9Dat2,select=c(soctrst,poltrst2,poleffic,resource,sgnptit,
                              pstplonl,polintr,clsprty,hincfel,pdwrk,
                              wrkorg,rlgblg,dscrgrp,mbtru,brncntr,
                              gndr,agea100,eduyrs100,lrscale,cntry))
```

Variables that were received as a result of factor analysis must be discretized as follows. Those values that are less than 0 are lower levels of political and social trust and internal and external political efficacy, while those values that are greater or equal to 0 are higher levels of political and social trust and internal and external political efficacy.

```
# Here I use a simple loop to discretize those variables
for (i in 1:4){
  data[,i]<-ifelse(data[,i]<0,0,1)
}
```

The rest of the variables are discretized as follows.

```
# "Placement on the left-right" is discretized as follows
# values that are <5 are a placement on the left of the scale
# values that are =5 are a placement in the center of the scale
# values that are >5 are a placement on the right of the scale
data$lrscale<-ifelse(data$lrscale<5,1,data$lrscale)
data$lrscale<-ifelse(data$lrscale==5,2,data$lrscale)
data$lrscale<-ifelse(data$lrscale>5,3,data$lrscale)

# "Years of education" are discretized as follows
# Education that is <6 years is primary education
```

```
# Education that is <11 years is secondary education
# Education that is <16 years is undergraduate education
# Education that is >16 years is graduate+ education
data$eduyrs100<-ifelse(data$eduyrs100<0.06,1,data$eduyrs100)
data$eduyrs100<-ifelse(data$eduyrs100<0.11,2,data$eduyrs100)
data$eduyrs100<-ifelse(data$eduyrs100<0.16,3,data$eduyrs100)
data$eduyrs100<-ifelse(data$eduyrs100<1,4,data$eduyrs100)

# "Age" is discretized as follows
# The first age group is between 15 and 30 y.o.
# The second age group is between 31 and 45 y.o.
# The third age group is between 46 and 60 y.o.
# The forth age group is >61 y.o.
data$agea100<-ifelse(data$agea100 >= 0.15 & data$agea100 <= 0.30, 1,
                         data$agea100)
data$agea100<-ifelse(data$agea100 >= 0.31 & data$agea100 <= 0.45, 2,
                         data$agea100)
data$agea100<-ifelse(data$agea100 >= 0.46 & data$agea100 <= 0.60, 3,
                         data$agea100)
data$agea100<-ifelse(data$agea100 >= 0.61 & data$agea100 <= 0.90, 4,
                         data$agea100)
```

```
# all variables are of the factor type
for (i in 1:20){
  data[,i]<-as.factor(as.character(data[,i]))
}
```

The next piece of code creates two datasets. One is the main dataset used for the analysis. In this dataset, participation in online activism is a “response” variable.

The second dataset is used for the robustness test. Here, participation in petition signing is a “respose” variable.

```
# This is the main dataset that will be used to explore the causal
# relationships between the variables associated with participation
# in online activism
d<-subset(data,select=c(soctrst,poltrst2,poleffic,resource,
                           pstplonl,polintr,clsprty,hincfel,pdwrk,
                           wrkorg,rlgblg,dscrgrp,mbtru,brncntr,
                           gndr,agea100,eduyrs100,lrscale,cntry))

# Dropping all of the lines with the missing values
d<-na.omit(d)

# This is the dataset that will be used to explore the causal
   relationships
# between the variables associated with participation in signing petitions
# That dataset will used for the robustness test
l<-subset(data,select=c(soctrst,poltrst2,poleffic,resource,
                           sgnptit,polintr,clsprty,hincfel,pdwrk,
                           wrkorg,rlgblg,dscrgrp,mbtru,brncntr,
                           gndr,agea100,eduyrs100,lrscale,cntry))

# Dropping all of the lines with the missing values
l<-na.omit(l)
```

```
# Saving the datasets
save(d,l,data,ESS9Dat2,file="Dataset.RData")
```

Structure learning

Bnlearn package (Scutari and Ness 2019) is used to perform Bayesian network structure learning.

When learning the structure, constraint-based, score-based and hybrid algorithms within the package (Scutari and Ness 2019) were applied and scores of the received networks were compared. Network scores show the goodness-of-fit of models (Scutari and Denis 2014). Structures with the highest network scores were retained for the further analysis.

In this study, score-based Tabu (Glover 1989) and hybrid H2PC (Gasse, Aussem, and Elghazel 2014) algorithms produced models with the highest network scores. The code below shows how the algorithms were applied and averaged.

Model averaging is usually applied to receive a better predictive performance (Scutari and Denis 2014). In the code the models are learned on the sets of 5000 network structures applying Tabu and H2PC algorithms.

```
library(bnlearn)

# Firstly, the blacklist must be defined.
# The blacklist includes all the edges incoming to the following nodes:
# "country of residence", "born in the country", "age" and "gender".

# here, the names of all nodes are assigned to the variable p
p<-names(d)

# Variable "Country" cannot be a child
# including all the esges incoming to the variable
a<-rep("cntry",length(p)-1)
blacklist<-data.frame(p[p!=a[1]],a)

# Variable "Gender" cannot be a child
a<-rep("gndr",length(p)-1)
blacklist<-rbind(blacklist,data.frame(p[p!=a[1]],a))

# Variable "Born in the country" cannot be a child
a<-rep("brncntr",length(p)-1)
blacklist<-rbind(blacklist,data.frame(p[p!=a[1]],a))

# Variable "Age" cannot be a child
a<-rep("agea100",length(p)-1)
blacklist<-rbind(blacklist,data.frame(p[p!=a[1]],a))
names(blacklist)<-c("from","to")
# New object blacklist is created
```

```
# Learning the structure applying TABU algorithm
struR1 <- tabu(d, blacklist = blacklist)
arcs(struR1) #looking at the arcs
```

```
##        from        to
##  [1,] "agea100"   "pdwrk"
##  [2,] "cntry"     "rlgblg"
##  [3,] "resource"  "polintr"
##  [4,] "poltrst2"  "poleffic"
##  [5,] "cntry"     "soctrst"
##  [6,] "cntry"     "hincfel"
##  [7,] "cntry"     "poltrst2"
##  [8,] "cntry"     "eduyrs100"
##  [9,] "cntry"     "mbtru"
## [10,] "agea100"   "mbtru"
## [11,] "cntry"     "wrkorg"
## [12,] "pdwrk"     "eduyrs100"
## [13,] "polintr"   "clsprty"
## [14,] "poleffic"  "resource"
## [15,] "soctrst"   "poltrst2"
## [16,] "cntry"     "resource"
## [17,] "clsprty"   "lrscale"
## [18,] "cntry"     "poleffic"
## [19,] "resource"  "pstplonl"
## [20,] "cntry"     "polintr"
## [21,] "resource"  "wrkorg"
## [22,] "agea100"   "pstplonl"
## [23,] "cntry"     "lrscale"
## [24,] "eduyrs100" "hincfel"
## [25,] "polintr"   "pstplonl"
## [26,] "pstplonl"  "dscrgrp"
## [27,] "agea100"   "clsprty"
## [28,] "agea100"   "rlgblg"
## [29,] "poltrst2"  "clsprty"
## [30,] "hincfel"   "soctrst"
## [31,] "mbtru"     "pdwrk"
## [32,] "gndr"      "pdwrk"
## [33,] "brncntr"   "dscrgrp"
## [34,] "agea100"   "polintr"
## [35,] "gndr"      "resource"
## [36,] "poltrst2"  "dscrgrp"
## [37,] "wrkorg"    "pstplonl"
## [38,] "pstplonl"  "clsprty"
## [39,] "polintr"   "wrkorg"
## [40,] "resource"  "dscrgrp"
```

```
# Learning the structure applying H2PC algorithm
struR2 <- h2pc(d, blacklist = blacklist)
arcs(struR2)
```

```
##        from        to
##  [1,] "agea100"   "pdwrk"
##  [2,] "cntry"     "rlgblg"
##  [3,] "resource"  "polintr"
##  [4,] "poltrst2"  "poleffic"
##  [5,] "cntry"     "soctrst"
##  [6,] "cntry"     "hincfel"
```

```
##   [7,] "cntry"      "poltrst2"
##   [8,] "cntry"      "eduyrs100"
##   [9,] "cntry"      "mbtru"
##  [10,] "agea100"    "mbtru"
##  [11,] "cntry"      "wrkorg"
##  [12,] "pdwrk"      "eduyrs100"
##  [13,] "polintr"    "clsprty"
##  [14,] "poleffic"   "resource"
##  [15,] "soctrst"    "poltrst2"
##  [16,] "cntry"      "resource"
##  [17,] "clsprty"    "lrscale"
##  [18,] "cntry"      "poleffic"
##  [19,] "resource"   "pstplonl"
##  [20,] "cntry"      "polintr"
##  [21,] "resource"   "wrkorg"
##  [22,] "agea100"    "pstplonl"
##  [23,] "cntry"      "lrscale"
##  [24,] "eduyrs100" "hincfel"
##  [25,] "polintr"    "pstplonl"
##  [26,] "agea100"    "rlgblg"
##  [27,] "cntry"      "dscrgrp"
##  [28,] "poltrst2"   "clsprty"
##  [29,] "hincfel"    "soctrst"
##  [30,] "mbtru"      "pdwrk"
##  [31,] "gndr"       "pdwrk"
##  [32,] "agea100"    "polintr"
##  [33,] "gndr"       "resource"
##  [34,] "wrkorg"     "pstplonl"
##  [35,] "brncntr"    "dscrgrp"
##  [36,] "resource"   "clsprty"
##  [37,] "polintr"    "wrkorg"
```

```
# Comparing the scores of two networks
cat("The network score of the sctructure learned by TABU is ", score(
   struR1,d),
".\nThe network score of the sctructure learned by H2PC is ",score(struR1,
   d),".\n")
```

```
## The network score of the sctructure learned by TABU is  -391029.4 .
## The network score of the sctructure learned by H2PC is  -391029.4 .
```

```
# TABU and H2PC algorithms produced the highest network scores in
# comparison to other algorithms that were used but not reported here
# Depending on the dataset, try other algorithms and see which ones are
# able to learn the structures of the networks with the highest scores

# Model averaging is required to receive more reliable structures
# Applying model averaging to the structure received as the result of
# using TABU algorithm
str.diff = boot.strength(d, R = 5000, algorithm = "tabu",
                         algorithm.args = list(blacklist = blacklist))
avg.diff = averaged.network(str.diff)
```

```
# Applying model averaging to the structure received as the result of
# using H2PC algorithm
str.diff2 = boot.strength(d, R = 5000, algorithm = "h2pc",
                          algorithm.args = list(blacklist = blacklist))
avg.diff2 = averaged.network(str.diff2)

save(avg.diff,avg.diff2,file="BNdata.RData")
```

Vizualizing and comparing the models

Two models were compared using the tools of bnlearn (Scutari and Ness 2019) package.

```
compare(avg.diff,avg.diff2, arcs = TRUE)
```

```
## $tp
##        from        to
##  [1,] "poltrst2"  "poleffic"
##  [2,] "poltrst2"  "clsprty"
##  [3,] "resource"  "pstplonl"
##  [4,] "resource"  "polintr"
##  [5,] "resource"  "wrkorg"
##  [6,] "pstplonl"  "clsprty"
##  [7,] "pstplonl"  "dscrgrp"
##  [8,] "polintr"   "pstplonl"
##  [9,] "polintr"   "clsprty"
## [10,] "clsprty"   "lrscale"
## [11,] "pdwrk"     "eduyrs100"
## [12,] "wrkorg"    "pstplonl"
## [13,] "brncntr"   "dscrgrp"
## [14,] "agea100"   "pstplonl"
## [15,] "agea100"   "polintr"
## [16,] "agea100"   "clsprty"
## [17,] "agea100"   "pdwrk"
## [18,] "agea100"   "rlgblg"
## [19,] "agea100"   "mbtru"
## [20,] "eduyrs100" "hincfel"
## [21,] "cntry"     "soctrst"
## [22,] "cntry"     "poltrst2"
## [23,] "cntry"     "poleffic"
## [24,] "cntry"     "resource"
## [25,] "cntry"     "polintr"
## [26,] "cntry"     "hincfel"
## [27,] "cntry"     "pdwrk"
## [28,] "cntry"     "wrkorg"
## [29,] "cntry"     "rlgblg"
## [30,] "cntry"     "mbtru"
## [31,] "cntry"     "eduyrs100"
## [32,] "cntry"     "lrscale"
##
## $fp
##       from       to
## [1,] "soctrst"  "poltrst2"
## [2,] "poleffic" "resource"
```

```
## [3,] "resource" "eduyrs100"
## [4,] "gndr"      "resource"
##
## $fn
##       from        to
## [1,] "poltrst2"  "soctrst"
## [2,] "poltrst2"  "dscrgrp"
## [3,] "resource"  "poleffic"
## [4,] "resource"  "dscrgrp"
## [5,] "polintr"   "wrkorg"
## [6,] "eduyrs100" "resource"
```

One way to vizualize the and compare the networks is to use DiagrammeR package (Iannone 2020). All the arcs and nodes illustrated by DiagrammeR must be specified manually.

```
library("DiagrammeR") # for vizualization
library(dplyr) # for merging dataframes
```

```
# Here, we extract the list of all arcs found by TABU
tabu_arcs<-as.data.frame(arcs(avg.diff))
# We want to exclude the node "Country" from the vizualized network
tabu_arcs<-subset(tabu_arcs,tabu_arcs$from!="cntry")

# Creating a dataframe that contains all node names and node numbers
# When vizualizing using DiagrammeR all nodes must be numerated
nodes<-names(d)
node_number<-1:19 # giving a number to each node
nodes<-data.frame(nodes,node_number) #creating a dataframe

# Now we can use left join to add two colums that will present the same
# arcs but instead of node names, node numbers will be used
names(nodes)<-c("from","a") # renaming column names to apply left join
tabu_arcs<-left_join(tabu_arcs,nodes)
names(nodes)<-c("to","b") # renaming column names to apply left join
                          #by column "to"
tabu_arcs<-left_join(tabu_arcs,nodes)
```

```
# To receive a clean list of all arcs that we can just copy paste
# into DiagrammeR
# We will create one more column
tabu_arcs$c<-paste0(tabu_arcs$a,"->",tabu_arcs$b)
paste(tabu_arcs$c, collapse=' ' ) # see the output
```

```
## [1] "2->1 2->3 2->7 2->12 4->3 4->5 4->6 4->10 4->12 5->7 5->12 6->5
   6->7 6->10 7->18 9->17 10->5 14->12 16->5 16->6 16->7 16->9 16->11
   16->13 17->4 17->8"
```

```
# now we can use the output to paste into the DiagrammeR code
```

```
# Specifying all the nodes and arcs in DiagrammeR

DiagrammeR::grViz("digraph dot{
```

```
  graph[layout = dot, rankdir = TB,overlap=TRUE,fontsize=72,label='DAG 1.
   Online
                  patricipation. Structure learned by TABU', labelloc=t]
  node[shape=circle, fontsize=46, fontcolor=Black, fillcolor = Salmon1,
   style = filled]
5 [label = 'Participation online', fontsize=46]
node[shape=egg, fontsize=32, fontcolor=Black,fillcolor = Turquoise2, style
    = filled]
16 [label = 'Age', fontsize=32]
15 [label = 'Gender', fontsize=32]
14 [label = 'Born in the country', fontsize=32]
node[shape=box,fontcolor=Black, fillcolor= Turquoise1, fontsize=32]
1 [label = 'Social trust']
2 [label = 'Political trust']
3 [label = 'External political efficacy']
4 [label = 'Internal political efficacy']
6 [label = 'Political interest']
7 [label = 'Party identification']
8 [label = 'Income']
9 [label = 'Being in workforce']
10 [label = 'Working in a non-governmental organization']
11 [label = 'Belonging to particular religion']
12 [label = 'Self-identifying as a part of a discriminated group']
13 [label = 'Membership in a trade union']
17 [label = 'Education']
18 [label = 'Placement on the left-right scale']

2->1 2->3 2->7 2->12 4->3 4->5 4->6 4->10 4->12 5->7 5->12 6->5 6->7 6->10
    7->18
9->17 10->5 14->12 16->5 16->6 16->7 16->9 16->11 16->13 17->4 17->8

}")
```

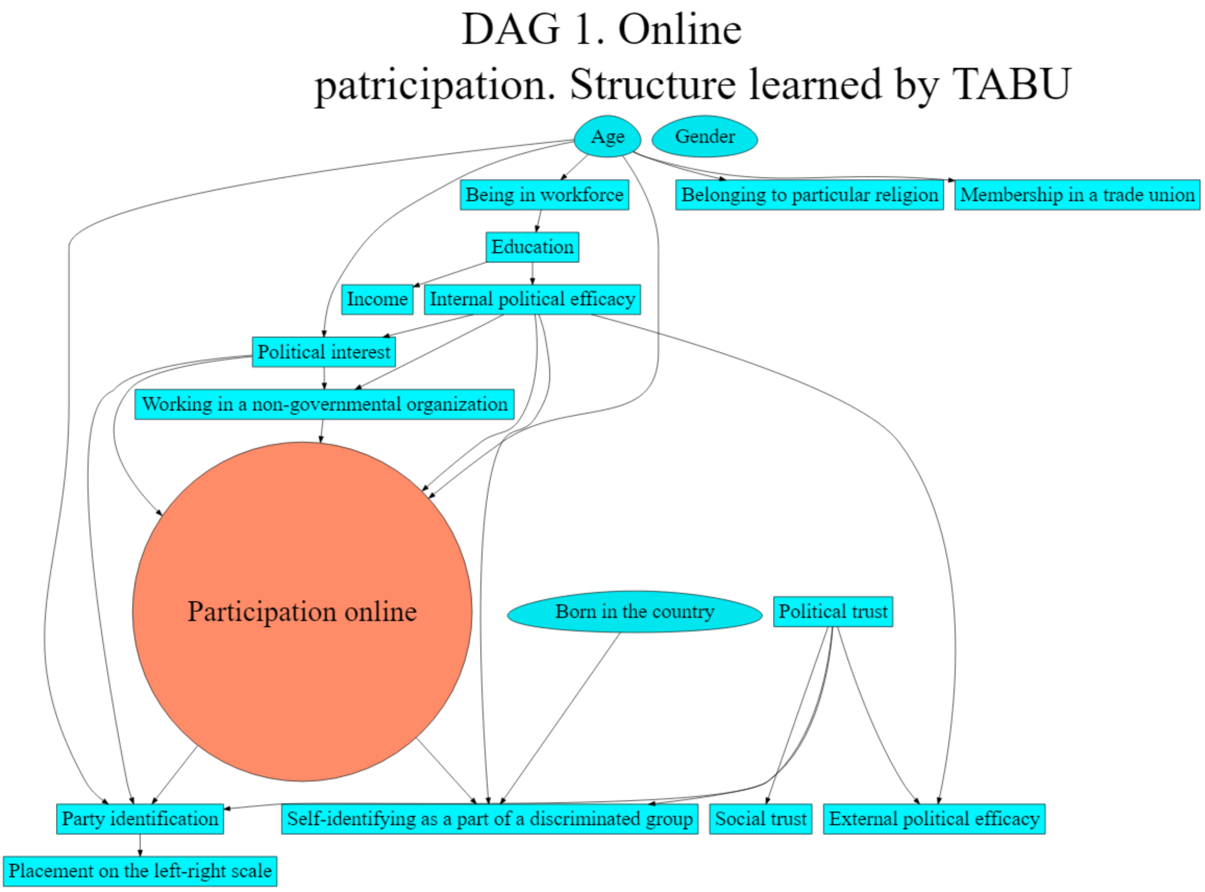


```
# The same actions are taken to receive a list of all arcs from the
# structure learned by H2PC

tabu_arcs2<-as.data.frame(arcs(avg.diff2))
tabu_arcs2<-subset(tabu_arcs2,tabu_arcs2$from!="cntry")

# Now we can use left join to add two colums that will present the same
# arcs but instead of node names, node numbers will be used
names(nodes)<-c("from","a") # renaming column names to apply left join
                             # by column "from"
tabu_arcs2<-left_join(tabu_arcs2,nodes)
names(nodes)<-c("to","b") # renaming column names to apply left
                           # join by column "to"
tabu_arcs2<-left_join(tabu_arcs2,nodes)

tabu_arcs2$c<-paste0(tabu_arcs2$a,"->",tabu_arcs2$b)
```

```
paste(tabu_arcs2$c, collapse=' ') # see the output
```

```
## [1] "1->2 2->3 2->7 3->4 4->5 4->6 4->10 4->17 5->7 5->12 6->5 6->7
    7->18 9->17 10->5 14->12 15->4 16->5 16->6 16->7 16->9 16->11 16->13
    17->8"
```

```
# now we can use the output to paste into the DiagrammeR code
```

```
# Specifying all the nodes and arcs in DiagrammeR
DiagrammeR::grViz("digraph dot{
  graph[layout = dot, rankdir = TB,overlap=TRUE,fontsize=72,label='DAG 2.
                  Online patricipation. Structure learned by H2PC',
   labelloc=t]
  node[shape=circle, fontsize=46, fontcolor=Black, fillcolor = Salmon1,
   style = filled]
5 [label = 'Participation online', fontsize=46]
node[shape=egg, fontsize=32, fontcolor=Black,fillcolor = Turquoise2, style
    = filled]
16 [label = 'Age', fontsize=32]
15 [label = 'Gender', fontsize=32]
14 [label = 'Born in the country', fontsize=32]
node[shape=box,fontcolor=Black, fillcolor= Turquoise1, fontsize=32]
1 [label = 'Social trust']
2 [label = 'Political trust']
3 [label = 'External political efficacy']
4 [label = 'Internal political efficacy']
6 [label = 'Political interest']
7 [label = 'Party identification']
8 [label = 'Income']
9 [label = 'Being in workforce']
10 [label = 'Working in a non-governmental organization']
11 [label = 'Belonging to particular religion']
12 [label = 'Self-identifying as a part of a discriminated group']
13 [label = 'Membership in a trade union']
17 [label = 'Education']
18 [label = 'Placement on the left-right scale']

1->2 2->3 2->7 3->4 4->5 4->6 4->10 4->17 5->7 5->12 6->5 6->7 7->18 9->17
    10->5
14->12 15->4 16->5 16->6 16->7 16->9 16->11 16->13 17->8
}")
```

DAG 2.
Online patrication. Structure learned by H2PC

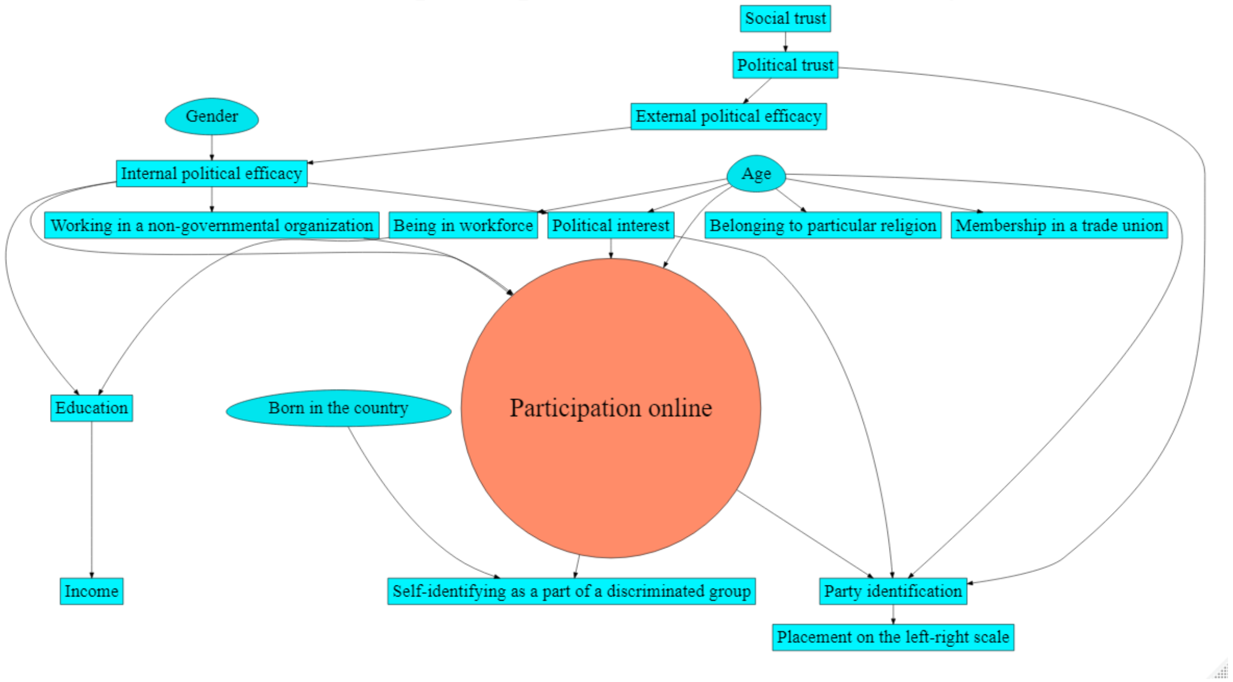


```
# We can also show false negatives and false positives in the figures
# The following code is used to extract false negatives and false
# positives to highlight the arcs in DiagrammeR figure

#Extracting false positives
false_posit<-as.data.frame(compare(avg.diff, avg.diff2, arcs = TRUE)$fp)
names(nodes)<-c("from","a")
false_posit<-left_join(false_posit,nodes)
names(nodes)<-c("to","b")
false_posit<-left_join(false_posit,nodes)
false_posit$c<-paste0(false_posit$a,"->",false_posit$b)


#Extracting false negatives
false_negat<-as.data.frame(compare(avg.diff, avg.diff2, arcs = TRUE)$fn)
names(nodes)<-c("from","a")
false_negat<-left_join(false_negat,nodes)
names(nodes)<-c("to","b")
false_negat<-left_join(false_negat,nodes)
false_negat$c<-paste0(false_negat$a,"->",false_negat$b)
```

```
cat("False positives are ",paste(false_posit$c, collapse=’ ’),"\n",
    "False negatives are ",paste(false_negat$c, collapse=’ ’),"\n")
```

```
## False positives are  1->2 3->4 4->17 15->4
##  False negatives are  2->1 2->12 4->3 4->12 6->10 17->4
```

```
DiagrammeR::grViz("digraph dot{
  graph[layout = dot, rankdir = TB,overlap=TRUE,fontsize=72,label='Online
   patricipation.
                 Compartison of the structures learned by TABU and H2PC',
   labelloc=t]
  node[shape=circle, fontsize=46, fontcolor=Black, fillcolor = Salmon1,
   style = filled]
5 [label = 'Participation online', fontsize=46]
node[shape=egg, fontsize=32, fontcolor=Black,fillcolor = Turquoise2, style
    = filled]
16 [label = 'Age', fontsize=32]
15 [label = 'Gender', fontsize=32]
14 [label = 'Born in the country', fontsize=32]
node[shape=box,fontcolor=Black, fillcolor= Turquoise1, fontsize=32]
1 [label = 'Social trust']
2 [label = 'Political trust']
3 [label = 'External political efficacy']
4 [label = 'Internal political efficacy']
6 [label = 'Political interest']
7 [label = 'Party identification']
8 [label = 'Income']
9 [label = 'Being in workforce']
10 [label = 'Working in a non-governmental organization']
11 [label = 'Belonging to particular religion']
12 [label = 'Self-identifying as a part of a discriminated group']
13 [label = 'Membership in a trade union']
17 [label = 'Education']
18 [label = 'Placement on the left-right scale']

2->1[color=DarkOrange2,penwidth=5] 2->12[color=DarkOrange2,penwidth=5]
4->3[color=DarkOrange2,penwidth=5] 4->12[color=DarkOrange2,penwidth=5]
6->10[color=DarkOrange2,penwidth=5] 17->4[color=DarkOrange2,penwidth=5]
1->2[color= DarkTurquoise, style=dashed,penwidth=5]
3->4[color= DarkTurquoise, style=dashed,penwidth=5]
4->17[color= DarkTurquoise, style=dashed,penwidth=5]
15->4[color= DarkTurquoise, style=dashed,penwidth=5]
2->3 2->7 4->5 4->6 4->10 5->7 5->12 6->5 6->7 7->18 9->17 10->5 14->12
16->5 16->6 16->7 16->9 16->11 16->13 17->8

}")
```

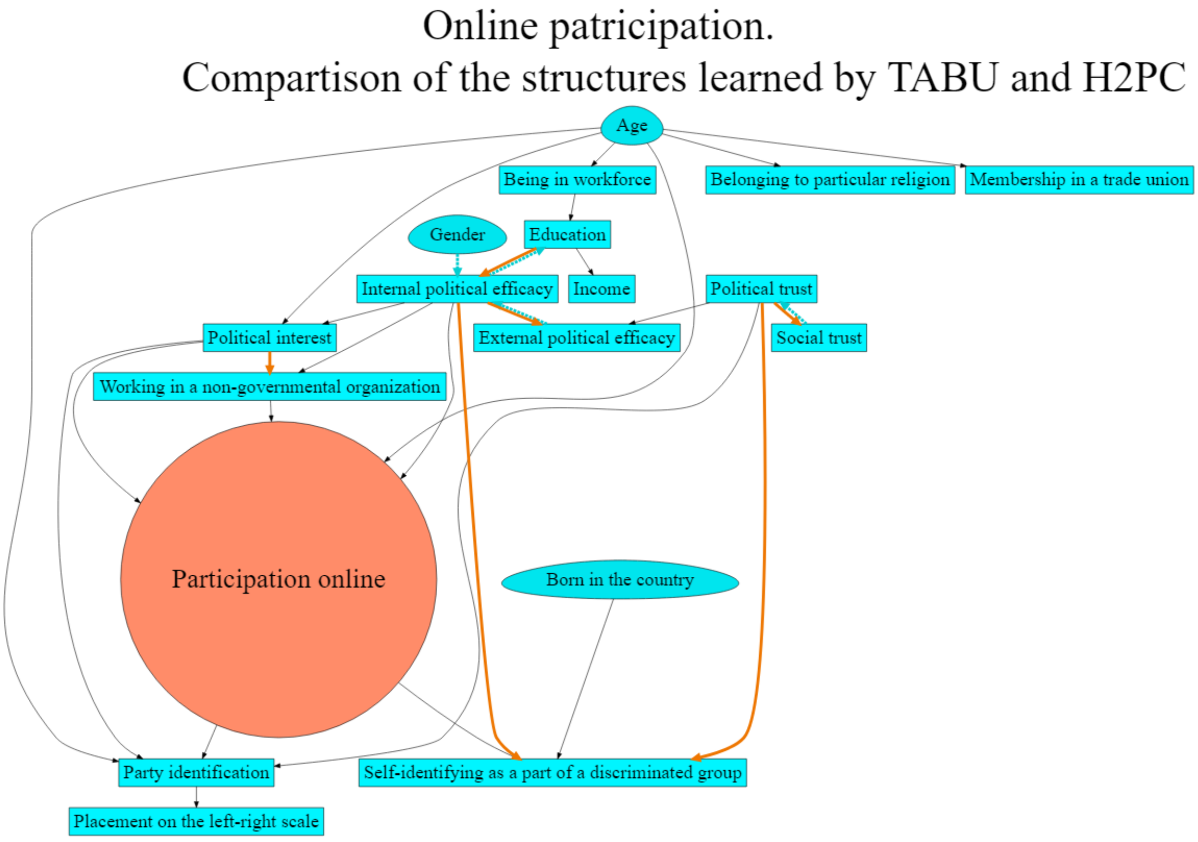

In the article, other methods of vizualization are used. In particular, I have used yEd Graph Editor. Please, visit https://www.yworks.com/products/yed for details.

Robustness check

As a robustness check the same steps were taken to examine the causal relationships between the variables associated with another type of non-institutionalised political participation, i.e., "Participation in signing petitions" (Marien, Hooghe, and Quintelier 2010). Thus, to learn the structure of the network all the nodes, except for "Participation in online activism" were retained, while the node "Participation in signing petitions" was added. In that way, the structures were learned on another subset of data, as not all the people who participate in petition signing also participate in online activism.

```
# The dataset used for the robustness check is l that was created earlier

# For learning the structure, we need to create a new blacklist, that
# is the same as the first one but pstplonl ("Participation in online
# activism") is replaced by sgnptit ("Participation in signing petitions")

p<-names(l)
# Variable "Country" cannot be a child
a<-rep("cntry",length(p)-1)
blacklist2<-data.frame(p[p!=a[1]],a)

# Variable "Gender" cannot be a child
```

```
a<-rep("gndr",length(p)-1)
blacklist2<-rbind(blacklist2,data.frame(p[p!=a[1]],a))

# Variable "Born in the country" cannot be a child
a<-rep("brncntr",length(p)-1)
blacklist2<-rbind(blacklist2,data.frame(p[p!=a[1]],a))

# Variable "Age" cannot be a child
a<-rep("agea100",length(p)-1)
blacklist2<-rbind(blacklist2,data.frame(p[p!=a[1]],a))
names(blacklist2)<-c("from","to")
# New object blacklist2 is created
```

```
# Learning the structure using TABU algorithm
struR3 <- tabu(l, blacklist = blacklist2)

# Learning the structure using H2PC algorithm
struR4 <- h2pc(l, blacklist = blacklist2)

# Model averaging is required to receive more reliable structures
# Applying model averaging to the structure received as the result of
# using TABU algorithm
str.diff3 = boot.strength(l, R = 5000, algorithm = "tabu",
                          algorithm.args = list(blacklist = blacklist2))
avg.diff3 = averaged.network(str.diff3)

# Applying model averaging to the structure received as the result of
# using H2PC algorithm
str.diff4 = boot.strength(l, R = 5000, algorithm = "h2pc",
                          algorithm.args = list(blacklist = blacklist2))
avg.diff4 = averaged.network(str.diff4)
```

```
# Examining the received arcs. Arcs of the network learned by TABU
arcs(avg.diff3)
```

```
##       from        to
##  [1,] "poltrst2"  "soctrst"
##  [2,] "poltrst2"  "poleffic"
##  [3,] "poltrst2"  "clsprty"
##  [4,] "poltrst2"  "dscrgrp"
##  [5,] "resource"  "poleffic"
##  [6,] "resource"  "sgnptit"
##  [7,] "resource"  "polintr"
##  [8,] "resource"  "clsprty"
##  [9,] "resource"  "wrkorg"
## [10,] "sgnptit"   "wrkorg"
## [11,] "sgnptit"   "dscrgrp"
## [12,] "polintr"   "sgnptit"
## [13,] "polintr"   "clsprty"
## [14,] "clsprty"   "lrscale"
## [15,] "pdwrk"     "eduyrs100"
## [16,] "brncntr"   "dscrgrp"
## [17,] "agea100"   "polintr"
```

```
## [18,] "agea100"   "clsprty"
## [19,] "agea100"   "pdwrk"
## [20,] "agea100"   "rlgblg"
## [21,] "agea100"   "mbtru"
## [22,] "eduyrs100" "resource"
## [23,] "eduyrs100" "hincfel"
## [24,] "cntry"     "soctrst"
## [25,] "cntry"     "poltrst2"
## [26,] "cntry"     "poleffic"
## [27,] "cntry"     "resource"
## [28,] "cntry"     "sgnptit"
## [29,] "cntry"     "polintr"
## [30,] "cntry"     "hincfel"
## [31,] "cntry"     "pdwrk"
## [32,] "cntry"     "wrkorg"
## [33,] "cntry"     "rlgblg"
## [34,] "cntry"     "mbtru"
## [35,] "cntry"     "eduyrs100"
## [36,] "cntry"     "lrscale"
```

```
# Arcs of the network learned by H2PC
arcs(avg.diff4)
```

```
##       from        to
##  [1,] "soctrst"   "poltrst2"
##  [2,] "poltrst2"  "poleffic"
##  [3,] "poltrst2"  "clsprty"
##  [4,] "poleffic"  "resource"
##  [5,] "resource"  "sgnptit"
##  [6,] "resource"  "polintr"
##  [7,] "resource"  "clsprty"
##  [8,] "resource"  "wrkorg"
##  [9,] "resource"  "eduyrs100"
## [10,] "sgnptit"   "wrkorg"
## [11,] "polintr"   "clsprty"
## [12,] "clsprty"   "lrscale"
## [13,] "pdwrk"     "eduyrs100"
## [14,] "brncntr"   "dscrgrp"
## [15,] "gndr"      "resource"
## [16,] "agea100"   "polintr"
## [17,] "agea100"   "clsprty"
## [18,] "agea100"   "pdwrk"
## [19,] "agea100"   "rlgblg"
## [20,] "agea100"   "mbtru"
## [21,] "eduyrs100" "hincfel"
## [22,] "cntry"     "soctrst"
## [23,] "cntry"     "poltrst2"
## [24,] "cntry"     "poleffic"
## [25,] "cntry"     "resource"
## [26,] "cntry"     "sgnptit"
## [27,] "cntry"     "polintr"
## [28,] "cntry"     "hincfel"
## [29,] "cntry"     "pdwrk"
## [30,] "cntry"     "wrkorg"
```

```
## [31,] "cntry"     "rlgblg"
## [32,] "cntry"     "dscrgrp"
## [33,] "cntry"     "mbtru"
## [34,] "cntry"     "eduyrs100"
## [35,] "cntry"     "lrscale"
```

Comparing the causal relationships between the variables associated with political participation, it became evident that the structures received using Bayesian network structure learning tools are quite reliable.

The following code shows one of the ways to compare the structures.

```
# To compare the structures, I renamed the "outcome" variables, i.e.,
# variables operationalizing political participation (online
# participation and signing petitions), as "participation"

# The nodes of the structures should have the same names to be compared
# using bnlearn tools

# Creating a list containing the names of all nodes
b1<-names(d) # working with the dataset containing the variables
# associated with online participation
b1[b1=="pstplonl"]<-"participation" # renaming the node

newdag1 <- empty.graph(nodes = b1) # creating a new graph with the
# nodes of the new dataset b1
b<- as.data.frame(arcs(avg.diff)) # taking the arcs of the 1st learned
                                  # structure
b[b$from=="pstplonl",1]<-"participation" # renaming the node pstplonl
# (online participation) to "participation"
b[b$to=="pstplonl",2]<-"participation"
arcs(newdag1) <- b # loading the arcs to the new graph

# Next, taking the same steps to compare structures of the networks
# of variables associated with online participation and signing petitions
# Creating a new DAG representing the structure avg.diff2 received by
# applying H2PC algorithm
newdag2 <- empty.graph(nodes = b1) # the same nodes are applied
b<- as.data.frame(arcs(avg.diff2))
b[b$from=="pstplonl",1]<-"participation"
b[b$to=="pstplonl",2]<-"participation"
arcs(newdag2) <- b

# Creating a new DAG representing the structure avg.diff3 received by
# applying TABU algorithm to the data containing variables associated
# with participation in petition signing
newdag3 <- empty.graph(nodes = b1)
b<- as.data.frame(arcs(avg.diff3))
b[b$from=="sgnptit",1]<-"participation"
b[b$to=="sgnptit",2]<-"participation"
arcs(newdag3) <- b

# Creating a new DAG representing the structure avg.diff3 received by
# applying H2PC algorithm to the data containing variables associated
# with participation in petition signing
newdag4 <- empty.graph(nodes = b1)
```

```
b<- as.data.frame(arcs(avg.diff4))
b[b$from=="sgnptit",1]<-"participation"
b[b$to=="sgnptit",2]<-"participation"
arcs(newdag4) <- b
```

```
# Now the DAGs can be compared as they have the same nodes
compare(newdag1,newdag3, arcs = TRUE)
```

```
## $tp
##       from            to
##  [1,] "poltrst2"      "soctrst"
##  [2,] "poltrst2"      "poleffic"
##  [3,] "poltrst2"      "clsprty"
##  [4,] "poltrst2"      "dscrgrp"
##  [5,] "resource"      "poleffic"
##  [6,] "resource"      "participation"
##  [7,] "resource"      "polintr"
##  [8,] "resource"      "wrkorg"
##  [9,] "participation" "dscrgrp"
## [10,] "polintr"       "participation"
## [11,] "polintr"       "clsprty"
## [12,] "clsprty"       "lrscale"
## [13,] "pdwrk"         "eduyrs100"
## [14,] "brncntr"       "dscrgrp"
## [15,] "agea100"       "polintr"
## [16,] "agea100"       "clsprty"
## [17,] "agea100"       "pdwrk"
## [18,] "agea100"       "rlgblg"
## [19,] "agea100"       "mbtru"
## [20,] "eduyrs100"     "resource"
## [21,] "eduyrs100"     "hincfel"
## [22,] "cntry"         "soctrst"
## [23,] "cntry"         "poltrst2"
## [24,] "cntry"         "poleffic"
## [25,] "cntry"         "resource"
## [26,] "cntry"         "polintr"
## [27,] "cntry"         "hincfel"
## [28,] "cntry"         "pdwrk"
## [29,] "cntry"         "wrkorg"
## [30,] "cntry"         "rlgblg"
## [31,] "cntry"         "mbtru"
## [32,] "cntry"         "eduyrs100"
## [33,] "cntry"         "lrscale"
##
## $fp
##      from            to
## [1,] "resource"      "clsprty"
## [2,] "participation" "wrkorg"
## [3,] "cntry"         "participation"
##
## $fn
##      from            to
## [1,] "resource"      "dscrgrp"
## [2,] "participation" "clsprty"
```

```
## [3,] "polintr"       "wrkorg"
## [4,] "wrkorg"        "participation"
## [5,] "agea100"       "participation"
```

```
compare(newdag1,newdag4, arcs = TRUE)
```

```
## $tp
##       from        to
##  [1,] "poltrst2"  "poleffic"
##  [2,] "poltrst2"  "clsprty"
##  [3,] "resource"  "participation"
##  [4,] "resource"  "polintr"
##  [5,] "resource"  "wrkorg"
##  [6,] "polintr"   "clsprty"
##  [7,] "clsprty"   "lrscale"
##  [8,] "pdwrk"     "eduyrs100"
##  [9,] "brncntr"   "dscrgrp"
## [10,] "agea100"   "polintr"
## [11,] "agea100"   "clsprty"
## [12,] "agea100"   "pdwrk"
## [13,] "agea100"   "rlgblg"
## [14,] "agea100"   "mbtru"
## [15,] "eduyrs100" "hincfel"
## [16,] "cntry"     "soctrst"
## [17,] "cntry"     "poltrst2"
## [18,] "cntry"     "poleffic"
## [19,] "cntry"     "resource"
## [20,] "cntry"     "polintr"
## [21,] "cntry"     "hincfel"
## [22,] "cntry"     "pdwrk"
## [23,] "cntry"     "wrkorg"
## [24,] "cntry"     "rlgblg"
## [25,] "cntry"     "mbtru"
## [26,] "cntry"     "eduyrs100"
## [27,] "cntry"     "lrscale"
##
## $fp
##      from            to
## [1,] "soctrst"       "poltrst2"
## [2,] "poleffic"      "resource"
## [3,] "resource"      "clsprty"
## [4,] "resource"      "eduyrs100"
## [5,] "participation" "wrkorg"
## [6,] "gndr"          "resource"
## [7,] "cntry"         "participation"
## [8,] "cntry"         "dscrgrp"
##
## $fn
##       from            to
##  [1,] "poltrst2"      "soctrst"
##  [2,] "poltrst2"      "dscrgrp"
##  [3,] "resource"      "poleffic"
##  [4,] "resource"      "dscrgrp"
##  [5,] "participation" "clsprty"
```

```
##  [6,] "participation" "dscrgrp"
##  [7,] "polintr"       "participation"
##  [8,] "polintr"       "wrkorg"
##  [9,] "wrkorg"        "participation"
## [10,] "agea100"       "participation"
## [11,] "eduyrs100"     "resource"
```

```
compare(newdag2,newdag3, arcs = TRUE)
```

```
## $tp
##       from            to
##  [1,] "poltrst2"      "poleffic"
##  [2,] "poltrst2"      "clsprty"
##  [3,] "resource"      "participation"
##  [4,] "resource"      "polintr"
##  [5,] "resource"      "wrkorg"
##  [6,] "participation" "dscrgrp"
##  [7,] "polintr"       "participation"
##  [8,] "polintr"       "clsprty"
##  [9,] "clsprty"       "lrscale"
## [10,] "pdwrk"         "eduyrs100"
## [11,] "brncntr"       "dscrgrp"
## [12,] "agea100"       "polintr"
## [13,] "agea100"       "clsprty"
## [14,] "agea100"       "pdwrk"
## [15,] "agea100"       "rlgblg"
## [16,] "agea100"       "mbtru"
## [17,] "eduyrs100"     "hincfel"
## [18,] "cntry"         "soctrst"
## [19,] "cntry"         "poltrst2"
## [20,] "cntry"         "poleffic"
## [21,] "cntry"         "resource"
## [22,] "cntry"         "polintr"
## [23,] "cntry"         "hincfel"
## [24,] "cntry"         "pdwrk"
## [25,] "cntry"         "wrkorg"
## [26,] "cntry"         "rlgblg"
## [27,] "cntry"         "mbtru"
## [28,] "cntry"         "eduyrs100"
## [29,] "cntry"         "lrscale"
##
## $fp
##      from            to
## [1,] "poltrst2"      "soctrst"
## [2,] "poltrst2"      "dscrgrp"
## [3,] "resource"      "poleffic"
## [4,] "resource"      "clsprty"
## [5,] "participation" "wrkorg"
## [6,] "eduyrs100"     "resource"
## [7,] "cntry"         "participation"
##
## $fn
##      from            to
## [1,] "soctrst"       "poltrst2"
```

```
## [2,] "poleffic"      "resource"
## [3,] "resource"      "eduyrs100"
## [4,] "participation" "clsprty"
## [5,] "wrkorg"        "participation"
## [6,] "gndr"          "resource"
## [7,] "agea100"       "participation"
```

```
compare(newdag2,newdag4, arcs = TRUE)
```

```
## $tp
##       from        to
##  [1,] "soctrst"   "poltrst2"
##  [2,] "poltrst2"  "poleffic"
##  [3,] "poltrst2"  "clsprty"
##  [4,] "poleffic"  "resource"
##  [5,] "resource"  "participation"
##  [6,] "resource"  "polintr"
##  [7,] "resource"  "wrkorg"
##  [8,] "resource"  "eduyrs100"
##  [9,] "polintr"   "clsprty"
## [10,] "clsprty"   "lrscale"
## [11,] "pdwrk"     "eduyrs100"
## [12,] "brncntr"   "dscrgrp"
## [13,] "gndr"      "resource"
## [14,] "agea100"   "polintr"
## [15,] "agea100"   "clsprty"
## [16,] "agea100"   "pdwrk"
## [17,] "agea100"   "rlgblg"
## [18,] "agea100"   "mbtru"
## [19,] "eduyrs100" "hincfel"
## [20,] "cntry"     "soctrst"
## [21,] "cntry"     "poltrst2"
## [22,] "cntry"     "poleffic"
## [23,] "cntry"     "resource"
## [24,] "cntry"     "polintr"
## [25,] "cntry"     "hincfel"
## [26,] "cntry"     "pdwrk"
## [27,] "cntry"     "wrkorg"
## [28,] "cntry"     "rlgblg"
## [29,] "cntry"     "mbtru"
## [30,] "cntry"     "eduyrs100"
## [31,] "cntry"     "lrscale"
##
## $fp
##      from            to
## [1,] "resource"      "clsprty"
## [2,] "participation" "wrkorg"
## [3,] "cntry"         "participation"
## [4,] "cntry"         "dscrgrp"
##
## $fn
##      from            to
## [1,] "participation" "clsprty"
## [2,] "participation" "dscrgrp"
```

```
## [3,] "polintr"       "participation"
## [4,] "wrkorg"        "participation"
## [5,] "agea100"       "participation"
```

I have conducted another robustness check and leaned the structures including both “Participation in signing petitions” and “Participation in online activism” as nodes.

```
p<-data # dataset data contains all of the needed variables
p<-na.omit(p)

# Creating a blacklist
z<-names(p)
# Variable "Country" cannot be a child
a<-rep("cntry",length(z)-1)
blacklist3<-data.frame(z[z!=a[1]],a)

# Variable "Gender" cannot be a child
a<-rep("gndr",length(z)-1)
blacklist3<-rbind(blacklist3,data.frame(z[z!=a[1]],a))

# Variable "Born in the country" cannot be a child
a<-rep("brncntr",length(z)-1)
blacklist3<-rbind(blacklist3,data.frame(z[z!=a[1]],a))

# Variable "Age" cannot be a child
a<-rep("agea100",length(z)-1)
blacklist3<-rbind(blacklist3,data.frame(z[z!=a[1]],a))

names(blacklist3)<-c("from","to")
# Now the new object blacklist3 is created

# Applying model averaging to the structure received as the result of
# using TABU algorithm
str.diff5 = boot.strength(p, R = 5000, algorithm = "tabu",
                          algorithm.args = list(blacklist = blacklist3))
avg.diff5 = averaged.network(str.diff5)

# Applying model averaging to the structure received as the result of
# using H2PC algorithm
str.diff6 = boot.strength(p, R = 5000, algorithm = "h2pc",
                          algorithm.args = list(blacklist = blacklist3))
avg.diff6 = averaged.network(str.diff6)
```

```
# Examining the received arcs. Arcs of the network learned by TABU
arcs(avg.diff5)
```

```
##      from       to
##  [1,] "poltrst2" "soctrst"
##  [2,] "poltrst2" "poleffic"
##  [3,] "poltrst2" "clsprty"
##  [4,] "poltrst2" "dscrgrp"
##  [5,] "resource" "poleffic"
##  [6,] "resource" "sgnptit"
##  [7,] "resource" "pstplonl"
```

```
##   [8,] "resource"  "polintr"
##   [9,] "resource"  "wrkorg"
##  [10,] "sgnptit"   "wrkorg"
##  [11,] "sgnptit"   "dscrgrp"
##  [12,] "pstplonl"  "sgnptit"
##  [13,] "pstplonl"  "clsprty"
##  [14,] "pstplonl"  "dscrgrp"
##  [15,] "polintr"   "pstplonl"
##  [16,] "polintr"   "clsprty"
##  [17,] "clsprty"   "lrscale"
##  [18,] "pdwrk"     "eduyrs100"
##  [19,] "brncntr"   "dscrgrp"
##  [20,] "agea100"   "pstplonl"
##  [21,] "agea100"   "polintr"
##  [22,] "agea100"   "clsprty"
##  [23,] "agea100"   "pdwrk"
##  [24,] "agea100"   "rlgblg"
##  [25,] "agea100"   "mbtru"
##  [26,] "eduyrs100" "resource"
##  [27,] "eduyrs100" "hincfel"
##  [28,] "cntry"     "soctrst"
##  [29,] "cntry"     "poltrst2"
##  [30,] "cntry"     "poleffic"
##  [31,] "cntry"     "resource"
##  [32,] "cntry"     "sgnptit"
##  [33,] "cntry"     "polintr"
##  [34,] "cntry"     "hincfel"
##  [35,] "cntry"     "pdwrk"
##  [36,] "cntry"     "wrkorg"
##  [37,] "cntry"     "rlgblg"
##  [38,] "cntry"     "mbtru"
##  [39,] "cntry"     "eduyrs100"
##  [40,] "cntry"     "lrscale"
```

```
# Arcs of the network learned by H2PC
arcs(avg.diff6)
```

```
##        from        to
##   [1,] "soctrst"   "poltrst2"
##   [2,] "poltrst2"  "poleffic"
##   [3,] "poltrst2"  "clsprty"
##   [4,] "poleffic"  "resource"
##   [5,] "resource"  "polintr"
##   [6,] "resource"  "clsprty"
##   [7,] "resource"  "wrkorg"
##   [8,] "sgnptit"   "pstplonl"
##   [9,] "sgnptit"   "wrkorg"
##  [10,] "pstplonl"  "resource"
##  [11,] "polintr"   "clsprty"
##  [12,] "clsprty"   "lrscale"
##  [13,] "pdwrk"     "eduyrs100"
##  [14,] "brncntr"   "dscrgrp"
##  [15,] "agea100"   "pstplonl"
##  [16,] "agea100"   "polintr"
```

```
## [17,] "agea100"   "clsprty"
## [18,] "agea100"   "pdwrk"
## [19,] "agea100"   "rlgblg"
## [20,] "agea100"   "mbtru"
## [21,] "eduyrs100" "hincfel"
## [22,] "cntry"     "soctrst"
## [23,] "cntry"     "poltrst2"
## [24,] "cntry"     "poleffic"
## [25,] "cntry"     "resource"
## [26,] "cntry"     "sgnptit"
## [27,] "cntry"     "polintr"
## [28,] "cntry"     "hincfel"
## [29,] "cntry"     "pdwrk"
## [30,] "cntry"     "wrkorg"
## [31,] "cntry"     "rlgblg"
## [32,] "cntry"     "dscrgrp"
## [33,] "cntry"     "mbtru"
## [34,] "cntry"     "eduyrs100"
## [35,] "cntry"     "lrscale"
```

Comparing all of the structures, several uncertainties in relation to the relationships between the variables were distinguished. Thus, the following causal relations are questioned.

1. Direct or reverse causation between political and social trust.
2. Direct or reverse causation between education and internal political efficacy.
3. Direct or reverse causation between external and internal political efficacy.
4. Direct or reverse causation between participation in online activism and working in an NGO.
5. Direct causation between political interest and working in an NGO.
6. Direct causation between gender and internal political efficacy.
7. Direct causation between political trust and self-identification with a discriminated group.
8. Direct causation between internal political efficacy and self-identification with a discriminated group.
9. Direct causation between internal political efficacy and party identification.
10. Direct causation between participation in online activism and self-identification with a discriminated group.

In order to test for the significance of the paths, structural equation modeling was conducted.

Structural equation modeling

To test for the significance of the learned paths and choose the most reliable structure, structural equation modeling was applied. Lavaan package (Rosseel et al. 2020) was used to analyse the data.

The first structure estimated within structural equation modeling includes only those arcs that were distinguished by both algorithms, TABU and H2PC.

```
# Here, we have to use ESS9Dat2 dataset as it contains variables that
# were not discretized
# Subseting the dataset to exclude variables received by applying
# factor analysis
ESS9Dat2<-ESS9Dat2[,1:31]
# Scaling the variables
for (i in 2:31){
  ESS9Dat2[,i]<-scale(ESS9Dat2[,i])
```

```
}

# Looking at the arcs present in both structures, the one learned by
# applying TABU and the one learned by applying H2PC
compare(avg.diff,avg.diff2, arcs=T)$tp
```

```
##       from        to
##  [1,] "poltrst2"  "poleffic"
##  [2,] "poltrst2"  "clsprty"
##  [3,] "resource"  "pstplonl"
##  [4,] "resource"  "polintr"
##  [5,] "resource"  "wrkorg"
##  [6,] "pstplonl"  "clsprty"
##  [7,] "pstplonl"  "dscrgrp"
##  [8,] "polintr"   "pstplonl"
##  [9,] "polintr"   "clsprty"
## [10,] "clsprty"   "lrscale"
## [11,] "pdwrk"     "eduyrs100"
## [12,] "wrkorg"    "pstplonl"
## [13,] "brncntr"   "dscrgrp"
## [14,] "agea100"   "pstplonl"
## [15,] "agea100"   "polintr"
## [16,] "agea100"   "clsprty"
## [17,] "agea100"   "pdwrk"
## [18,] "agea100"   "rlgblg"
## [19,] "agea100"   "mbtru"
## [20,] "eduyrs100" "hincfel"
## [21,] "cntry"     "soctrst"
## [22,] "cntry"     "poltrst2"
## [23,] "cntry"     "poleffic"
## [24,] "cntry"     "resource"
## [25,] "cntry"     "polintr"
## [26,] "cntry"     "hincfel"
## [27,] "cntry"     "pdwrk"
## [28,] "cntry"     "wrkorg"
## [29,] "cntry"     "rlgblg"
## [30,] "cntry"     "mbtru"
## [31,] "cntry"     "eduyrs100"
## [32,] "cntry"     "lrscale"
```

```
library(lavaan)

# The model excludes arcs between participation and work in a NGO
# and participation and identification with a discriminated group as
# those arcs were questioned after performing the robustness test
mod1 <- "
    poltrst2 =~ trstplt+trstprt+trstprl+trstlgl+trstplc
    soctrst =~ ppltrst+pplfair+pplhlp
    poleffic =~ psppsgva+psppipla
    resource=~actrolga+cptppola

  poleffic~poltrst2
  polintr~resource+agea100
  pdwrk~agea100
```

```
  wrkorg~resource
  rlgblg~agea100
  mbtru~agea100
  pstplonl~resource+polintr+agea100
  eduyrs100~pdwrk
  clsprty~poltrst2+pstplonl+polintr+agea100
  hincfel~eduyrs100
  dscrgrp~brncntr
  lrscale~clsprty
"
# Estimating the model
m1 <- sem(mod1, data=ESS9Dat2)
```

The next step was to add questioned arcs one by one and test for the significance of the paths. The structure with the best model fit was retained on each of the steps (the chi-square tests were computed to compare the models).

```
# Checking if adding a link between internal political efficacy and
# gender improves the model (by computing the chi-square tests to compare
# mod1 and the new model)
# Creating a new model "mod2"
mod2 <- "
    poltrst2 =~ trstplt+trstprt+trstprl+trstlgl+trstplc
    soctrst =~ ppltrst+pplfair+pplhlp
    poleffic =~ psppsgva+psppipla
    resource=~actrolga+cptppola

  poleffic~poltrst2
  polintr~resource+agea100
  pdwrk~agea100
  wrkorg~resource
  rlgblg~agea100
  mbtru~agea100
  pstplonl~resource+polintr+agea100
  eduyrs100~pdwrk
  clsprty~poltrst2+pstplonl+polintr+agea100
  hincfel~eduyrs100
  dscrgrp~brncntr
  lrscale~clsprty
  resource~gndr
"
m2 <- sem(mod2, data=ESS9Dat2)
```

```
anova(m1,m2) # comparing the models m1 and m2
```

```
## Chi-Squared Difference Test
##
##      Df     AIC     BIC Chisq Chisq diff Df diff
## m1 245 1613099 1613732 27486
## m2 269 1613987 1614612 29910     2423.8      24
##    Pr(>Chisq)
## m1
## m2  < 2.2e-16 ***
## ---
```

```
## Signif. codes:
## 0 '***' 0.001 '**' 0.01 '*' 0.05 '.' 0.1 ' ' 1
```

```
# Gender does not seem to play a "significant" role
```

```
# Checking if adding a link from education to internal political efficacy
# improves the model (by computing the chi-square tests to compare
# mod1 and the new model)
# Creating a new model "mod3"
mod3 <- "
    poltrst2 =~ trstplt+trstprt+trstprl+trstlgl+trstplc
    soctrst =~ ppltrst+pplfair+pplhlp
    poleffic =~ psppsgva+psppipla
    resource=~actrolga+cptppola

  poleffic~poltrst2
  polintr~resource+agea100
  pdwrk~agea100
  wrkorg~resource
  rlgblg~agea100
  mbtru~agea100
  pstplonl~resource+polintr+agea100
  eduyrs100~pdwrk
  clsprty~poltrst2+pstplonl+polintr+agea100
  hincfel~eduyrs100
  dscrgrp~brncntr
  lrscale~clsprty
  resource~eduyrs100
"
m3 <- sem(mod3, data=ESS9Dat2)

# Checking if adding a link from internal political efficacy to education
# improves the model (by computing the chi-square tests to compare
# mod1 and the new model)
# Creating a new model "mod4"
mod4 <- "
    poltrst2 =~ trstplt+trstprt+trstprl+trstlgl+trstplc
    soctrst =~ ppltrst+pplfair+pplhlp
    poleffic =~ psppsgva+psppipla
    resource=~actrolga+cptppola

  poleffic~poltrst2
  polintr~resource+agea100
  pdwrk~agea100
  wrkorg~resource
  rlgblg~agea100
  mbtru~agea100
  pstplonl~resource+polintr+agea100
  eduyrs100~pdwrk+resource
  clsprty~poltrst2+pstplonl+polintr+agea100
  hincfel~eduyrs100
  dscrgrp~brncntr
  lrscale~clsprty
"
```

```r
m4 <- sem(mod4, data=ESS9Dat2)
```

```r
#Comparing three models
anova(m1,m3)
```

```
## Chi-Squared Difference Test
##
##      Df     AIC     BIC Chisq Chisq diff Df diff
## m1 245 1613099 1613732 27486
## m3 246 1612455 1613079 26843     -642.8       1
##    Pr(>Chisq)
## m1
## m3          1
```

```r
anova(m1,m4)
```

```
## Chi-Squared Difference Test
##
##      Df     AIC     BIC Chisq Chisq diff Df diff
## m4 244 1611103 1611744 25488
## m1 245 1613099 1613732 27486     1998.1       1
##    Pr(>Chisq)
## m4
## m1  < 2.2e-16 ***
## ---
## Signif. codes:
## 0 '***' 0.001 '**' 0.01 '*' 0.05 '.' 0.1 ' ' 1
```

```r
anova(m3,m4)
```

```
## Chi-Squared Difference Test
##
##      Df     AIC     BIC Chisq Chisq diff Df diff
## m4 244 1611103 1611744 25488
## m3 246 1612455 1613079 26843     1355.3       2
##    Pr(>Chisq)
## m4
## m3  < 2.2e-16 ***
## ---
## Signif. codes:
## 0 '***' 0.001 '**' 0.01 '*' 0.05 '.' 0.1 ' ' 1
```

```r
# Model 3 has a better fit
```

```r
# Checking if adding a link from internal political efficacy to
# external political efficacy improves the model (by computing the
# chi-square tests to compare mod3 and the new model)
# Creating a new model "mod5"
mod5 <- "
    poltrst2 =~ trstplt+trstprt+trstprl+trstlgl+trstplc
    soctrst =~ ppltrst+pplfair+pplhlp
```

```
    poleffic =~ psppsgva+psppipla
    resource=~actrolga+cptppola

  poleffic~poltrst2 + resource
  polintr~resource+agea100
  pdwrk~agea100
  wrkorg~resource
  rlgblg~agea100
  mbtru~agea100
  pstplonl~resource+polintr+agea100
  eduyrs100~pdwrk+resource
  clsprty~poltrst2+pstplonl+polintr+agea100
  hincfel~eduyrs100
  dscrgrp~brncntr
  lrscale~clsprty
"
m5 <- sem(mod5, data=ESS9Dat2)

# Checking if adding a link from external political efficacy to
# internal political efficacy improves the model (by computing the
# chi-square tests to compare mod3 and the new model)
# Creating a new model "mod6"
mod6 <- "
    poltrst2 =~ trstplt+trstprt+trstprl+trstlgl+trstplc
    soctrst =~ ppltrst+pplfair+pplhlp
    poleffic =~ psppsgva+psppipla
    resource=~actrolga+cptppola

  poleffic~poltrst2
  polintr~resource+agea100
  pdwrk~agea100
  wrkorg~resource
  rlgblg~agea100
  mbtru~agea100
  pstplonl~resource+polintr+agea100
  eduyrs100~pdwrk+resource
  clsprty~poltrst2+pstplonl+polintr+agea100
  hincfel~eduyrs100
  dscrgrp~brncntr
  lrscale~clsprty
  resource~poleffic
"
m6 <- sem(mod6, data=ESS9Dat2)
```

```
#Comparing three models
anova(m3,m5)
```

```
## Chi-Squared Difference Test
##
##      Df     AIC     BIC Chisq Chisq diff Df diff
## m5 243 1608645 1609294 23027
## m3 246 1612455 1613079 26843     3816.1       3
##    Pr(>Chisq)
## m5
```

```
## m3  < 2.2e-16 ***
## ---
## Signif. codes:
## 0 '***' 0.001 '**' 0.01 '*' 0.05 '.' 0.1 ' ' 1
```

```
anova(m3,m6)
```

```
## Chi-Squared Difference Test
##
##      Df     AIC     BIC Chisq Chisq diff Df diff
## m3 246 1612455 1613079 26843
## m6 251 1609103 1609686 23502    -3341.8       5
##    Pr(>Chisq)
## m3
## m6          1
```

```
anova(m5,m6)
```

```
## Chi-Squared Difference Test
##
##      Df     AIC     BIC Chisq Chisq diff Df diff
## m5 243 1608645 1609294 23027
## m6 251 1609103 1609686 23502     474.37       8
##    Pr(>Chisq)
## m5
## m6  < 2.2e-16 ***
## ---
## Signif. codes:
## 0 '***' 0.001 '**' 0.01 '*' 0.05 '.' 0.1 ' ' 1
```

```
# Model 5 has a better fit
```

```
# Checking if adding a link from internal political efficacy to
# identification with a discriminated group improves the model (by
# computing the chi-square tests to compare mod5 and the new model)
# Creating a new model "mod7"
mod7 <- "
    poltrst2 =~ trstplt+trstprt+trstprl+trstlgl+trstplc
    soctrst =~ ppltrst+pplfair+pplhlp
    poleffic =~ psppsgva+psppipla
    resource=~actrolga+cptppola

  poleffic~poltrst2 + resource
  polintr~resource+agea100
  pdwrk~agea100
  wrkorg~resource
  rlgblg~agea100
  mbtru~agea100
  pstplonl~resource+polintr+agea100
  eduyrs100~pdwrk+resource
  clsprty~poltrst2+pstplonl+polintr+agea100
  hincfel~eduyrs100
```

```
  dscrgrp~brncntr+resource
  lrscale~clsprty
"
m7 <- sem(mod7, data=ESS9Dat2)
```

```
#Comparing two models
anova(m5,m7)
```

```
## Chi-Squared Difference Test
##
##      Df     AIC     BIC Chisq Chisq diff Df diff
## m7 242 1608496 1609154 22877
## m5 243 1608645 1609294 23027     150.33       1
##    Pr(>Chisq)
## m7
## m5  < 2.2e-16 ***
## ---
## Signif. codes:
## 0 '***' 0.001 '**' 0.01 '*' 0.05 '.' 0.1 ' ' 1
```

```
# Model 7 has a better fit
```

```
# Checking if adding a link from internal political efficacy to
# party identification improves the model (by computing
# the chi-square tests to compare mod7 and the new model)
# Creating a new model "mod8"
mod8 <- "
    poltrst2 =~ trstplt+trstprt+trstprl+trstlgl+trstplc
    soctrst =~ ppltrst+pplfair+pplhlp
    poleffic =~ psppsgva+psppipla
    resource=~actrolga+cptppola

  poleffic~poltrst2 + resource
  polintr~resource+agea100
  pdwrk~agea100
  wrkorg~resource
  rlgblg~agea100
  mbtru~agea100
  pstplonl~resource+polintr+agea100
  eduyrs100~pdwrk+resource
  clsprty~poltrst2+pstplonl+polintr+agea100+resource
  hincfel~eduyrs100
  dscrgrp~brncntr+resource
  lrscale~clsprty
"
m8 <- sem(mod8, data=ESS9Dat2)
```

```
#Comparing two models
anova(m7,m8)
```

```
## Chi-Squared Difference Test
##
```

```
##      Df     AIC     BIC Chisq Chisq diff Df diff
## m8 241 1608339 1609004 22717
## m7 242 1608496 1609154 22877     159.56       1
##    Pr(>Chisq)
## m8
## m7  < 2.2e-16 ***
## ---
## Signif. codes:
## 0 '***' 0.001 '**' 0.01 '*' 0.05 '.' 0.1 ' ' 1
```

```
# Model 8 has a better fit
```

```
# Checking if adding a link from political trust to
# social trust improves the model (by computing
# the chi-square tests to compare mod8 and the new model)
# Creating a new model "mod9"
mod9 <- "
    poltrst2 =~ trstplt+trstprt+trstprl+trstlgl+trstplc
    soctrst =~ ppltrst+pplfair+pplhlp
    poleffic =~ psppsgva+psppipla
    resource=~actrolga+cptppola

  poleffic~poltrst2 + resource
  polintr~resource+agea100
  pdwrk~agea100
  wrkorg~resource
  rlgblg~agea100
  mbtru~agea100
  pstplonl~resource+polintr+agea100
  eduyrs100~pdwrk+resource
  clsprty~poltrst2+pstplonl+polintr+agea100+resource
  hincfel~eduyrs100
  dscrgrp~brncntr+resource
  lrscale~clsprty
  soctrst~poltrst2
"
m9 <- sem(mod9, data=ESS9Dat2)

# Checking if adding a link from social trust to
# political trust improves the model (by computing
# the chi-square tests to compare mod8 and the new model)
# Creating a new model "mod10"
mod10 <- "
    poltrst2 =~ trstplt+trstprt+trstprl+trstlgl+trstplc
    soctrst =~ ppltrst+pplfair+pplhlp
    poleffic =~ psppsgva+psppipla
    resource=~actrolga+cptppola

  poleffic~poltrst2 + resource
  polintr~resource+agea100
  pdwrk~agea100
  wrkorg~resource
  rlgblg~agea100
  mbtru~agea100
```

```
  pstplonl~resource+polintr+agea100
  eduyrs100~pdwrk+resource
  clsprty~poltrst2+pstplonl+polintr+agea100+resource
  hincfel~eduyrs100
  dscrgrp~brncntr+resource
  lrscale~clsprty
  poltrst2~soctrst
"
m10 <- sem(mod10, data=ESS9Dat2)
```

```
#Comparing three models
anova(m8,m9)
```

```
## Chi-Squared Difference Test
##
##     Df     AIC     BIC Chisq Chisq diff Df diff
## m9 235 1607719 1608434 22086
## m8 241 1608339 1609004 22717     631.45       6
##    Pr(>Chisq)
## m9
## m8  < 2.2e-16 ***
## ---
## Signif. codes:
## 0 '***' 0.001 '**' 0.01 '*' 0.05 '.' 0.1 ' ' 1
```

```
anova(m8,m10)
```

```
## Chi-Squared Difference Test
##
##      Df     AIC     BIC Chisq Chisq diff Df diff
## m8  241 1608339 1609004 22717
## m10 242 1608737 1609394 23118     400.29       1
##     Pr(>Chisq)
## m8
## m10  < 2.2e-16 ***
## ---
## Signif. codes:
## 0 '***' 0.001 '**' 0.01 '*' 0.05 '.' 0.1 ' ' 1
```

```
anova(m9,m10)
```

```
## Chi-Squared Difference Test
##
##      Df     AIC     BIC Chisq Chisq diff Df diff
## m9  235 1607719 1608434 22086
## m10 242 1608737 1609394 23118     1031.8       7
##     Pr(>Chisq)
## m9
## m10  < 2.2e-16 ***
## ---
## Signif. codes:
## 0 '***' 0.001 '**' 0.01 '*' 0.05 '.' 0.1 ' ' 1
```

```
# Model 9 has a better fit
```

```
# Checking if adding a link from political trust to
# identification with a discriminated group improves the model (by
# computing the chi-square tests to compare mod9 and the new model)
# Creating a new model "mod11"
mod11 <- "
    poltrst2 =~ trstplt+trstprt+trstprl+trstlgl+trstplc
    soctrst =~ ppltrst+pplfair+pplhlp
    poleffic =~ psppsgva+psppipla
    resource=~actrolga+cptppola

  poleffic~poltrst2 + resource
  polintr~resource+agea100
  pdwrk~agea100
  wrkorg~resource
  rlgblg~agea100
  mbtru~agea100
  pstplonl~resource+polintr+agea100
  eduyrs100~pdwrk+resource
  clsprty~poltrst2+pstplonl+polintr+agea100+resource
  hincfel~eduyrs100
  dscrgrp~brncntr+resource+poltrst2
  lrscale~clsprty
  soctrst~poltrst2
"
m11 <- sem(mod11, data=ESS9Dat2)
```

```
#Comparing two models
anova(m9,m11)
```

```
## Chi-Squared Difference Test
##
##       Df     AIC     BIC Chisq Chisq diff Df diff
## m11 234 1607373 1608096 21738
## m9  235 1607719 1608434 22086     348.27       1
##      Pr(>Chisq)
## m11
## m9   < 2.2e-16 ***
## ---
## Signif. codes:
## 0 '***' 0.001 '**' 0.01 '*' 0.05 '.' 0.1 ' ' 1
```

```
# Model 11 has a better fit
```

```
# Checking if adding a link from online participation to
# identification with a discriminated group improves the model (by
# computing the chi-square tests to compare mod11 and the new model)
# Creating a new model "mod12"
mod12 <- "
    poltrst2 =~ trstplt+trstprt+trstprl+trstlgl+trstplc
    soctrst =~ ppltrst+pplfair+pplhlp
```

```
    poleffic =~ psppsgva+psppipla
    resource=~actrolga+cptppola

  poleffic~poltrst2 + resource
  polintr~resource+agea100
  pdwrk~agea100
  wrkorg~resource
  rlgblg~agea100
  mbtru~agea100
  pstplonl~resource+polintr+agea100
  eduyrs100~pdwrk+resource
  clsprty~poltrst2+pstplonl+polintr+agea100+resource
  hincfel~eduyrs100
  dscrgrp~brncntr+resource+poltrst2+pstplonl
  lrscale~clsprty
  soctrst~poltrst2
"
m12 <- sem(mod12, data=ESS9Dat2)
```

```
#Comparing two models
anova(m11,m12)
```

```
## Chi-Squared Difference Test
##
##      Df     AIC     BIC Chisq Chisq diff Df diff
## m12 233 1607129 1607860 21492
## m11 234 1607373 1608096 21738     246.03       1
##     Pr(>Chisq)
## m12
## m11  < 2.2e-16 ***
## ---
## Signif. codes:
## 0 '***' 0.001 '**' 0.01 '*' 0.05 '.' 0.1 ' ' 1
```

```
# Model 12 has a better fit
```

```
# Checking if adding a link from political interest to
# working in an NGO improves the model (by computing
# the chi-square tests to compare mod12 and the new model)
# Creating a new model "mod13"
mod13 <- "
    poltrst2 =~ trstplt+trstprt+trstprl+trstlgl+trstplc
    soctrst =~ ppltrst+pplfair+pplhlp
    poleffic =~ psppsgva+psppipla
    resource=~actrolga+cptppola

  poleffic~poltrst2 + resource
  polintr~resource+agea100
  pdwrk~agea100
  wrkorg~resource + polintr
  rlgblg~agea100
  mbtru~agea100
  pstplonl~resource+polintr+agea100
```

```
  eduyrs100~pdwrk+resource
  clsprty~poltrst2+pstplonl+polintr+agea100+resource
  hincfel~eduyrs100
  dscrgrp~brncntr+resource+poltrst2+pstplonl
  lrscale~clsprty
  soctrst~poltrst2
"
m13 <- sem(mod13, data=ESS9Dat2)
```

```
#Comparing two models
anova(m12,m13)
```

```
## Chi-Squared Difference Test
##
##       Df      AIC     BIC Chisq Chisq diff Df diff
## m13 232 1607053 1607793 21414
## m12 233 1607129 1607860 21492     77.494       1
##     Pr(>Chisq)
## m13
## m12  < 2.2e-16 ***
## ---
## Signif. codes:
## 0 '***' 0.001 '**' 0.01 '*' 0.05 '.' 0.1 ' ' 1
```

```
# Model 13 has a better fit
```

```
# Checking if adding a link from online participation to
# working in an NGO improves the model (by computing
# the chi-square tests to compare mod13 and the new model)
# Creating a new model "mod14"
mod14 <- "
    poltrst2 =~ trstplt+trstprt+trstprl+trstlgl+trstplc
    soctrst =~ ppltrst+pplfair+pplhlp
    poleffic =~ psppsgva+psppipla
    resource=~actrolga+cptppola

  poleffic~poltrst2 + resource
  polintr~resource+agea100
  pdwrk~agea100
  wrkorg~resource + polintr+pstplonl
  rlgblg~agea100
  mbtru~agea100
  pstplonl~resource+polintr+agea100
  eduyrs100~pdwrk+resource
  clsprty~poltrst2+pstplonl+polintr+agea100+resource
  hincfel~eduyrs100
  dscrgrp~brncntr+resource+poltrst2+pstplonl
  lrscale~clsprty
  soctrst~poltrst2
"
m14 <- sem(mod14, data=ESS9Dat2)

# Checking if adding a link from working in an NGO to
```

```
# online participation improves the model (by computing
# the chi-square tests to compare mod13 and the new model)
# Creating a new model "mod15"
mod15 <- "
    poltrst2 =~ trstplt+trstprt+trstprl+trstlgl+trstplc
    soctrst =~ ppltrst+pplfair+pplhlp
    poleffic =~ psppsgva+psppipla
    resource=~actrolga+cptppola

  poleffic~poltrst2 + resource
  polintr~resource+agea100
  pdwrk~agea100
  wrkorg~resource + polintr
  rlgblg~agea100
  mbtru~agea100
  pstplonl~resource+polintr+agea100  +wrkorg
  eduyrs100~pdwrk+resource
  clsprty~poltrst2+pstplonl+polintr+agea100+resource
  hincfel~eduyrs100
  dscrgrp~brncntr+resource+poltrst2+pstplonl
  lrscale~clsprty
  soctrst~poltrst2
"
m15 <- sem(mod15, data=ESS9Dat2)
```

```
#Comparing three models
anova(m13,m14)
```

```
## Chi-Squared Difference Test
##
##       Df     AIC     BIC Chisq Chisq diff Df diff
## m14 231 1606888 1607636 21247
## m13 232 1607053 1607793 21414     167.07       1
##     Pr(>Chisq)
## m14
## m13  < 2.2e-16 ***
## ---
## Signif. codes:
## 0 '***' 0.001 '**' 0.01 '*' 0.05 '.' 0.1 ' ' 1
```

```
anova(m13,m15)
```

```
## Chi-Squared Difference Test
##
##       Df     AIC     BIC Chisq Chisq diff Df diff
## m13 232 1607053 1607793 21414
## m15 238 1607395 1608086 21768     353.94       6
##     Pr(>Chisq)
## m13
## m15  < 2.2e-16 ***
## ---
## Signif. codes:
## 0 '***' 0.001 '**' 0.01 '*' 0.05 '.' 0.1 ' ' 1
```

```
anova(m14,m15)
```

```
## Chi-Squared Difference Test
##
##       Df     AIC     BIC Chisq Chisq diff Df diff
## m14 231 1606888 1607636 21247
## m15 238 1607395 1608086 21768     521.01       7
##     Pr(>Chisq)
## m14
## m15  < 2.2e-16 ***
## ---
## Signif. codes:
## 0 '***' 0.001 '**' 0.01 '*' 0.05 '.' 0.1 ' ' 1
```

```
# Model 14 has a better fit
```

```
# Plotting the model with the best fit
library(lavaanPlot) # using the package to plot the results
lavaanPlot(model=m14, coefs=T, edge_options=list(fontsize=14, color = "
   grey"),
           node_options=list(fontsize=16), stars="regress")
```

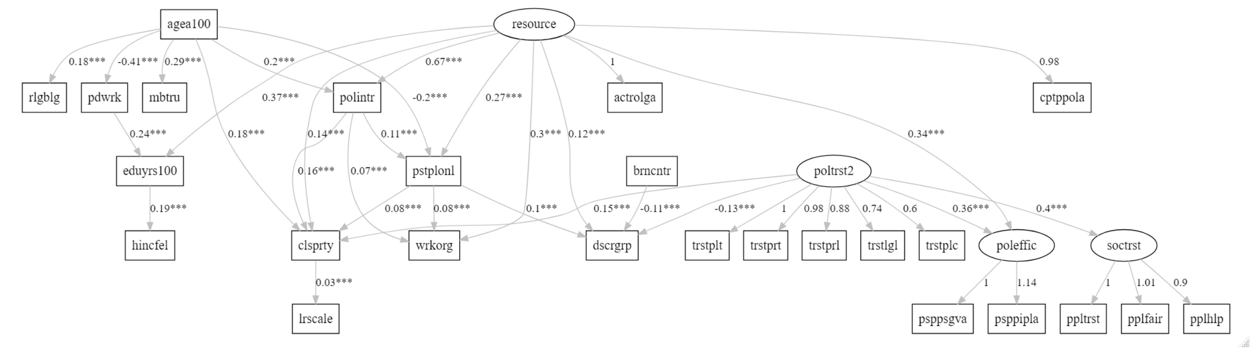

The structure with the best model fit was chosen for acquiring the set of conditional probability distribution tables.

Exacting inference

Once, the structure with the best model fit was determined, a new empty graph was created using bnlearn tools (Scutari and Ness 2019). The list of arcs was set to those directed edges that were determined on the previous step.

The code shows how a new bnlearn graph was created and the arcs were set.

```
# The new graph will contain the arcs (learned by TABU and H2PC) from
# "Country" to other nodes

# Checking if the both of the algorithms agree on those arcs
b<-as.data.frame(arcs(avg.diff)) # saving the arcs of the structure
# received by applying TABU
b1<-as.data.frame(arcs(avg.diff2)) # saving the arcs of the structure
```

```
# received by applying H2PC

# Comparing the arcs from "Country" to other nodes
b<-b[b$from=="cntry",]
b1<-b1[b1$from=="cntry",]
```

```
for (i in 1:nrow(b1)){
cat(b$from[i],"->",b$to[i],"---",b1$from[i],"->",b1$to[i],"\n")
}
```

```
## cntry -> soctrst --- cntry -> soctrst
## cntry -> poltrst2 --- cntry -> poltrst2
## cntry -> poleffic --- cntry -> poleffic
## cntry -> resource --- cntry -> resource
## cntry -> polintr --- cntry -> polintr
## cntry -> hincfel --- cntry -> hincfel
## cntry -> pdwrk --- cntry -> pdwrk
## cntry -> wrkorg --- cntry -> wrkorg
## cntry -> rlgblg --- cntry -> rlgblg
## cntry -> mbtru --- cntry -> mbtru
## cntry -> eduyrs100 --- cntry -> eduyrs100
## cntry -> lrscale --- cntry -> lrscale
```

```
# The algorithms agree on the influence of "Country"

# Using the same arcs for the new graph
newdag <- empty.graph(nodes = names(d)) # creating an empty DAG
arcs(newdag) <- b # loading the arcs from "Country" to other nodes
                  # to the new DAG

# Setting other arcs that were received by comparing numerous models

# Setting the arcs defined by the model 14 (mod14 or m14)
newdag <- set.arc(newdag, from = "agea100", to = "mbtru")
newdag <- set.arc(newdag, from = "agea100", to = "rlgblg")
newdag <- set.arc(newdag, from = "agea100", to = "pstplonl")
newdag <- set.arc(newdag, from = "agea100", to = "pdwrk")
newdag <- set.arc(newdag, from = "agea100", to = "clsprty")
newdag <- set.arc(newdag, from = "agea100", to = "polintr")

newdag <- set.arc(newdag, from = "resource", to = "eduyrs100")
newdag <- set.arc(newdag, from = "eduyrs100", to = "hincfel")
newdag <- set.arc(newdag, from = "pdwrk", to = "eduyrs100")

newdag <- set.arc(newdag, from = "resource", to = "poleffic")
newdag <- set.arc(newdag, from = "resource", to = "polintr")
newdag <- set.arc(newdag, from = "resource", to = "wrkorg")
newdag <- set.arc(newdag, from = "resource", to = "pstplonl")
newdag <- set.arc(newdag, from = "resource", to = "clsprty")
newdag <- set.arc(newdag, from = "resource", to = "dscrgrp")

newdag <- set.arc(newdag, from = "polintr", to = "wrkorg")
newdag <- set.arc(newdag, from = "polintr", to = "pstplonl")
```

```r
newdag <- set.arc(newdag, from = "polintr", to = "clsprty")

newdag <- set.arc(newdag, from = "pstplonl", to = "wrkorg")
newdag <- set.arc(newdag, from = "pstplonl", to = "clsprty")
newdag <- set.arc(newdag, from = "pstplonl", to = "dscrgrp")

newdag <- set.arc(newdag, from = "brncntr", to = "dscrgrp")

newdag <- set.arc(newdag, from = "poltrst2", to = "soctrst")
newdag <- set.arc(newdag, from = "poltrst2", to = "poleffic")
newdag <- set.arc(newdag, from = "poltrst2", to = "clsprty")
newdag <- set.arc(newdag, from = "poltrst2", to = "dscrgrp")

newdag <- set.arc(newdag, from = "clsprty", to = "lrscale")
```

A network consists of 39 directed edges. After the new DAG was created, it was transformed into a junction tree (using gRain tools (Højsgaard 2020)) and its probability tables were computed.

```r
# Fitting the parameters of the Bayesian network conditional on the new
# structure
# Using Bayesian parameter estimation as the method
bn.bayes <- bn.fit(newdag, data = d, method = "bayes")

# Using gRain package for exacting inference
library(gRain)

# Computing the probability tables of the junction tree
junction <- compile(as.grain(bn.bayes))
```

```r
# Examining the probability tables
querygrain(junction)
```

```
## $soctrst
## soctrst
##         0         1
## 0.4186085 0.5813915
##
## $poltrst2
## poltrst2
##         0         1
## 0.4269357 0.5730643
##
## $cntry
## cntry
##          AT          BE          BG          CH
## 0.07396102 0.05916920 0.03188651 0.04408519
##          CY          CZ          DE          EE
## 0.01892084 0.06610857 0.07925685 0.06055707
##          FI          FR          GB          HU
## 0.05920572 0.06099535 0.06800777 0.04514436
##          IE          IT          NL          NO
## 0.06198147 0.06044750 0.05343509 0.04536350
##          PL          RS          SI
## 0.03656145 0.03922763 0.03568490
```

```
##
## $poleffic
## poleffic
##         0         1
## 0.4852851 0.5147149
##
## $resource
## resource
##         0         1
## 0.5432615 0.4567385
##
## $pstplonl
## pstplonl
##         0         1
## 0.8297014 0.1702986
##
## $polintr
## polintr
##         0         1
## 0.5098189 0.4901811
##
## $agea100
## agea100
##         1         2         3         4
## 0.1649470 0.2260500 0.2675402 0.3414627
##
## $clsprty
## clsprty
##         0         1
## 0.4828257 0.5171743
##
## $wrkorg
## wrkorg
##         0         1
## 0.8240816 0.1759184
##
## $pdwrk
## pdwrk
##         0         1
## 0.4451335 0.5548665
##
## $eduyrs100
## eduyrs100
##          1          2          3          4
## 0.01931507 0.17262066 0.52218030 0.28588397
##
## $dscrgrp
## dscrgrp
##          0          1
## 0.92756696 0.07243304
##
## $brncntr
## brncntr
##          0          1
```

```
## 0.09158145 0.90841855
##
## $hincfel
## hincfel
##         0         1
## 0.6562946 0.3437054
##
## $rlgblg
## rlgblg
##         0         1
## 0.4467858 0.5532142
##
## $mbtru
## mbtru
##         0         1
## 0.6060667 0.3939333
##
## $lrscale
## lrscale
##         1         2         3
## 0.3164863 0.3303400 0.3531736
##
## $gndr
## gndr
##         0         1
## 0.5101351 0.4898649
```

```
# Examining the probability distribution of "Participation in online
# activism"
querygrain(junction, nodes = "pstplonl")
```

```
## $pstplonl
## pstplonl
##         0         1
## 0.8297014 0.1702986
```

Using the received probability distribution tables, it is possible to construct conditional probability queries, i.e., calculate the probabilities corresponding to an event under specific conditions(Scutari and Denis 2014). For example, we can set the following conditions and see how the probability of participation in online activism changes.

1. The probability of a person to participate in online activism if this person is an individual with the low income, graduate level of education, working in a non-governmental organization, placing oneself on the left of the left-right scale and having a low political trust.

```
# Examining the probability tables having prior knowledge
evid1 <- setEvidence(junction, nodes = "hincfel", states = "0")
```

```
querygrain(evid1, nodes = "pstplonl")# see the probability distribution
```

```
## $pstplonl
## pstplonl
```

```
##          0          1
## 0.8374708 0.1625292
```

```
evid1 <- setEvidence(evid1, nodes = "eduyrs100", states = "4")
```

```
querygrain(evid1, nodes = "pstplonl")
```

```
## $pstplonl
## pstplonl
##          0          1
## 0.8039997 0.1960003
```

```
evid1 <- setEvidence(evid1, nodes = "wrkorg", states = "1")
```

```
querygrain(evid1, nodes = "pstplonl")
```

```
## $pstplonl
## pstplonl
##          0          1
## 0.6773301 0.3226699
```

```
evid1 <- setEvidence(evid1, nodes = "lrscale", states = "0")
```

```
querygrain(evid1, nodes = "pstplonl")
```

```
## $pstplonl
## pstplonl
##          0          1
## 0.6773301 0.3226699
```

```
evid1 <- setEvidence(evid1, nodes = "poltrst2", states = "0")
```

```
querygrain(evid1, nodes = "pstplonl")
```

```
## $pstplonl
## pstplonl
##          0          1
## 0.6534094 0.3465906
```

2. The probability to participate in online activism of a 31-45 y.o. employed person who was born in the country of residence and has high levels of political trust, internal political efficacy, political interest and social trust.

```
evid2 <- setEvidence(junction, nodes = "agea100", states = "2")
```

```
querygrain(evid2, nodes = "pstplonl")
```

```
## $pstplonl
## pstplonl
##         0         1
## 0.7699497 0.2300503
```

```
evid2 <- setEvidence(evid2, nodes = "pdwrk", states = "1")
```

```
querygrain(evid2, nodes = "pstplonl")
```

```
## $pstplonl
## pstplonl
##         0         1
## 0.7697857 0.2302143
```

```
evid2 <- setEvidence(evid2, nodes = "brncntr", states = "1")
```

```
querygrain(evid2, nodes = "pstplonl")
```

```
## $pstplonl
## pstplonl
##         0         1
## 0.7697857 0.2302143
```

```
evid2 <- setEvidence(evid2, nodes = "poltrst2", states = "1")
querygrain(evid2, nodes = "pstplonl")
```

```
## $pstplonl
## pstplonl
##        0        1
## 0.763632 0.236368
```

```
evid2 <- setEvidence(evid2, nodes = "resource", states = "1")
```

```
querygrain(evid2, nodes = "pstplonl")
```

```
## $pstplonl
## pstplonl
##         0         1
## 0.6738875 0.3261125
```

```
evid2 <- setEvidence(evid2, nodes = "polintr", states = "1")
```

```
querygrain(evid2, nodes = "pstplonl")
```

```
## $pstplonl
## pstplonl
##         0         1
## 0.6159456 0.3840544
```

```r
evid2 <- setEvidence(evid2, nodes = "polintr", states = "1")
```

```r
querygrain(evid2, nodes = "soctrst")
```

```
## $soctrst
## soctrst
##         0         1
## 0.2435831 0.7564169
```

References

Gasse, Maxime, Alex Aussem, and Haytham Elghazel. 2014. "A Hybrid Algorithm for Bayesian Network Structure Learning with Application to Multi-Label Learning." *Expert Systems with Applications* 41 (15): 6755–72.

Glover, Fred. 1989. "Tabu Search—Part I." *ORSA Journal on Computing* 1 (3): 190–206.

Højsgaard, Søren. 2020. "Package 'gRain': Graphical Independence Networks." *The Comprehensive R Archive Network*.

Iannone, Richard. 2020. *DiagrammeR: Graph/Network Visualization.* https://CRAN.R-project.org/package=DiagrammeR.

Marien, Sofie, Marc Hooghe, and Ellen Quintelier. 2010. "Inequalities in Non-Institutionalised Forms of Political Participation: A Multi-Level Analysis of 25 Countries." Journal Article. *Political Studies* 58: 187–213.

R Core Team. 2020a. *Foreign: Read Data Stored by 'Minitab', 'S', 'Sas', 'Spss', 'Stata', 'Systat', 'Weka', 'dBase', ...* https://CRAN.R-project.org/package=foreign.

———. 2020b. *R: A Language and Environment for Statistical Computing.* Vienna, Austria: R Foundation for Statistical Computing. https://www.R-project.org/.

Revelle, William. 2020. *Psych: Procedures for Psychological, Psychometric, and Personality Research.* Evanston, Illinois: Northwestern University. https://CRAN.R-project.org/package=psych.

Rosseel, Yves, Daniel Oberski, Jarrett Byrnes, Leonard Vanbrabant, Victoria Savalei, Ed Merkle, Michael Hallquist, et al. 2020. "Package 'Lavaan': Latent Variable Analysis." *The Comprehensive R Archive Network*.

Scutari, Marco, and Jean-Baptiste Denis. 2014. *Bayesian Networks: With Examples in R*. Boca Raton, FL: CRC press.

Scutari, Marco, and Robert Ness. 2019. "Package 'Bnlearn': Bayesian Network Structure Learning, Parameter Learning and Inference." *The Comprehensive R Archive Network*.
