# Supplementary material for: Predicting online participation through Bayesian network analysis
Source: PLoS One. 2021 Dec 23;16(12):e0261663. doi: 10.1371/journal.pone.0261663 (PMC8699968; doi:10.1371/journal.pone.0261663)
Supplement: S3 File — The R script used to compare the predictive performance of the models and to test a two-fold approach in network structure learning, i.e., the combination of Bayesian structure learning and structural equation modeling, on simulated data. (PDF) [file pone.0261663.s010.pdf]

Supplementary information for: Predicting online participation through Bayesian network analysis

11/5/2021

Evaluating the predictive performance of the models

Loading the dataset

```
load("Dataset.RData")
```

Applying BN structure learning to the "Online participation dataset"

```
library(tidyverse)
library(bnlearn)

# Creating a blacklist
p<-names(d)
# Variable "Country" cannot be a child
a<-rep("cntry",length(p)-1)
blacklist<-data.frame(p[p!=a[1]],a)

# Variable "Gender" cannot be a child
a<-rep("gndr",length(p)-1)
blacklist<-rbind(blacklist,data.frame(p[p!=a[1]],a))

# Variable "Born in the country" cannot be a child
a<-rep("brncntr",length(p)-1)
blacklist<-rbind(blacklist,data.frame(p[p!=a[1]],a))

# Variable "Age" cannot be a child
a<-rep("agea100",length(p)-1)
blacklist<-rbind(blacklist,data.frame(p[p!=a[1]],a))
names(blacklist)<-c("from","to")
# New object blacklist is created

rm(a,p) #removing the objects from the workspace

# Learning the structure using TABU algorithm
stru_tabu <- tabu(d, blacklist = blacklist)

# Learning the structure using HC algorithm
stru_hc <- hc(d, blacklist = blacklist)
```

```r
# Learning the structure using MMHC algorithm
stru_mmhc <- mmhc(d, blacklist = blacklist)

# Learning the structure using H2PC algorithm
stru_h2pc <- h2pc(d, blacklist = blacklist)

# Model averaging is required to receive more reliable structures
# Applying model averaging to the structure received as the result of using
# TABU algorithm
str.diff_tabu = boot.strength(d, R = 5000, algorithm = "tabu",
                         algorithm.args = list(blacklist = blacklist))
avg.diff_tabu = averaged.network(str.diff_tabu)

# Applying model averaging to the structure received as the result of using
# H2PC algorithm
str.diff_hc = boot.strength(d, R = 5000, algorithm = "hc",
                          algorithm.args = list(blacklist = blacklist))
avg.diff_hc = averaged.network(str.diff_hc)

# Applying model averaging to the structure received as the result of using
# H2PC algorithm
str.diff_mmhc = boot.strength(d, R = 5000, algorithm = "mmhc",
                          algorithm.args = list(blacklist = blacklist))
avg.diff_mmhc = averaged.network(str.diff_mmhc)

# Applying model averaging to the structure received as the result of using
# H2PC algorithm
str.diff_h2pc = boot.strength(d, R = 5000, algorithm = "h2pc",
                          algorithm.args = list(blacklist = blacklist))
avg.diff_h2pc = averaged.network(str.diff_h2pc)
```

Applying SEM for fine-tuning the structures

```r
# Sunsetting the ESS9Dat2 dataset
df<-ESS9Dat2[ , !names(ESS9Dat2) %in% c("poltrst2", "soctrst",
                                        "poleffic" ,"resource",
                                        "contplt","vote","sgnptit")]

library(lavaan)
# Defining the 2-step approach to the structure learning
structure_learning_2_step<-function(df,arcs1,arcs2){

  #uploading the model
  base_arcs<-data.frame(semi_join(as.data.frame(arcs1),
                                  as.data.frame(arcs2)))



  #building the sem model
  build_model<-function(base_arcs){
    mod0<-"poltrst2 =~ trstplt+trstprt+trstprl+trstlgl+trstplc \n
```

```
  soctrst =~ ppltrst+pplfair+pplhlp \n poleffic =~ psppsgva+psppipla \n
  resource=~actrolga+cptppola \n"
  for (i in unique(base_arcs$to)){
    mod0<-paste(mod0,i,"~",
    paste0(base_arcs$from[which(base_arcs$to==i)],collapse ="+"),
    "\n")
  }
  return(mod0)
}
mod0<-build_model(base_arcs)


# evaluating the model
m0 <- lavaan::sem(mod0, data=df)

# choosing only significant paths
base_arcs<-lavaan::standardizedSolution(m0) %>%
  dplyr::filter(!is.na(pvalue)) %>%
  arrange(desc(pvalue)) %>%
  mutate_if("is.numeric","round",3) %>%
  select(-ci.lower,-ci.upper,-z) %>%
  dplyr::filter(pvalue<0.05)%>%
  dplyr::filter(op=="~")%>% select(rhs,lhs)
names(base_arcs)<-c("from","to")

routs_to_check<-anti_join(as.data.frame(arcs1),as.data.frame(arcs2))

base_model<-m0
if (nrow(routs_to_check)!=0){
# finding the best model
repeat{

checked_arcs<-base_arcs #saving those arcs that were checked
# This is important to stop the loop in time

# Building more complex models
m_list<-list()
for (i in 1:nrow(routs_to_check)){
  # building a new model
  assign(paste0("new_arc_set",i),rbind(base_arcs,routs_to_check[i,]))
  assign(paste0("mod",i),build_model(get(paste0("new_arc_set",i))))

  assign(paste0("m",i),lavaan::sem(get(paste0("mod",i)), data=df))
  # estimating the model
  m_list<-append(m_list,get(paste0("m",i)))
}

# Adding paths one by one and comparing the models with ANOVA
for (i in 1:length(m_list)){
  if (slot(m_list[[i]], "optim")$converged==T){
  anova_res<-anova(m_list[[i]],base_model)
  if(is.na(anova_res["m_list[[i]]",]$`Pr(>Chisq)`)&
     (anova_res["m_list[[i]]",]$Chisq<anova_res["base_model",]$Chisq)){
```

```r
      # The models are similar, but a more complex one is still a little
      # bit better
        base_model<-m_list[[i]]
        base_arcs<-get(paste0("new_arc_set",i))
      }else{
        if(!(is.na(anova_res["anova_res",]$`Pr(>Chisq)`))&
           !is.na(anova_res["base_model","Pr(>Chisq)"])&
                  anova_res["base_model","Pr(>Chisq)"]<0.05){
          # A more complex model is significantly better
          base_model<-m_list[[i]]
          base_arcs<-get(paste0("new_arc_set",i))
        }else{
          if(anova_res["m_list[[i]]","AIC"]<anova_res["base_model","AIC"]&
             anova_res["m_list[[i]]","BIC"]<anova_res["base_model","BIC"]){
            # if none of the conditions is true, a more complex model
            # can still substantially increase the predictability
            # So, if both AIC and BIC are smaller than those of the
            # base model, we can still vote for the more complex one.
            # Especially, when it comes to the social-phenomena models
          base_model<-m_list[[i]]
          base_arcs<-get(paste0("new_arc_set",i))
        }
                  }}
      }
  }

  # which arcs still need to be checked
  routs_to_check<-anti_join(routs_to_check,base_arcs)
   # if all of the arcs were checked, stop
   if(identical(checked_arcs,base_arcs)|(nrow(routs_to_check)==0)){
    break
   }
  }}
  # return the best model
  return(base_arcs)
}

# Applying the function to the structures
tabu_h2pc<-structure_learning_2_step(df,arcs(stru_tabu),
                                     arcs(stru_h2pc))
hc_mmhc<-structure_learning_2_step(df,arcs(stru_hc),
                                   arcs(stru_mmhc))
tabu_mmhc<-structure_learning_2_step(df,arcs(stru_tabu),
                                     arcs(stru_mmhc))
hc_h2pc<-structure_learning_2_step(df,arcs(stru_hc),
                                   arcs(stru_h2pc))

avg_tabu_h2pc<-structure_learning_2_step(df,arcs(avg.diff_tabu),
                                         arcs(avg.diff_h2pc))
avg_hc_mmhc<-structure_learning_2_step(df,arcs(avg.diff_hc),
                                       arcs(avg.diff_mmhc))
avg_tabu_mmhc<-structure_learning_2_step(df,arcs(avg.diff_tabu),
                                         arcs(avg.diff_mmhc))
```

```r
avg_hc_h2pc<-structure_learning_2_step(df,arcs(avg.diff_hc),
                                        arcs(avg.diff_h2pc))

# Saving as a BN object
dag_tabu_h2pc <- empty.graph(nodes = names(d))
dag_hc_mmhc <- empty.graph(nodes = names(d))
dag_tabu_mmhc <- empty.graph(nodes = names(d))
dag_hc_h2pc <- empty.graph(nodes = names(d))

dag_avg_tabu_h2pc <- empty.graph(nodes = names(d))
dag_avg_hc_mmhc <- empty.graph(nodes = names(d))
dag_avg_tabu_mmhc <- empty.graph(nodes = names(d))
dag_avg_hc_h2pc <- empty.graph(nodes = names(d))

arcs(dag_tabu_h2pc) <- tabu_h2pc
arcs(dag_hc_mmhc) <- hc_mmhc
arcs(dag_tabu_mmhc) <- tabu_mmhc
arcs(dag_hc_h2pc) <- hc_h2pc

arcs(dag_avg_tabu_h2pc) <- avg_tabu_h2pc
arcs(dag_avg_hc_mmhc) <- avg_hc_mmhc
arcs(dag_avg_tabu_mmhc) <- avg_tabu_mmhc
arcs(dag_avg_hc_h2pc) <- avg_hc_h2pc
```

Doing cross-validation

```r
# Loading the caret package
library(caret)

# Creating a function that will return all parameters of the
# predictive performance
performance_fun<-function(obj_str,dat){
  # Calculating the loss
  cv.bic <- bn.cv(dat,bn=get(obj_str), runs = 10,
          algorithm.args = list(score = "bic"))
  bic_loss<-paste0(round(mean(loss(cv.bic)),4)," (",
                   round(sd(loss(cv.bic)),4),")")
  cv.bde <- bn.cv(dat,bn=get(obj_str), runs = 10,
          algorithm.args = list(score = "bde", iss = 1))
  bde_loss<-paste0(round(mean(loss(cv.bde)),4)," (",
                   round(sd(loss(cv.bde)),4),")")
  # Evaluating the model
  xval <- bn.cv(dat,bn=get(obj_str),  loss = "pred-lw",runs=10,
              loss.args = list(target = "pstplonl"))
  pred_loss<-paste0(round(mean(loss(xval)),4)," (",
                    round(sd(loss(xval)),4),")")

  OBS <- unlist(lapply(xval[[1]], `[[`, "observed"))
  PRED <- unlist(lapply(xval[[1]], `[[`, "predicted"))
  # Loading all of the attributes of the predictive performance
  ress<-confusionMatrix(table(OBS, PRED))$byClass%>%as.data.frame(.)
```

```r
  ress$"."<-round(ress$".",4)
  names(ress)<-obj_str
  # Binding with the loss
  ress<-rbind(ress,bic_loss)%>%rbind(.,bde_loss)%>%rbind(.,pred_loss)
  row.names(ress)[12:14]<-c("BIC loss","BDE loss","Prediction loss")
  # Returning the result
  return(ress)
}
# Loading the names of the structures
names<-c("stru_tabu","stru_hc","stru_mmhc","stru_h2pc",
         "avg.diff_tabu","avg.diff_hc","avg.diff_mmhc","avg.diff_h2pc",
         "dag_tabu_h2pc","dag_tabu_mmhc","dag_hc_mmhc","dag_hc_h2pc",
         "dag_avg_tabu_h2pc","dag_avg_tabu_mmhc","dag_avg_hc_mmhc",
         "dag_avg_hc_h2pc")
# Applying the function to each of the structures
result<-lapply(names,FUN=function(x) performance_fun(x,d))
df_res <- do.call("cbind", result)
df_res$rows_n<-rownames(df_res)
# Saving the performance results
write_csv(as.data.frame(df_res),file="precision.csv")
```

Testing the performance of the models on synthetic data

```r
# Loading needed packages

library(tidyr)
library(dplyr)
library(reshape2)# to reshape the dfs
library(ggplot2)# to visualise
library(viridis)# color palette
library(ggpubr)

# Defyning the function that applies a list of algorithms to the data
myfun<-function(df){
  #list of algorithms
  bn_alg<-list(tabu,hc,h2pc,mmhc)
  #score-based algorithms are tabu and hc
  #hybrid algorithms are h2pc and mmhc

  #return list of structures
  return(lapply(bn_alg, function(x) x(df)))
}

# Defyning a function that returns the percentage of true-positive arcs
percent_true<-function(str_list,arcs_list){
                return(lapply(str_list[1:4],
                  function(x) nrow(semi_join(data.frame(arcs_list),
                        data.frame(arcs(x))))/nrow(arcs_list)))}
# How many arcs are identified
n_arcs_ident<-function(str_list){
                  return(lapply(str_list[1:4],
```

```
                        function(x) nrow(data.frame(arcs(x)))))}
# Defyning the function that adds noice to the data
add_noise<-function(df,percent_noise){
                n_df<-nrow(df)
                for (i in c((n_df+1):(n_df+n_df/100*percent_noise))){
                df[i,]<-
                  lapply(c(1:ncol(df)),
                          function(x)
                          sample(unique(df[,x]),1))
                }

                return(df)
}

myfun3<-function(df,arcs1,arcs2){
  #fist of all, we have to recode the variables so, lavaan can work
  #with the data

  #due to the fact that levels are coded as "no" and "yes", we have to
  #recode all "no" to 0 and all "yes" to 1
  for (i in (1:ncol(df))){
    levels(df[,i])<-c(0:(length(levels(df[,i]))-1))
  }



  #coding variables as numeric
  for (i in 1:ncol(df)){
    df[,i]<-as.numeric(as.character(df[,i]))
  }

  #uploading the model
  base_arcs<-data.frame(semi_join(arcs1,arcs2))



  #building the sem model
  build_model<-function(base_arcs){
    mod0<-"\n"
    for (i in unique(base_arcs$to)){
      mod0<-paste(mod0,i,"~",
      paste0(base_arcs$from[which(base_arcs$to==i)],collapse ="+"),"\n")
    }
    return(mod0)
  }
  mod0<-build_model(base_arcs)


  # evaluating the model
  m0 <- lavaan::sem(mod0, data=df)
base_arcs<-lavaan::standardizedSolution(m0) %>%
    dplyr::filter(!is.na(pvalue)) %>%
    arrange(desc(pvalue)) %>%
```

```
  mutate_if("is.numeric","round",3) %>%
  select(-ci.lower,-ci.upper,-z) %>%
  dplyr::filter(pvalue<0.05)%>%
  dplyr::filter(op=="~")%>% select(rhs,lhs)
names(base_arcs)<-c("from","to")

routs_to_check<-anti_join(arcs1,arcs2)

base_model<-m0

if (nrow(routs_to_check)!=0){
# finding the best model
repeat{

checked_arcs<-base_arcs #saving those arcs that were checked
# This is important to stop the loop in time

# Building more complex models
m_list<-list()
for (i in 1:nrow(routs_to_check)){
  # building a new model
  assign(paste0("new_arc_set",i),rbind(base_arcs,routs_to_check[i,]))
  assign(paste0("mod",i),build_model(get(paste0("new_arc_set",i))))

  assign(paste0("m",i),lavaan::sem(get(paste0("mod",i)), data=df))
  # estimating the model
  m_list<-append(m_list,get(paste0("m",i)))
}

# Adding paths one by one and comparing the models with ANOVA
for (i in 1:length(m_list)){
  anova_res<-anova(m_list[[i]],base_model)
  if(is.na(anova_res["m_list[[i]]",]$`Pr(>Chisq)`)&
     (anova_res["m_list[[i]]",]$Chisq<anova_res["base_model",]$Chisq)){
    # The models are similar, but a more complex one is still a little
    # bit better
      base_model<-m_list[[i]]
      base_arcs<-get(paste0("new_arc_set",i))
    }else{
      if(!(is.na(anova_res["anova_res",]$`Pr(>Chisq)`))&
         !is.na(anova_res["base_model","Pr(>Chisq)"])&
                anova_res["base_model","Pr(>Chisq)"]<0.05){
        # A more complex model is significantly better
        base_model<-m_list[[i]]
        base_arcs<-get(paste0("new_arc_set",i))
      }else{
        if(anova_res["m_list[[i]]","AIC"]<anova_res["base_model","AIC"]&
           anova_res["m_list[[i]]","BIC"]<anova_res["base_model","BIC"]){
          # if none of the conditions is true, a more complex model
          # can still substantially increase the predictability
          # So, if both AIC and BIC are smaller than those of the
          # base model, we can still vote for the more complex one.
          # Especially, when it comes to the social phenomena models
```

```
          base_model<-m_list[[i]]
          base_arcs<-get(paste0("new_arc_set",i))
        }
                  }
      }
  }

  # which arcs still need to be checked
  routs_to_check<-anti_join(routs_to_check,base_arcs)
   # if all of the arcs were checked, stop
   if(identical(checked_arcs,base_arcs)|(nrow(routs_to_check)==0)){
    break
   }
  }}
  # return the best model
  return(base_arcs)
}

# Defyning the function that fine-tunes the BN structures
myfun3<-function(df,arcs1,arcs2){
  #fist of all, we have to recode the variables so, lavaan can work
  #with the data

  #due to the fact that levels are coded as "no" and "yes", we have to
  #recode all "no" to 0 and all "yes" to 1
  for (i in (1:ncol(df))){
    levels(df[,i])<-c(0:(length(levels(df[,i]))-1))
  }



  #coding variables as numeric
  for (i in 1:ncol(df)){
    df[,i]<-as.numeric(as.character(df[,i]))
  }

  #uploading the model
  base_arcs<-data.frame(semi_join(arcs1,arcs2))



  #building the sem model
  build_model<-function(base_arcs){
    mod0<-"\n"
    for (i in unique(base_arcs$to)){
      mod0<-paste(mod0,i,"~",
      paste0(base_arcs$from[which(base_arcs$to==i)],collapse ="+"),"\n")
    }
    return(mod0)
  }
  mod0<-build_model(base_arcs)
```

```r
# evaluating the model
m0 <- lavaan::sem(mod0, data=df)

# choosing only significant paths
base_arcs<-lavaan::standardizedSolution(m0) %>%
  dplyr::filter(!is.na(pvalue)) %>%
  arrange(desc(pvalue)) %>%
  mutate_if("is.numeric","round",3) %>%
  select(-ci.lower,-ci.upper,-z) %>%
  dplyr::filter(pvalue<0.05)%>%
  dplyr::filter(op=="~")%>% select(rhs,lhs)
names(base_arcs)<-c("from","to")

routs_to_check<-anti_join(arcs1,arcs2)

base_model<-m0
if (nrow(routs_to_check)!=0){
# finding the best model
repeat{

checked_arcs<-base_arcs #saving those arcs that were checked
# This is important to stop the loop in time

# Building more complex models
m_list<-list()
for (i in 1:nrow(routs_to_check)){
  # building a new model
  assign(paste0("new_arc_set",i),rbind(base_arcs,routs_to_check[i,]))
  assign(paste0("mod",i),build_model(get(paste0("new_arc_set",i))))

  assign(paste0("m",i),lavaan::sem(get(paste0("mod",i)), data=df))
  # estimating the model
  m_list<-append(m_list,get(paste0("m",i)))
}

# Adding paths one by one and comparing the models with ANOVA
for (i in 1:length(m_list)){
  if (slot(m_list[[i]], "optim")$converged==T){
  anova_res<-anova(m_list[[i]],base_model)
  if(is.na(anova_res["m_list[[i]]",]$`Pr(>Chisq)`)&
     (anova_res["m_list[[i]]",]$Chisq<anova_res["base_model",]$Chisq)){
    # The models are similar, but a more complex one is still a little
    # bit better
      base_model<-m_list[[i]]
      base_arcs<-get(paste0("new_arc_set",i))
    }else{
      if(!(is.na(anova_res["anova_res",]$`Pr(>Chisq)`))&
         !is.na(anova_res["base_model","Pr(>Chisq)"])&
                anova_res["base_model","Pr(>Chisq)"]<0.05){
        # A more complex model is significantly better
        base_model<-m_list[[i]]
        base_arcs<-get(paste0("new_arc_set",i))
      }else{
```

```r
          if(anova_res["m_list[[i]]","AIC"]<anova_res["base_model","AIC"]&
             anova_res["m_list[[i]]","BIC"]<anova_res["base_model","BIC"]){
            # if none of the conditions is true, a more complex model
            # can still substantially increase the predictability
            # So, if both AIC and BIC are smaller than those of the
            # base model, we can still vote for the more complex one.
            # Especially, when it comes to the social phenomena models
          base_model<-m_list[[i]]
          base_arcs<-get(paste0("new_arc_set",i))
        }
                  }}
      }
  }

  # which arcs still need to be checked
  routs_to_check<-anti_join(routs_to_check,base_arcs)
   # if all of the arcs were checked, stop
   if(identical(checked_arcs,base_arcs)|(nrow(routs_to_check)==0)){
    break
   }
  }}
  # return the best model
  return(base_arcs)
}

# Defyning the function that fine-tunes every combination of algorithms
my_fun4<-function(df,list_res,list_ident,
                  list_ident2,sample_n,arcs_list){

  bn_alg4<-list("tabu","hc","h2pc","mmhc")
  n_arcs_samples<-paste0("s",sample_n,"_n_arcs")
  percent_samples<-paste0("s",sample_n,"_percent")


  for (i in 1:length(df)){

    # check which combination of the algorithms work better
    for (j in 1:2){
      for (k in 3:4){
        cat("i is ",i," j is ",j, "k is ",k)
         best_model_arcs<-myfun3(df[[i]],
                        data.frame(arcs(list_ident[[i]][[j]])),
                        data.frame(arcs(list_ident[[i]][[k]])))

         # adding a new line to the list of the results
         list_res[[i]][nrow(list_res[[i]])+1,"algorithm"]<-
           paste0(bn_alg4[j],"+",bn_alg4[k],"+SEM")


          # how many arcs identified
          list_res[[i]][nrow(list_res[[i]]),"n_arcs"]<-
            nrow(best_model_arcs)
```

```
        # percentage of arcs identified correctly
        list_res[[i]][nrow(list_res[[i]]),"percent"]<-
          nrow(semi_join(data.frame(arcs_list),
                         best_model_arcs))/nrow(arcs_list)

        for (l in 1:length(n_arcs_samples)){
          cat("l is ",l)
          best_model_arcs<-myfun3(df[[i]],
                       data.frame(arcs(list_ident2[[i]][[l]][[j]])),
                       data.frame(arcs(list_ident2[[i]][[l]][[k]])))

           # how many arcs identified
        list_res[[i]][nrow(list_res[[i]]),n_arcs_samples[[l]]]<-
          nrow(best_model_arcs)

        # percentage of arcs identified correctly
        list_res[[i]][nrow(list_res[[i]]),percent_samples[[l]]]<-
          nrow(semi_join(data.frame(arcs_list),
                         best_model_arcs))/nrow(arcs_list)
        }
      }
    }


  }
  return(list_res)
}

# Defyning the function that visualises the results
vis_fun<-function(list_res2,percent_noise,sample_n,arcs_list,df_name){
  sets<-c("Without noise",paste0(percent_noise,"% of noise"))

 vis_df<-list_res2[[1]][0,]
  for (i in 1:length(list_res2)){
    temp<-list_res2[[i]]
    temp$set<-sets[i]
    temp$fp1<-(temp$n_arcs-(temp$percent*nrow(arcs_list)))
    for (j in 1:length(sample_n)){
      temp[,paste0("fp",
                  sample_n[[j]])]<-temp[,paste0("s",
                                      sample_n[[j]],
                                      "_n_arcs")]-(temp[,paste0("s",
                                      sample_n[[j]],
                                      "_percent")]*nrow(arcs_list))
    }
    vis_df<-rbind(vis_df,temp)
  }



  vis_df1<-vis_df[,c("algorithm","percent",
                     paste0("s",sample_n,"_percent"),"set")]
  names(vis_df1)[2:(length(names(vis_df1))-1)]<-c("1 structure",
```

```
                                             paste0(sample_n,
                                                    " structures"))

vis_df1<-melt(vis_df1, id.vars = c("algorithm","set"))
vis_df1$algorithm <- factor(vis_df1$algorithm,
                            # Reordering group factor levels
                       levels = unique(vis_df1$algorithm))
vis_df1$variable <- factor(vis_df1$variable,
                           # Reordering group factor levels
                       levels = rev(paste0(sample_n," structures")))
vis_df1$set <- factor(vis_df1$set,
                      # Reordering group factor levels
                       levels = sets)
vis_df1 <-vis_df1[with(vis_df1, order(set,algorithm,variable)),]


g_accur<-ggplot(data=vis_df1, aes(x=variable,
                                  y=value,
                                  color=algorithm,
                                  fill=algorithm)) +
geom_bar(stat="identity")+
facet_grid(algorithm~set,switch="y")+
coord_flip()+
scale_colour_viridis_d()+
scale_fill_viridis_d()+
  theme(panel.background = element_rect(fill = NA),
      legend.position = "none")+
ylab(expression(frac(Number~of~the~correctly~identified~arcs,
                     Number~of~the~original~structure~arcs)))+
xlab("Model has been averaged over")


vis_df2<-vis_df[,c("algorithm","fp1",paste0("fp",sample_n),
                       "set")]
names(vis_df2)[2:(length(names(vis_df2))-1)]<-c("1 structure",
                                             paste0(sample_n,
                                                    " structures"))

vis_df2<-melt(vis_df2, id.vars = c("algorithm","set"))
vis_df2$algorithm <- factor(vis_df2$algorithm,
                            # Reordering group factor levels
                       levels = unique(vis_df2$algorithm))
vis_df2$variable <- factor(vis_df2$variable,
                           # Reordering group factor levels
                       levels =  rev(paste0(sample_n," structures")))
vis_df2$set <- factor(vis_df2$set,
                      # Reordering group factor levels
                       levels = sets)
vis_df2 <-vis_df2[with(vis_df2, order(set,algorithm,variable)),]
g_fp<-ggplot(data=vis_df2, aes(x=variable, y=value,
                               color=algorithm, fill=algorithm)) +
geom_bar(stat="identity")+
facet_grid(algorithm~set,switch="y")+
```

```r
  coord_flip()+
  scale_colour_viridis_d()+
  scale_fill_viridis_d()+
    theme(panel.background = element_rect(fill = NA),
        legend.position = "none")+
 ylab("Number of the false positive arcs")+
  xlab("Model has been averaged over")


  myplot<-ggarrange(g_accur,g_fp,
          ncol = 1, nrow = 2)
  ggsave(paste0(df_name,"_comparison.eps"), plot = myplot,
    scale = 1, width =50,
    height = 75, units =  "cm")
  return(list_res2)
}


# Defyning the function that saves the results
my_big_fun<-function(df,arc_list,df_name,
                     percent_noise=c(0.5,1,2,5,10,15),
                     sample_n=c(50,200,500)){

  # adding noise
  set.seed(43)
  df2<- append(list(df),lapply(percent_noise,
                               function(x) add_noise(df,x)))

  # I will save the results in a list of dfs
  res <- data.frame(matrix(ncol = 1+length(sample_n)*2+2, nrow = 4))
  names(res)<-c("algorithm",
              "percent",
              "n_arcs",
              paste0("s",sample_n,"_percent"),
              paste0("s",sample_n,"_n_arcs"))
  res[1:4,1]<-c("tabu","hc","h2pc","mmhc")

  #learning the structure on the updated data
  list_ident<-lapply(df2,function(x) myfun(x))
  # how many correct arcs are identified
  list_percent<-lapply(list_ident, function(x) percent_true(x,arc_list))
  # how many arcs are identified
  list_n_arcs_ident<-lapply(list_ident, function(x) n_arcs_ident(x))

  list_res<-list()
  for (i in 1:length(list_percent)){
    res2<-res
    res2$percent<-unlist(list_percent[[i]], recursive=FALSE)
    res2$n_arcs<-unlist(list_n_arcs_ident[[i]], recursive=FALSE)
    list_res<-append(list_res,list(res2))
  }
```

```
  # averaging on 50, 200 and 500 samples
  list_ident2<-lapply(df2,
                 function(i) lapply(sample_n,
                                    function(x) myfun2(i,x)))
  # how many correct arcs are identified
  list_percent<-lapply(list_ident2,
                   function(i) lapply(i,
                                  function(x)
                                    percent_true(x,arc_list)))
  # how many arcs are identified
  list_n_arcs_ident<-lapply(list_ident2,
                       function(i) lapply(i,
                                  function(x) n_arcs_ident(x)))

  for (i in 1:length(list_percent)){
    res2<-list_res[[i]]
    res2$s50_percent<-unlist(list_percent[[i]][[1]], recursive=FALSE)
    res2$s200_percent<-unlist(list_percent[[i]][[2]], recursive=FALSE)
   res2$s500_percent<-unlist(list_percent[[i]][[3]], recursive=FALSE)
    res2$s50_n_arcs<-unlist(list_n_arcs_ident[[i]][[1]],
                       recursive=FALSE)
    res2$s200_n_arcs<-unlist(list_n_arcs_ident[[i]][[2]],
                        recursive=FALSE)
    res2$s500_n_arcs<-unlist(list_n_arcs_ident[[i]][[3]],
                        recursive=FALSE)
    list_res[[i]]<-res2
  }

  list_res2<-my_fun4(df2,list_res,list_ident,
                 list_ident2,sample_n,arc_list)

  list_res2<-vis_fun(list_res2,percent_noise,sample_n,arc_list,df_name)
  save(list_res2,file=paste0(df_name,"1_res.RData"))
  return(list_res2)
}
```

Applying all of the functions to the data

```
#Loading the "ALARM" dataset
data(alarm)

# Loading the true model-structure
modelstring = paste0("[HIST|LVF][CVP|LVV][PCWP|LVV][HYP][LVV|HYP:LVF][LVF]",
  "[STKV|HYP:LVF][ERLO][HRBP|ERLO:HR][HREK|ERCA:HR][ERCA][HRSA|ERCA:HR][ANES]",
  "[APL][TPR|APL][ECO2|ACO2:VLNG][KINK][MINV|INT:VLNG][FIO2][PVS|FIO2:VALV]",
  "[SAO2|PVS:SHNT][PAP|PMB][PMB][SHNT|INT:PMB][INT][PRSS|INT:KINK:VTUB][DISC]",
  "[MVS][VMCH|MVS][VTUB|DISC:VMCH][VLNG|INT:KINK:VTUB][VALV|INT:VLNG]",
  "[ACO2|VALV][CCHL|ACO2:ANES:SAO2:TPR][HR|CCHL][CO|HR:STKV][BP|CO:TPR]")
dag = model2network(modelstring)
arc_list<-arcs(dag)
my_big_fun(alarm,arc_list,"alarm",
```

```
                    percent_noise=c(0.5,1,2,5,10,15),
                    sample_n=c(50,200,500))
```
