# Supplementary material for: Predicting online participation through Bayesian network analysis
Source: PLoS One. 2021 Dec 23;16(12):e0261663. doi: 10.1371/journal.pone.0261663 (PMC8699968; doi:10.1371/journal.pone.0261663)
Supplement: Attachment — Submitted filename: Response to reviewers.pdf [file pone.0261663.s011.pdf]

Response to the comments about the submitted paper *Predicting online participation through Bayesian network analysis*

November 5, 2021

I thank the reviewers and the associate editor for their constructive comments. I have addressed all of them and modified the paper accordingly. Please, find modified parts of the text to have different colors. Blue color represents changes done according to the comments of Reviewer 1. Red color represents changes done according to the comments of Reviewer 2. The detailed answers follow.

Please note that reviewers' comments are in italics while the answers are not.

Answers to Reviewer 1

Comment R1.1 *Comment:*
As the authors rightly point out, the structure of a Bayesian network can be affected by imprecise or missing observations.
In this regard, in the Results section, I found no information regarding missing data. How much did missing data were there?, on which variables?
Regarding measurement imprecision, it would be useful to clarify at least for the most important variables (online participation, political participation, political participation, etc.) how these variables were measured in the ESS.

Answer to R1.1 Answer:
Please, find an additional table (Table 1) describing the data used for the analysis and showing the original values of the variables. I have also, added Table 3 showing step-by-step how the data was transformed for the analysis, in particular, how it was recoded, what were the values after dimensionality reduction and how those values were discretized. Table 3 also, shows the number of missing values for each one of the variables. In addition to that, I wrote a short paragraph, where I elaborate a little bit more on the variables used for the analysis (see pp. 3-4 in red).

Comment R1.2 *Comment:*
At the structure learning stage:
A typical issue that needs to be dealt with is selecting the best structure for the Bayesian network.
In this process, as mentioned by the authors, it is possible to use several algorithms. They use score-based and hybrid algorithms. Is it possible to report a table with more accurate information or some statistics of the goodness-of-fitness of the algorithms to compare the performance?
On the other hand, as a means of robustness, I understand, they use several learned structures to predict the variable "online participation." The idea is to evaluate these models to get the best of them.
Based on what performance measure do the authors evaluate the predictive ability?
In summary, it would be nice to explain how they found the best set of network structures in more detail...[further] how do you prevent the problem of overfitting? do you use any cross-validation scheme?...[further]
The results section reports the results of the directed acyclic graphs. The section reports the Edges present in the structures and that are influential in online activism. It also reports the inference parameters (conditional probability distributions). However, I do not find results concerning "prediction ." In this regard, I would like to differentiate between the knowledge we gain in interpreting the network structure and the ability of this model to predict.
What happens with the model's performance (Bayesian network learned) to predict the online activism variable in a different sample than the one used for the learning process?
My understanding is that the suggested three-step approach allows adding robustness and validity to the results, especially for survey data. However, I wonder how much the predictive capacity increases between a model found with this methodology and another without this methodology. This is of interest because the title of this paper is precisely related to the "prediction" of online participation.

Answer to R1.2 Answer:
Please, find a new Table 4 with the comparison of the models' predictive performance in the main manuscript. I have also updated the part of the Methods section on pp. 8-10 in blue in regard to this table and the comparison of the models. Comparing the performance of several models, I focused on maximising the recall (i.e., the performance measure that shows how many, out of all the positive cases, have been predicted correctly) and balanced accuracy, as well as minimising the prediction error and other types of losses. Moreover, I have tested the ensemble learning method on a synthetic dataset to show that the method indeed, substantially decreases the number of false positive arcs usually found by score-based algorithms. The data presented by the ALARM dataset (Beinlich et al., 1989) resembles the one analysed in this paper in regard to two parameters: (1) it has a comparable number of observations (20 000) and the number of variables is, also, quite high (37 variables). Still, the ALARM dataset has a higher complexity of the data as the variables often have more than 2 values and the number of variables is higher. Thus, the accuracy of the models is decreased substantially when compared to the predictive performance of the models tested on the ESS data. Yet, the test shows that balancing the structures substantially reduces over-fitting while keeping the predictive performance comparatively high. Moreover, the test once again, showed that balancing TABU and H2PC, in particular, allows reaching both, high predictive performance and low over-fitting. This result is in line with the conclusions of Constantinou et al. (2020), who in several tests, showed TABU and H2PC to be the top BN structure learning algorithms.

Comment R1.3 *Comment:*
According to this, step 2 (SEM) identifies those edges or arcs of the Bayesian network that are statistically significant.
If we assume that we are only interested in the model that performs best in predicting the dependent variable, then what do we gain from step 2?
I assume that the authors are not only interested in predictive ability, but also in "explaining" the online participation variability between observations.

Answer to R1.3 Answer:
Indeed, one of the main objectives is to find a model that can serve as a theoretical foundation for future studies on online political participation predictors. In that regard, ensemble learning shows to be important when trying to decrease the number of false positives and learn the theoretically-sound structure. Please, find those concerns to be elaborated upon on pp. 9-10 of the main manuscript.

Comment R1.4 *Comment:*
From the Bayesian network, we obtain causality information. In SEM we see if there is statistical significance in the network arcs. So how do we interpret causality between two variables that are not significant? How do we interpret a pair of variables whose relationship is significant but without connecting arcs in the network?

Answer to R1.4 Answer:
Regarding the first question, such a situation can indeed, take place. However, it is only applicable to the marginal cases with weak coefficients. Due to the fact that every algorithm is specific in dealing with marginal cases, some of them can keep the arcs showing a rather weak relationship between the variables. This is due to the fact that some algorithms (e.g., score-based) prioritise the predictive performance of the model. In the meanwhile, maximum likelihood is more restrictive. When using SEM, less significant effects are being discarded.
Concerning the second question, if in theory, this situation is possible, it is still not applicable to the proposed method. This is due to the fact that the first step of the analysis is BN structure learning and the second is SEM and not the other way around. The purpose of exploring the structure of the network using BN structure learning algorithms is to limit the number of possible combinations of relationships between the variables. Starting with SEM would require knowing the structure of the model in advance, which is not possible to do examining the question of preconditions of political participation due to the fact only a limited number of experimental studies examined the question of political participation factor causality (e.g., Rothstein and Eek, 2009; Hernández-Lagos and Minor, 2020).

Comment R1.5 *Comment:*
In the results section on page 8, Lines 289-301, the authors report the sensitivity. They indicate how much online participation could increase or decrease when the level of other variables changes. I believe that much could be gained from the results by specifying a more detailed sensitivity analysis that would allow us to understand the most critical factors on online participation on the dependent variable in terms of intensity and direction.

Answer to R1.5 Answer:
Thank you so much for this comment and your suggestion to provide the results of a more detailed sensitivity analysis! After careful consideration and research on the topic of sensitivity analysis of Bayesian network models, I came to the conclusion that providing another (than what was presented in Fig 5) graphical result of sensitivity analysis, would not give any additional benefit. Moreover, a traditional way to report sensitivity of a node in Bayesian networks (as reported in Leonelli et al., 2021), includes calculating the middle values of the variables, which were reported to have a binary scale. In that regard, it feels like it may be confusing for a reader to read such statistics, knowing that all of the variables were discretized. For example, the following graph (i.e., Figure 1) shows how the results of the proposed sensitivity analysis may be reported.

Such visual representation of the results may suggest, however, that variable Internal political efficacy have a continuous scale and the values range from 0 to 1. Which is not in line with what was reported earlier.
On the other hand, the solution, which was earlier proposed in the manuscript, i.e., Fig 5 (I will once again provide it here as Figure 2), provides the same statistics but shows internal political efficacy to be a variable on a binary scale (with values low and high internal political efficacy).

Moreover, the sensitivity of online political participation represented as percentage bars may be easier to understand for a reader, who has little knowledge about sensitivity analysis.

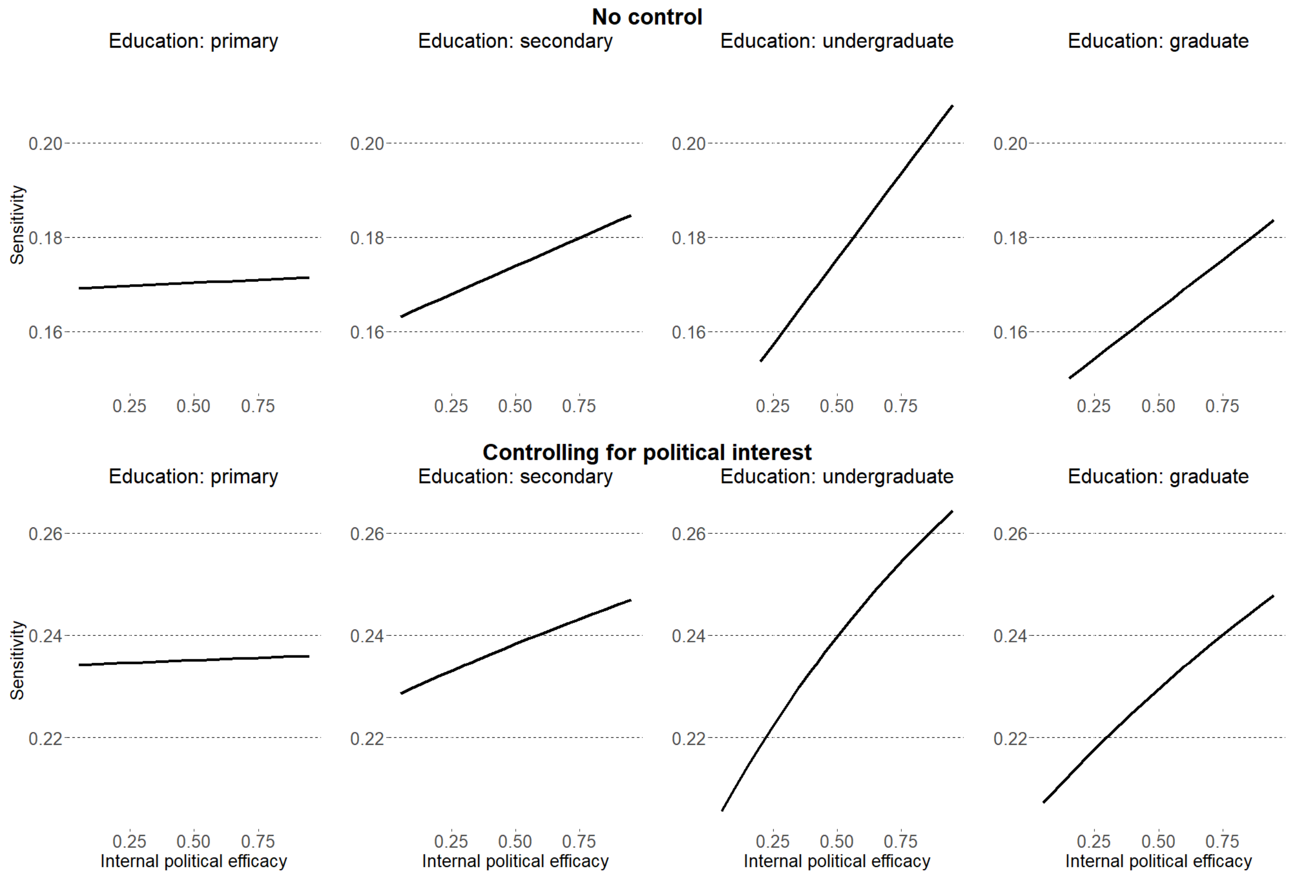

Figure 1: **Sensitivity of the node "online political participation".** *Source: ESS round 9: European social survey round 9 data.Data file edition 1.2.* (2018). N= 27 379 individuals in 19 countries. *Notes:* Bayesian parameter estimation, conditional on the acquired structure of the network, was applied to analyze the data.

Comment R1.6 *Comment:*
This seems obvious, but for a reader not so knowledgeable in Bayesian networks, it might be nice to interpret the directionality (causality) of the Bayesian network edges.

Answer to R1.6 Answer:
Please, find some clarification added to the text of the main manuscript on p. 11 in blue.

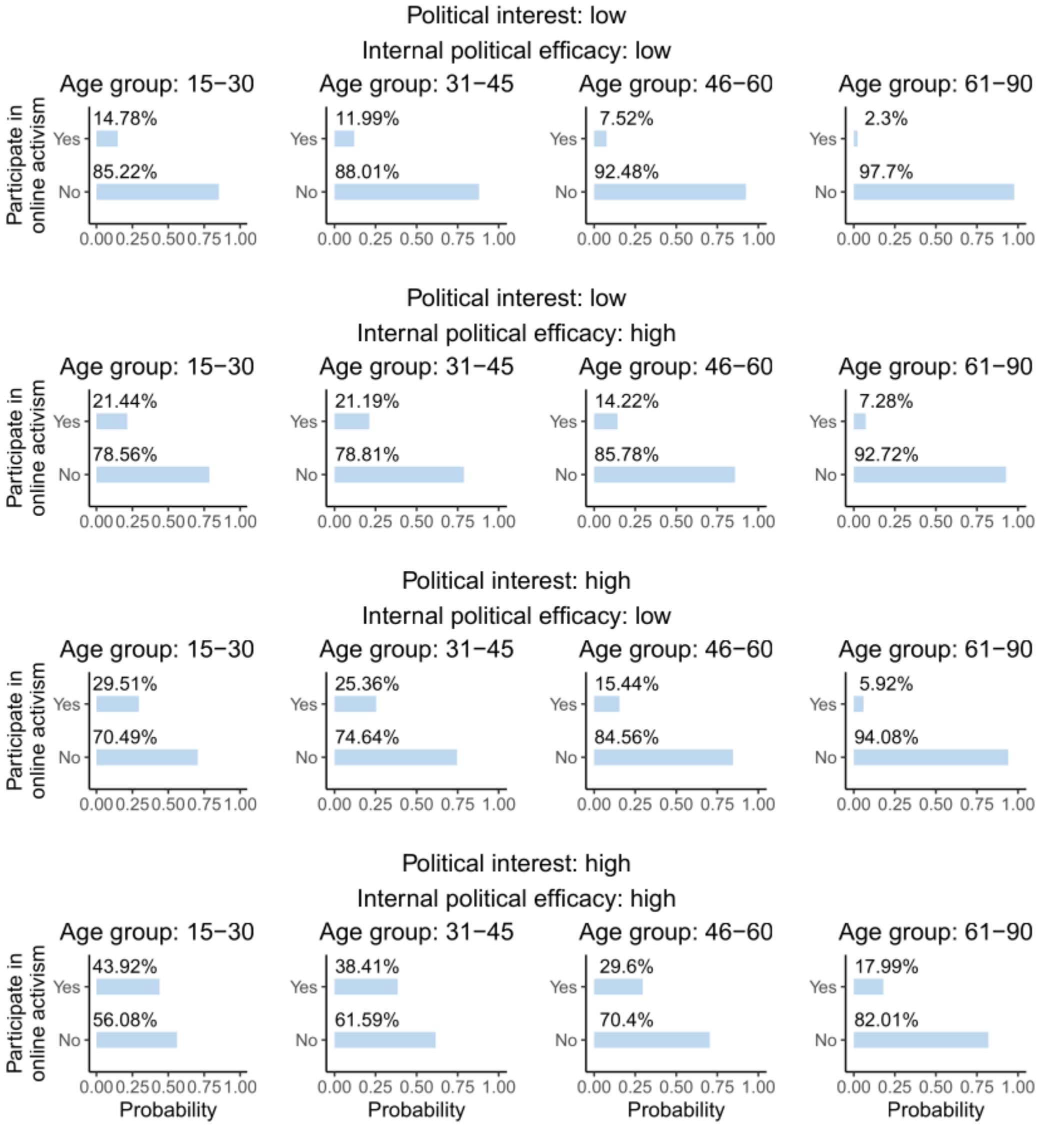

Figure 2: **Probability distribution of all factors associated with participation in online activism.** *Source: ESS round 9: European social survey round 9 data.Data file edition 1.2.* (2018). N= 27 379 individuals in 19 countries. *Notes:* Bayesian parameter estimation, conditional on the acquired structure of the network, was applied to analyze the data. Entities are the probabilities of events in percentage. The following conditional probability query was applied: education is "graduate", placement on the left-right scale is "left", work in an NGO is "yes", political trust is "low" and income is "low".

Answers to Reviewer 2

Comment R2.1 *Comment:*
clarify which algorithm was used for the discretization

Answer to R2.1 Answer:
Please, find a new table (Table 3) describing how all of the variables were discretized added to the main text of the manuscript. Table 3 shows how the data was transformed for the analysis, in particular, how it was recoded, what were the values after dimensionality reduction and how those values were discretized.

Comment R2.2 *Comment:*
Step 1 should include more information on Bayesian networks. mathematical model, different networks and a mathematical description of the algorithms used. https://www.sciencedirect.com/science/article/pii/S0167739X19303322 https://link.springer.com/article/10.1023/A:1007465528199

Answer to R2.2 Answer:
Please, find a more detailed description of the algorithms and the mathematical formula of the Bayesian net-

work structure learning in the main manuscript on p.8 in red. Please, note that the Methods section does not include the mathematical formula for each one of the algorithms due to the text-length constraints as there are 7 algorithms that are being applied to the data and each one of them uses its own approach to maximisation of the formula

$$Pr(G|D) \propto Pr(G)Pr(D|G) = Pr(G) \int Pr(D|G,\Theta)Pr(\Theta|G)d\Theta$$

, where D is a dataset, $B = (G, \Theta)$ is a Bayesian network, in which the parameters of the global distribution of a set of variables $X = X_1, X_2, ..., X_p$ is denoted as Θ and G is a directed acyclic graph, $Pr(G|D)$ is the posterior probability of the DAG, $Pr(G)$ is the product of the prior distribution over the possible DAGs and $Pr(D|G)$ is the probability of the data (Scutari and Denis, 2014).
Still, I have tried to elaborate on the main principles of score-based, constraint-based and hybrid algorithms in a way that is understandable for a reader. I have also, tried to refer an interested reader to the original works of scholars, who developed the algorithms.
Thank you for the suggested resources showing good examples of how to mathematically define the concepts, which were elaborated upon in the manuscript! The suggested literature, however, uses BN for classification tasks. In the meanwhile, this article addresses the question of prediction and the algorithms, which were compared in the paper, deal with the task of BN structure learning, rather than classification (which makes it harder to reference such literature in the manuscript).
Comment R2.3 *Question:*
I do not understand how the authors choose the high scores of the networks. what is this? what is the class label used in the Bayesian network?

Answer to R2.3 Answer:
As mentioned earlier, the BN structure learning algorithms aim to find the model that maximises the above-mentioned formula. All structure learning algorithms approach the task of formula maximisation differently. Score-based algorithms apply heuristic optimisation techniques assigning to each structure candidate a network score (i.e., Bayesian Information criterion (BIC) (Schwarz, 1978) or Bayesian Dirichlet equivalent (BDE) uniform (Heckerman et al., 1995) scores), which shows the model's goodness of fit, and trying to maximise it.
Due to the fact that within this revision, comparing the structures, I focus more on the predictive performance of the models rather than the network scores, I see no additional benefit to elaborate on the network scores in the manuscript. The network scores are defined by complex mathematical formulas, which an interested reader can find in the original works of Schwarz (1978) and Heckerman et al. (1995). Those works were referred to in the main manuscript.
In regard to the class labels, as mentioned earlier, the algorithms are used to learn the structures of the BN, rather than for a classification task. Thus, score-based and hybrid algorithms use network scores (BIC and BDE) to evaluate the structure's goodness of fit.

Comment R2.4 *Comment:*
Authors should include more details of the data used, especially when it is used in the Bayesian network, it is not clear.

Answer to R2.4 Answer:
I have included a description of the variables used for the analysis to the main text of the manuscript. Please, find this information on pp. 3-4 in red. I have also, added a detailed description of the variables as Table 1 of the main manuscript.

References

Beinlich, I. A., Suermondt, H. J., Chavez, R. M. and Cooper, G. F. (1989), The ALARM monitoring system: A case study with two probabilistic inference techniques for belief networks, *in* J. Hunter, J. Cookson and J. Wyatt, eds, 'AIME 89', Springer Berlin Heidelberg, Berlin, Heidelberg, pp. 247–256.

Constantinou, A. C., Liu, Y., Chobtham, K., Guo, Z. and Kitson, N. K. (2020), 'Large-scale empirical validation of bayesian network structure learning algorithms with noisy data'.

ESS round 9: European social survey round 9 data.Data file edition 1.2. (2018), NSD - Norwegian Centre for Research Data, Norway – Data Archive and distributor of ESS data for ESS ERIC.

Heckerman, D., Geiger, D. and Chickering, D. (1995), 'Learning Bayesian networks: The combination of knowledge and statistical data', *Machine Learning* **20**, 197–243.

Hernández-Lagos, P. and Minor, D. (2020), 'Political identity and trust', *Quarterly Journal of Political Science* **15**(3), 337–367.
URL: *http://dx.doi.org/10.1561/100.00018063*

Leonelli, M., Ramanathan, R. and Wilkerson, R. L. (2021), 'Sensitivity and robustness analysis in bayesian networks with the bnmonitor r package', *ArXiv* **abs/2107.11785**.

Rothstein, B. and Eek, D. (2009), 'Political corruption and social trust: An experimental approach', *Rationality and society* **21**(1), 81–112.

Schwarz, G. (1978), 'Estimating the dimension of a model', *The Annals of Statistics* **6**(2), 461–464.

Scutari, M. and Denis, J.-B. (2014), *Bayesian networks: with examples in R*, CRC press, Boca Raton, FL.
