# Supplementary material for: Predicting online participation through Bayesian network analysis
Source: PLoS One. 2021 Dec 23;16(12):e0261663. doi: 10.1371/journal.pone.0261663 (PMC8699968; doi:10.1371/journal.pone.0261663)
Supplement: Attachment — Submitted filename: Response to reviewers.pdf [file pone.0261663.s012.pdf]

Response to the comments about the submitted paper *Predicting online participation through Bayesian network analysis*

December 3, 2021

I thank the reviewers and the associate editor for their comments. In particular, Reviewer 2 mentioned two specific issues:

1. That "Figure 2 is illegible": I have addressed this issue and improved the quality of the figure;
2. That "Table 3 is very long": I have moved Table 3 to the supplementary material file (S1 File).
